# Secondary Metabolites with Nitric Oxide Inhibition from Marine-Derived Fungus *Alternaria* sp. 5102

**DOI:** 10.3390/md18080426

**Published:** 2020-08-14

**Authors:** Senhua Chen, Yanlian Deng, Chong Yan, Zhenger Wu, Heng Guo, Lan Liu, Hongju Liu

**Affiliations:** 1 School of Pharmacy, Guangdong Medical University, Dongguan 523808, China; dengylian2016@163.com (Y.D.); jdsbj2000@163.com (C.Y.); 2 School of Marine Sciences, Sun Yat-sen University, Guangzhou 510006, China; chensenh@mail.sysu.edu.cn (S.C.); wuzher@mail2.sysu.edu.cn (Z.W.); hengeguo163@163.com (H.G.); cesllan@mail.sysu.edu.cn (L.L.); 3 Southern Laboratory of Ocean Science and Engineering (Guangdong, Zhuhai), Zhuhai 519000, China

**Keywords:** marine-derived fungus, secondary metabolites, *Alternaria*, anti-inflammatory

## Abstract

Two new benzofurans, alternabenzofurans A and B (**1** and **2**) and two new sesquiterpenoids, alternaterpenoids A and B (**3** and **4**), along with 18 known polyketides (**5**−**22**), were isolated from the marine-derived fungus *Alternaria* sp. 5102. Their structures were elucidated on the basis of extensive spectroscopic analyses (1D and 2D NMR, HR-ESIMS, and ECD) and X-ray crystallography, as well as the modified Mosher’s method. Compounds **2**, **3**, **5**, **7**, **9**–**18**, and **20**–**22** exhibited potent anti-inflammatory activity by inhibiting the production of NO in RAW264.7 cells activated by lipopolysaccharide with IC_50_ values in the range from 1.3 to 41.1 μM. Structure-activity relationships of the secondary metabolites were discussed.

## 1. Introduction

Marine-derived fungi are a significant source of pharmacological molecules with interesting and diversified structural properties [1,2,3,4]. Among them, the fungal genus *Alternaria* had a widespread distribution in marine source [4] (including sediment [5], sponge [6], alga [7], mangrove [8], soft coral [9]) and could produce plenty of structural molecules, including nitrogen-containing compounds, steroids, terpenoids, pyranones, quinones, and phenolics [10]. These metabolites exhibited a variety of biological activities such as cytotoxic, antimicrobial, enzyme inhibitor properties [10]. For instances, naphtho-*γ*-pyrones pyrophen displayed anti-fungal activity against *C. albicans* from the marine-derived fungus *Alternaria alternate* D2006 [11], anthraquinone anthrininone A showed significant inhibition activity against indoleamine 2,3-dioxygenase 1 (IDO1) from the deep-sea derived fungus *Alternaria tenuissima* DFFSCS013 [12], drimane meroterpenoid alternarin A revealed effective inhibition of spontaneous synchronous Ca^2+^ oscillations from the coral-associated fungi *Alternaria* sp. ZH-15 [13].

**Figure 1:**
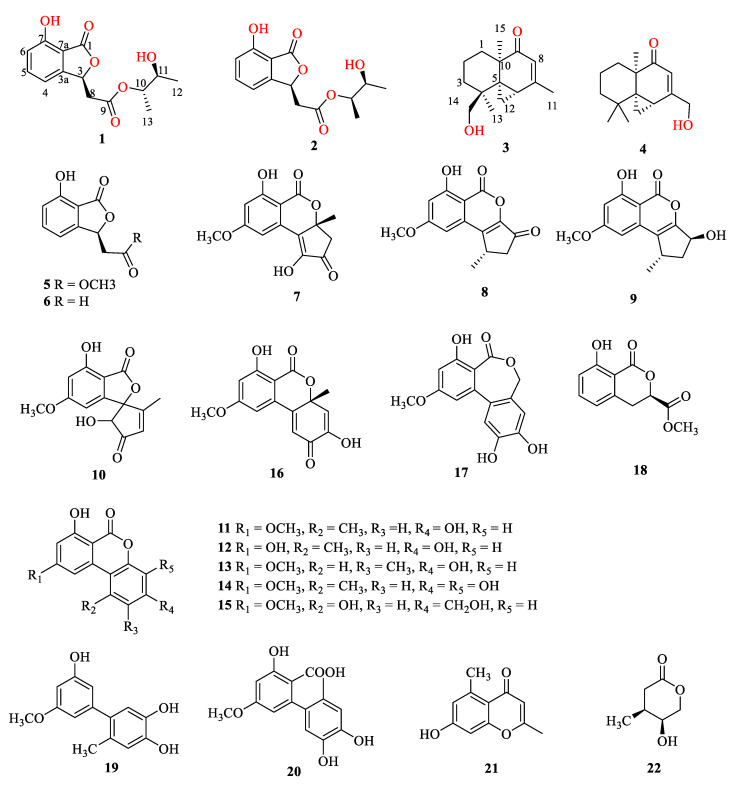
Chemical structures of **1**–**22**.

Recently, we searched for anti-inflammatory secondary metabolites from the South China Sea [14,15,16,17]. EtOAc extract of marine-derived fungus *Alternaria* sp. 5102 showed anti-inflammatory activity in vitro by inhibiting nitric oxide (NO) production in lipopolysaccharide activated in RAW264.7 cells. Subsequent chemical investigation led to the isolation of 22 secondary metabolites, including two new benzofurans, alternabenzofurans A and B (**1** and **2**) and two new sesquiterpenoids, alternaterpenoids A and B (**3** and **4**), along with 18 known polyketides, isobenzofuranone A (**5**) isoochracinic acid (**6**), (*R*)-1,6-dihydroxy-8-methoxy-3a-methyl-3,3a-dihydrocyclopenta[c]isochromene-2,5-dione (**7**), dihydroaltenuenes A (**8**), phialophriol (**9**), ±talaroflavone (**10**), alternariol-9-*O*-methyl ether (**11**), alternariol (**12**), 2-methyl-9-methoxy alternariol (**13**), 3’-hydroxyalternariol 5-*O*-methyl ether (**14**), alternariol-1’-hydroxy-9-methyl ether (**15**), dehydroaltenusin (**16**), alteryulactone (**17**), tenuissimasatin (**18**), 5’-methoxy-6-methyl- biphenyl-3,4,3’-triol (**19**), altenusin (**20**), 2,5-dimethyl-7-hydroxychromone (**21**) and walterolactone C (**22**) (Figure 1). Their structures were identified by extensive spectroscopic analyses (1D and 2D NMR, HR-ESIMS, and ECD) and X-ray crystallography, as well as the modified Mosher’s method. Compounds **2**, **3**, **5**, **7**, **9**—**18**, and **20**—**22** showed potential anti-inflammatory activity with IC_50_ values ranging from 1.3 to 41.1 µM. Herein, we report the isolation, structure determination, and anti-inflammatory bioactivity of the secondary metabolites.

## 2. Results and Discussion

Alternabenzofuran A (**1**) was obtained as a colorless crystal. The molecular formula of **1** was established as C_14_H_16_O_6_ based on the HR-ESIMS data (Figure S1) at *m*/*z* 279.08790 [M-H]^−^ (calcd for C_14_H_15_O_6_, 279.08741), implying seven degrees of unsaturation. The ^1^H NMR spectrum (Figure S2) (Table 1) along with the HSQC experiment showed three aromatic protons owing to a 1,2,3- trisubstituted aromatic ring [*δ*_H_ 7.56 (1H, t, *J* = 7.9 Hz); 6.96 (1H, dd, *J* = 2.5, 7.3 Hz); 6.96 (1H, dd, *J* = 2.5, 7.3 Hz)], three methines [*δ*_H_ 5.90 (1H ,t, *J* = 6.6 Hz); 4.86 (1H, m); 3.73 (1H, m);], one methylene [*δ*_H_ 2.95 (2H, m)], and two methyls [1.25 (3H, d, *J* = 6.4 Hz); 1.18 (3H, d, *J* = 6.4 Hz)]. The ^13^C NMR (Figure S3) and HSQC data (Figure S4) of **1** indicated the presence of 14 carbons for eight sp^2^ hybridized carbons including two ester carbonyls (*δ*_C_ 171.5, 168.8) and six sp^3^ hybridized carbons (*δ*_C_ 78.3, 76.3, 70.1, 39.7, 19.1, 16.4).

**Figure 2:**
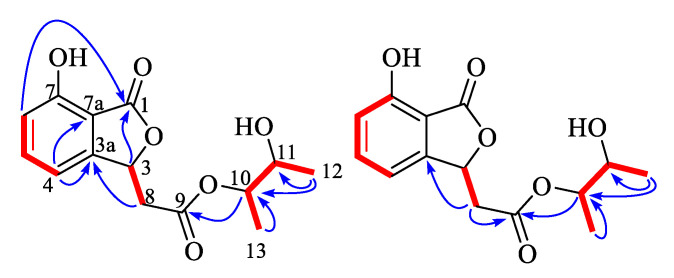
Key ^1^H-^1^H COSY (red line) and HMBC (blue arrow) correlations of compounds **1** and **2**.

**Figure 3:**
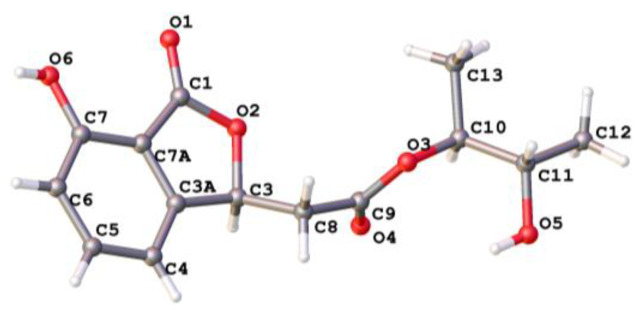
Single crystal structure of **1**.

**Figure 4:**
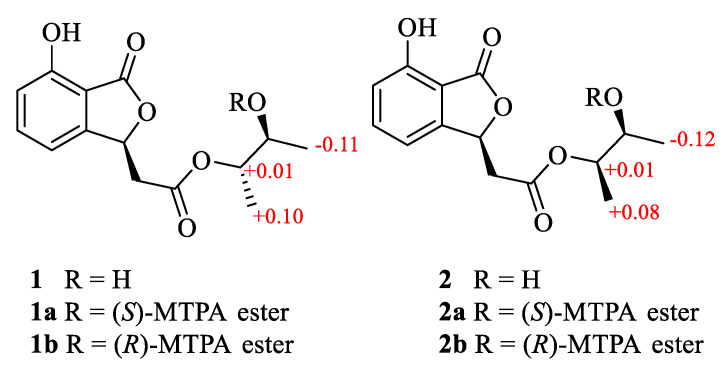
∆*δ* = *δ*_S_ − *δ*_R_ values in ppm obtained from the MTPA esters of **1** and **2**.

A 7-hydroxybenzofuran was assigned by ^1^H-^1^H COSY correlations (Figure S5) between H-4 and H-5, H-5 and H-6, H-6 and H-7, and HMBC correlations (Figure S6) from H-4 to C-3a and C-7a, and H-3 to ester carbonyl C-1, as well as four-bond W-type correlation from H-6 to ester carbonyl C-1 (Figure 2). The HMBC correlations from H-8 to C-3 and H-3 to C-9 indicated that C-8 of acetoxy was linked to C-3 of 7-hydroxy-benzofuran group. At the same time, ^1^H-^1^H COSY correlations between H-12 and H-10, H-10 and H-11, H-11, and HMBC correlations from a H-12 to C-10 and C-11, and H-13 to C-10 constructed a 2,3-butanediol group. The key HMBC correlations from H-10 to another ester carbonyl C-9 suggested that 2,3-butanediol group was connected to benzofuran moiety by ester bond. Finally, compound **1** was crystallized upon slow evaporation of chloroform solvent to give a crystal of the monoclinic space group *P*2_1_, which was detected by X-ray crystallography. The refinement of the Cu Kα data resulted in a Flack parameter [18] of 0.04(17) and a Hooft parameter [19] of 0.07(5), which assigned the absolute configuration of **1** as 3*S*, 10*S*, and 11*S* (Figure 3). The absolute configuration of secondary alcohol was further resolved by a modified Mosher’s method [20,21]. The chemical shifts for H-10, H-11, H-12, H-13 of **1a** and **1b** were measured as *δ*_H_ 5.19, 5.32, 1.20, 1.17 for **1a**, and *δ*_H_ 5.18, 5.31, 1.31, 1.07 for **1b**, respectively. The observed differences of chemical shifts (∆*δ* = *δ*_S_ − *δ*_R_) (Figure 4) indicated that the C-11 absolute configuration is *S* in agreement with the X-ray crystallography analysis. 

Alternabenzofuran B (**2**) was isolated as a yellow oil and had the same molecular formula (C_14_H_16_O_6_) as alternabenzofuran A (**1**) established by the HR-ESIMS ions at *m*/*z* 279.08783 [M−H]^−^ (calcd for C_14_H_15_O_6_, 279.08741) (Figure S11). Compound **2** shared the same planar structure as **1**, and was further identified by 2D NMR spectra (^1^H-^1^H COSY, HSQC, and HMBC) (Figure S14–S16). The minor chemical shift variation of C-10 (*δ*_C_ 76.3, *δ*_H_ 4.86 for **1**; *δ*_C_ 76.4, *δ*_H_ 4.85 for **2**), C-11 (*δ*_C_ 70.1, *δ*_H_ 3.73 for **1**; *δ*_C_ 70.0, *δ*_H_ 3.75 for **2**), C-12 (*δ*_C_ 19.1, *δ*_H_ 1.18 for **1**; *δ*_C_ 19.2, *δ*_H_ 1.19 for **2**) but obvious chemical shift variation of C-13 (*δ*_C_ 16.4, *δ*_H_ 1.25 for **1**; *δ*_C_ 16.3, *δ*_H_ 1.20 for **2**) were observed, it suggested that **2** should be a 10-epimer of **1**. At the same time, the absolute configuration of C-11 in **2**, bearing a secondary hydroxyl group (11*S*), was also identified as same as that of **1** according to the modified Mosher's method. Therefore, compound **2** was identified as 10-epimer of **1**, and named as alternabenzofuran B.

Alternaterpenoid A (**3**) was isolated as a white power and gave a molecular formula of C_15_H_22_O_2_ as determined from HR-ESIMS *m/z* 235.16949 [M + H]^+^ (calcd for C_15_H_23_O_2_, 235.16949), implying 4 degrees of unsaturation (Figure S21). The ^1^H NMR spectrum (Figure S22) (Table 2) showed one olefinic proton [*δ*_H_ 5.53 (1H, s)], one methine proton [*δ*_H_ 1.97 (1H, dd, *J* = 4.7, 9.1 Hz)], five methylene protons [*δ*_H_ 1.39 (1H, m),1.29(1H,m); 1.54(1H, td, *J* = 1.6, 3.3 Hz ), 1.32 (1H, td, *J* = 1.6, 3.4 Hz); 1.83 (1H, dt, *J* = 3.5, 13.6 Hz), 1.61 (1H, t, *J* = 3.6 Hz); 1.69 (1H, d, *J* = 4.1 Hz), 0.05 (1H, t, *J* = 4.3 Hz); 3.22 (1H, d, *J* = 11.3 Hz), 3.14 (1H, d, *J* = 11.3 Hz)] and three methyl protons [*δ*_H_ 2.09 (3H, s); 1.18(3H, s); 1.45(3H, s)]. The ^13^C NMR spectrum (Figure S23) displayed 15 carbons signals, including one conjugated carbonyl carbon (*δ*_C_ 202.5), two double bond carbons (*δ*_C_ 164.7 and 118.0), and 12 sp^3^ hybridized carbons.

**Figure 5:**
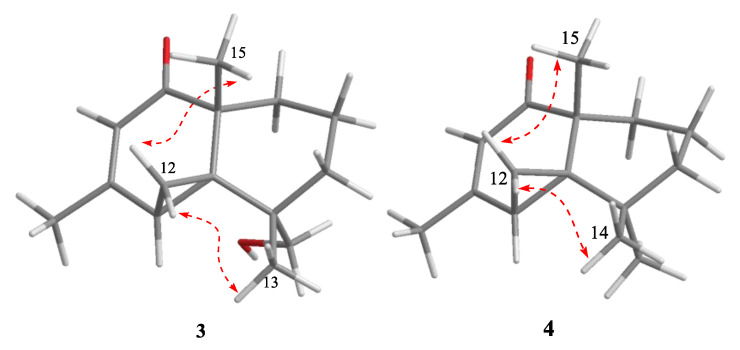
Key NOESY (dashed blue arrow) correlations of compounds **3** and **4**.

**Figure 6:**
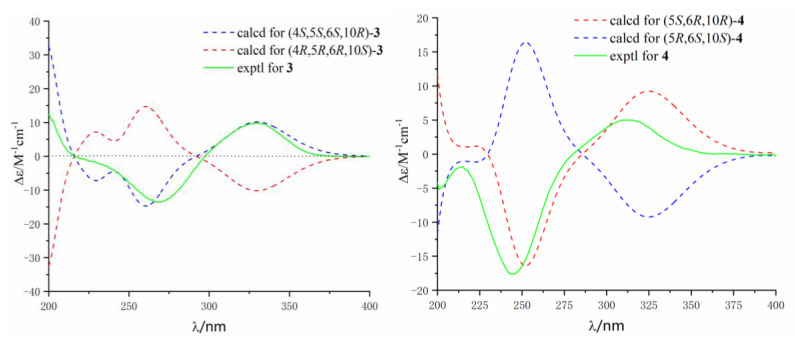
Experimental and calculated ECD spectra of compounds **3** and **4** (in MeOH).

The ^1^H–^1^H COSY correlations (Figure S25) between H-1 and H-2, H-2 and H-3, and the key HMBC correlations (Figure S26) from methyl protons H-15 to C-1, C-5, and C-10, and H-13 to C-3, C-4, C-5, and C-14, established a cyclohexane moiety (A ring) attached with two methyl and hydroxymethyl at C-10 and C-4, respectively. Another cyclohexanone moiety (B ring) was assigned by the key HMBC correlations from methyl H-15 to carbonyl C-9, and two quandary carbons C-5 and C-10, another methyl H-11 to C-6, C-7, and C-8, and methylene protons H-12 to C-5 and C-6. The remaining three-member ring (C ring) was identified by ^1^H-^1^H COSY correlations between H-6 and H-12, and key HMBC correlations from H-12 to C-4, C-5, C-6, C-7, and C-10. The planar structure with a 6/6/3 rings system was finally completed and belongs to the sesquiterpene thujopsene family [22]. The relative configuration of **3** was determined by the detailed analysis of NOESY data (Figure 5). NOE correlations of H-13 with H-12, and H-12 with H-15 suggested that the orientation of H-12, H-13, and H-15 were on the same side and the relative configuration of **3** were 4S*,5S*,6S*,10R*. The theoretical ECD spectra were calculated by a quantum chemical method at the [RB3LYP/6311+G(2d,p)] level, and the predicted ECD curve of (4*S*,5*S*,6*S*,10*R*)-**3** was in good agreement with that of the experimental one (Figure 6). Therefore, the structure of **3** was established as (4*S*,5*S*,6*S*,10*R*)-**3** and named alternaterpenoid A. 

**Figure 7:**
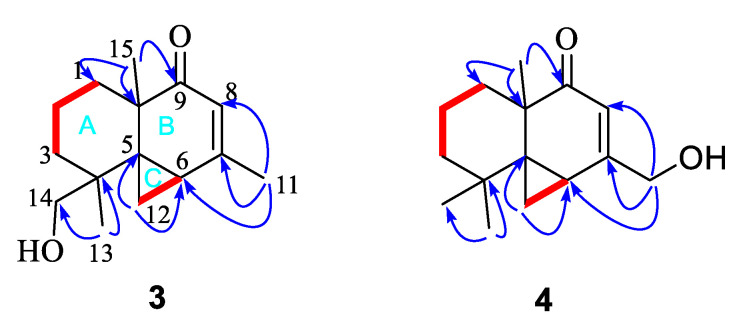
Key ^1^H-^1^H COSY (red line) and HMBC (blue arrow) correlations of compounds **3** and **4**.

Alternaterpenoid B (**4**) was obtained as white power. According to HR-ESIMS *m/z* 235.1691 [M + H]^+^ (calcd for C_15_H_23_O_2_, 235.1693) analysis (Figure S29), compound **4** was found to have a molecular formula (C_15_H_22_O_2_) as that of **3**. The 1D NMR data (Figures S30 and S31) of **4** were closely comparable to those of **3**, except for the change in the substitution group on C-11(71.3)/C-14(27.0), resulting in one hydroxymethyl on C-7(164.6) and C-4 (51.0) with methyl in **4**. The deduction was further confirmed by the HMBC correlations (Figure S34) from the hydroxymethyl protons H-11 to C-6, C-7, and C-8, see Figure 7. The relative configuration of **4** was identified to be identical to **3** by interpretation of its NOESY spectrum (Figure 5 and Figure S35). The theoretical ECD spectra were calculated by a quantum chemical method at the [RB3LYP/6311+G(2d,p)] level, and the predicted ECD curve of (5*S*,6*R*,10*R*)-**4** was in good agreement with that of the experimental one (Figure 6). Therefore, the structure of **4** was established as (5*S*,6*R*,10*R*)-**4**, and named alternaterpenoid B. 

Additionally, 18 known compounds were identified as isobenzofuranone A (**5**) [23], isoochracinic acid (**6**) [24], (*R*)-1,6-dihydroxy-8-methoxy-3a-methyl-3,3a-dihydrocyclopenta[c]isochromene-2,5-dione (**7**) [25], dihydroaltenuenes A (**8**) [25], phialophriol (**9**) [25,26], ± talaroflavone (**10**), alternariol-9-*O*-methyl ether (**11**) [27], alternariol (**12**) [27], 2-methyl-9-methoxy alternariol (**13**) [28], 3’-hydroxyalternariol 5-*O*-methyl ether (**14**) [29], alternariol-1’-hydroxy-9-methyl ether (**15**) [30], dehydroaltenusin (**16**) [31], alteryulactone (**17**) [31,32], tenuissimasatin (**18**) [33], 5’-methoxy-6-methyl-biphenyl-3,4,3’-triol (**19**) [29], altenusin (**20**) [34], 2,5-dimethyl-7-hydroxychromone (**21**) [35] and walterolactone C (**22**) [36] by comparing their spectroscopic data with published literature values.

In this work, all isolated compounds (**1**–**22**) were evaluated for their inhibition of nitric oxide (NO) production in RAW264.7 cells activated by lipopolysaccharide (LPS) using the Griess assay with indomethacin as a positive control (Table 3). Compounds **2**, **3**, **7**, **9**,**10**, **12**–**15**, **17**,**18** and **20**–**22** showed stronger anti-inflammatory activity compared to the positive control indomethacin whose IC_50_ was 35.8 ± 5.7 μM. Among them, compounds **3**, **7**, **9** and **14** displayed significant inhibitory effects on the production of NO with IC_50_ values below 10 μM, while compounds **5** and **11** exhibited moderate anti-inflammatory activity with IC_50_ values of 41.1 and 39.0 μM, respectively. To investigate whether the inhibitory activities of the active compounds were due to their cytotoxicity, the effects of the tested compounds on cell proliferation/viability were evaluated using the MTT method. Meanwhile, compounds **2**, **9**, **11**, **14**, and **17** (up to 100 μM) did not show any significant cytotoxicity with LPS treatment for 24 h. The pro-inflammatory enzymes, inducible nitric oxide synthase (iNOS) for nitric oxide (NO) production and cyclooxygenase-2 (COX-2) for prostaglandin production, have been shown to play key roles in inflammatory processes. Therefore, further studies are required to clarify the underlying mechanism of the active compounds.

In comparison of anti-inflammatory activity of two sesquiterpene thujopsene, **3** showed much stronger activity than that of **4**, indicating that the hydroxymethyl group (C-14) played an important role in anti-inflammatory action. For isocoumarins with 6/6/5 system (**8** and **9**), the keto carbonyl group at C-9 made no difference to the anti-inflammatory activity, while the hydroxyl group at C-9 made a more positive contribution to the anti-inflammatory activity. For another isocoumarins with 6/6/6 system (**11**–**15**), the substitution with the hydroxyl group at C-10 made a more positive contribution to the anti-inflammatory activity, while other substitution made no difference to the anti-inflammatory activity. 

There has been a tremendous increase in pharmacological research on anti-inflammatory of marine-derived molecules, and more than 150 anti-inflammatory compounds derived from marine fungi have been reported past two decades [37,38]. The anti-inflammatory compounds are classified into different chemical classes, such as terpenes [39,40], steroids [41], polyketides [15], alkaloids [42], and peptides [43]. For example, tanzawaic acid Q (isolated from a marine-derived fungus, *Penicillium steckii* 108YD142) inhibited the lipopolysaccharide (LPS)-induced inducible nitric oxide synthase (iNOS) and cyclooxygenase-2 (COX-2) proteins and mRNA expressions in RAW 264.7 macrophages. Amestolkolides B (obtained from the mangrove endophytic fungus *Talaromyces amestolkiae* YX1) showed strong anti-inflammatory activity by inhibiting nitric oxide (NO) production in lipopolysaccharide activated in RAW264.7 cells with IC_50_ values of 1.6 ± 0.1 *Μ*m in vitro [40]. A serial of mono- and dimeric sorbicillinoids (isolated from the marine-derived fungus *Trichoderma reesei* 4670) exhibited potent anti-inflammatory activity by inhibiting the production of NO in RAW264.7 cells activated by lipopolysaccharide with IC_50_ values in the range from 0.94 to 38 μM, whose structure–activity relationships were discussed [15]. Oxepinamide A (isolated from a marine-derived fungus *Acremonium* sp. from the surface of the Caribbean tunicate *Ecteinascidia turbinata*.) showed potent anti-inflammatory effect with the inhibition rate of 82% at the standard testing dose of 50 µg per ear by a topical resiniferatoxin (RTX)-induced mouse ear edema assay [42]. Alternaramide (isolated from a marine *Alternaria* sp. SF-5016) showed the inhibition of the production of PGE2 and NO correlated with down-regulation of iNOS and COX-2 expression in LPS-induced RAW264.7 and BV2 macroglia cells with IC_50_ values ranging from 27.63 to 40.52 µM, and suppressed the NF-κB and MAPK signaling pathway, as well as reduced the Toll-like receptor 4 (TLR4) and myeloid differentiation primary response gene 88 (MyD88) at the mRNA and protein levels [43]. Though there is no marine-derived anti-inflammatory agent currently on the market, the chemical diversity and biological activities of marine-derived molecules will provide medical and chemical researchers with a plenty variety of promising lead compounds for the development of anti-inflammatory marine drugs.

## 3. Materials and Methods

### 3.1. General Experimental Procedures

Optical rotations were measured on an MCP 200 polarimeter by using a Na lamp (Anton Paar). UV spectra were recorded using a Shimadzu UV-2501PC spectrometer (Shimadzu, Kyoto, Japan). To obtained ECD experiment data, Chirascan and Chirascan-Plus circular dichroism spectrometers (Applied Photophysics Ltd., Surrey, UK) were used. IR spectra were recorded using a Fourier transformation infra-red spectrometer coupled with infrared microscope EQUINOX 55 (Bruker, Rheinstetten, Germany). NMR spectra were obtained with a Bruker Avance 400 MHz spectrometer with tetramethylsilane as the internal standard (Bruker, Karlsruhe, Germany). HR-ESIMS data were determined by an LTQ-Orbitrap LC-MS spectrometer (Thermo Corporation, Waltham, MA, USA). ESIMS were acquired in an ACQUITY QDA (Waters Corporation, Milford, MA, USA). Silica gel 200–300 mesh (Qing dao Marine Chemical Factory, Qingdao, China) and Sephadex LH-20 (GF Healthcare, Littile Chalfont, UK) was used for column chromatography (CC). Semipreparative HPLC was performed on an Essentia LC-16 (Shimadzu, Shanghai, China). Thin layer chromatography was carried out on Pre-coated silica gel plates (Qingdao Huang Hai Chemical Group Co., G60, F-254, China).

### 3.2. Biological Material

The fungal strain 5102 was isolated from an actiniae collected in the Laishizhou island (22°27′49.7″ N 114°32′21.4″ E), Shenzhen City, Guangdong Province, China, in April 2016. The fungus was identified as *Alternaria* by an ITS sequence with 99% query coverage and 99% similarity to which has been deposited in GenBank under accession number EFJ809940.1. The fungal strain 5102 was deposited in GenBank with accession number MT742159.

### 3.3. Extraction, Isolation, and Characterization

The fungus was activated and purified on PDA plate, then implanted in a conical flask with PDB liquid (four 500 mL Erlenmeyer flasks; each containing 12 g of PDB powder and 15 g of artificial sea salt and 500 mL distilled H_2_O) and cultured in a shaker chamber for three days (150 RPM, 28 °C) to obtained seed liquid. The fungus’s seed liquid cultured on a rice medium (150 bottles 500 mL conical flask; each bottle with 50 g of rice, 15 g of sea salt and 60 mL sterile water) with room temperature under daylight and stilling culture for one month. The fermented material was extracted with EtOAc three times and concentrated under reduced pressure. The EtOAc extract (45.9 g) was subjected to CC on silica gel (100–200 mesh) and was eluted with PE (petroleum ether)/EtOAc of increasing polarity (from 80:20 to 0:100) to afford seven fractions (A−F). 

Fr. A was fractionated on a CC on silica gel (200-300 mesh) eluting with PE/EA (from 75:15 to 60:40) to afford 2 fractions (Fr.A.1, Fr.A.2). Fr.A.1 was further separated on Sephadex LH-20 (CH_2_Cl_2_/MeOH v/v, 1:1) and purified by NR-HPLC (n-hexane:IPA (isopropyl alcohol) v/v, 97:3, flow rate 1.5 mL/min, Ultimate S column 10 × 250 mm, 5 μm) to give **3** (10.0 mg). Fr.A.2 was separated on Sephadex LH-20 (CH_2_Cl_2_/MeOH v/v, 1:1) and further applied to a silica gel column eluting with PE/EA (from 75:15 to 60:40) to afford **8** (19.6 mg), **9** (18.0 mg) and **11** (46.0 mg), and others was purified by RP-HPLC with n-hexane/IPA (from 95:5 to 90:10) to give **7** (27 mg), **15** (17.3 mg), **17** (7.8 mg), **21** (9.4 mg) and **4** (3.0 mg). Fr. B was subjected to Sephadex LH-20 (CH_2_Cl_2_/MeOH v/v, 1:1) to afford 6 fractions (Fr.B.1 to Fr.B.6). Fr.B.1 was fractionated on a CC on silica gel (200-300 mesh) eluting with PE/EA (from 80:20 to 70:30) to afford 3 fractions (Fr.B.1.1, Fr.B.1.2 and Fr.B.1.3). Fr.B.1.1 was further applied to a Sephadex LH-20 (CH_2_Cl_2_/MeOH v/v, 1:1) to afford **22** (15.3 mg). Fr.B.1.2 was purified on silica gel column (PE/EA, 75:25) to obtain **5** (16.4 mg) and **18** (10.3 mg). Fr.B.1.3 was separated on a CC on silica gel eluting with CH_2_Cl_2_/MeOH (from 97.5:2.5 to 96:4) to give **12** (6.4 mg), **13** (168 mg), **14** (23.5 mg), **16** (5 mg), **19** (4 mg) and **20** (3.3 mg). Fr.B.2 was also purified on silica gel column with PE/EA (from 80:20 to 70:30) to obtain two fractions (Fr.B.2.1 and Fr.B.2.2). Fr.B.2.1 was successively applied to a silica gel with CH_2_Cl_2_/MeOH (97:3) to give **6** (4.4 mg), 2-8a/2-8b (15.3 mg). Fr.B.2.2 was finally purified by NR-HPLC (n-hexane:IPA v/v, 75:25) to afford **2** (14.3 mg) and **1** (18.2 mg). 

#### 3.3.1. Alternabenzofuran A (**1**)

Colorless cluster crystals; ${\left[\alpha \right]}_{D}^{25}$ −2.5 (*c* 0.1, MeOH); UV (MeOH) *λ_max_* (log ε) 300 (2.52), 235 (2.79), 206 (3.51) nm; IR (neat) *v_max_*: 3444, 2968, 2923, 2857, 1743, 1609, 1471, 1384, 1317, 1286, 1265, 1181, 1087, 1009, 917, 865, 798, 690 cm^−1^ (Figure S8); ^1^H NMR (CDCl_3_, 400 MHz) and ^13^C NMR (CDCl_3_, 100 MHz) data, see Table 1; HR-ESIMS *m/z* 279.08790 [M-H]^−^ (calcd for C_14_H_15_O_6_, 279.08741). 

#### 3.3.2. Alternabenzofuran B (2)

Yellow oily; ${\left[\alpha \right]}_{D}^{25}$ +3.3 (*c* 0.1, MeOH); UV (MeOH) *λ_max_* (log ε) 300 (2.68), 237 (2.87), 206 (3.64) nm; IR (neat) *v_max_*: 3439, 2982, 2928, 1747, 1609, 1477, 1382, 1314–1280, 1191, 1078, 1005, 931, 863, 803, 690 cm^−1^ (Figure S18); ^1^H NMR (CDCl_3_, 400 MHz) and ^13^C NMR (CDCl_3_, 100 MHz) data, see Table 1; HR-ESIMS *m/z* 279.08783 [M-H]^−^ (calcd for C_14_H_15_O_6_, 279.08741).

#### 3.3.3. Alternaterpenoid A (3) 

White power; ${\left[\alpha \right]}_{D}^{25}$ +1.7 (*c* 0.1, MeOH); UV (MeOH) *λ_max_* (log ε) 266 (3.13), 230 (2.72) nm; CD (MeOH) *λ_max_* (Δε) 267 (−13.5), 330 (10.1) nm; IR (neat) *v_max_*: 3430, 2928, 2864, 1761, 1723, 1644, 1438, 1368, 1260, 1079, 1034, 872, 798 cm^−1^ (Figure S28); ^1^H NMR (CDCl_3_, 400 MHz) and ^13^C NMR (CDCl_3_, 100 MHz) data, see Table 2; HR-ESIMS *m/z* 235.16949 [M + H]^+^ (calcd for C_15_H_23_O_2_, 235.16949).

#### 3.3.4. Alternaterpenoid B (4)

White power; ${\left[\alpha \right]}_{D}^{25}$ −0.0 (*c* 0.1, MeOH); UV (MeOH) *λ_max_* (log ε) 244 (2.67), 227 (2.55) nm; CD (MeOH) *λ_max_* (Δε) 244 (−17.6), 312 (5.0); IR (neat) *v_max_*: 3440, 2923, 2864, 1674, 1457, 1373, 1243, 1177, 1094, 1049, 968, 848 cm^−1^ (Figure S36); ^1^H NMR (CDCl_3_, 400 MHz) and ^13^C NMR (CDCl_3_, 100 MHz) data, see Table 2; HR-ESIMS *m/z* 235.1691 [M + H]^+^ (calcd for C_15_H_23_O_2_, 235.1693).

### 3.4. X-ray Crystallographic Analysis of Compound 1

Colorless crystals of compound **1** were obtained from a solvent of chloroform. Crystal data were acquired using the hemisphere technique on a Rigaku Oxford Diffraction diffractometer with graphite-monochromated Cu-Kα (radiation λ = 1.54178 Å). The structure was solved by direct methods using SHELXS-97; refinement was done by full-matrix least-squares on F2 using the SHELXL-97 program suite on Olex2 Launcher.

Crystal data of (**1**): C_14_H_15_O_6_, Mr = 280.27, monoclinic, *a* = 9.69498(8) Å, *b* = 4.96003(5) Å, *c* = 13.68007(10) Å, *α* = 90°, *β* = 93.4053(7)°, *γ* = 90°, *V* = 656.677(10) Å^3^, space group *P*_21_, *T* = 150.0(4) K, *Z* = 2, *D*_calcd_ = 1.417 g/cm^3^, *μ* = 0.942 mm^−1^, and *F*(000) = 296.0. Crystal dimensions: 0.41 × 0.14 × 0.11 mm^3^. Independent reflections 2611 (*R*_int_ = 0.0443), The goodness of fit on F^2^ was 1.075. The final *R*_1_ values were 0.0331, *wR*_2_ = 0.0910 [*I* > 2*σ*(*I*)]. The Flack parameter was 0.04(17) and the Hooft parameter was 0.07(5). CCDC number: 2010422.

### 3.5. Preparation of (S)-MTPA Ester and (R)-MTPA Ester

#### 3.5.1. (*S*)-MTPA Ester (**1a**) and (*R*)-MTPA Ester (**1b**)

Compound **1** (1.0 mg, 10 μmol), (*R*)-MPTACl (10.0 μL, 50 μmol), and pyridine-*d*_5_ (0.5 mL) were mixed in an NMR tube to a reaction (room temperature, 24 h). Then the ^1^H NMR data of the (*S*)-MTPA ester derivative (**1a**) was measured directly on the reaction mixture. ^1^H NMR (Figure S9) (selected signals, pyridine-*d*_5_, 400 MHz) *δ*_H_: 5.19 (1H, m, H-10), 5.32 (1H, m, H-11), 1.20 (3H, d, H-12), 1.17 (3H, d, H-13).

Similarly, another reaction of **1** (1.0 mg, 4 μmol), (*S*)-MPTACl (10.0 μL, 52 *μ*mol), and pyridine-*d*_5_ (0.5 mL) was performed as described above for **1a** to afford **1b**. ^1^H NMR (Figure S10) (selected signals, pyridine-*d*_5_, 400 MHz) *δ*_H_: 5.18 (1H, m, H-10), 5.31 (1H, m, H-11), 1.31 (3H, d, H-12), 1.07 (3H, d, H-13).

#### 3.5.2. (*S*)-MTPA Ester (**2a**) and (*R*)-MTPA Ester (**2b**)

(*S*)-MTPA Ester (**2a**) and (*R*)-MTPA ester (**2b**) were easy to obtain refer to above method. ^1^H NMR (Figures S19 and S20) (selected signals, pyridine-*d*_5_, 400 MHz) **2a** *δ*_H_: 5.19 (1H, m, H-10), 5.35 (1H, m, H-11), 1.17 (3H, d, H-12), 1.17 (3H, d, H-13). **2b** *δ*_H_: 5.18 (1H, m, H-10), 5.33 (1H, m, H-11), 1.29 (3H, d, H-12), 1.09 (3H, d, H-13).

### 3.6. Calculation of the ECD Spectra

Molecular Merck force field (MMFF) and DFT/TD-DFT calculations were carried out with Spartan’ 14 software (Wavefunction Inc., Irvine, CA, USA) and Gaussian 09 program, respectively. Conformers within 10 kcal/mol energy window were obtained and optimized by DFT calculations at B3LYP/6-31G(d) level. Conformers with Bolzmann distribution over 1% were selected for ECD calculations in methanol at B3LYP/6-311+g(2d,p) level (Figures S67 and S68). The IEF-PCM solvent model for MeOH was used. ECD spectra were generated using the program SpecDis 3.0 (University of Würzburg, Würzburg, Germany) and OriginPro 8.5 (OriginLab, Ltd., Northampton, MA, USA) from dipole-length rotational strengths by applying Gaussian band shapes with sigma = 0.30 ev. All calculations were performed by Tianhe-2 in the National Super Computer Center in Guangzhou.

### 3.7. Cell Viability Assay and Anti-Inflammatory Activity

Cell viability was measured using the conventional MTT assay. RAW 264.7 cells were seeded in 96-well plates at a density of 1.5 × 10^5^ cells/mL. After 12 h, the cells were treated with LPS (1 µg/mL) and samples, followed by additional incubation for 24 h at 37 °C. MTT stock solution (2 mg/mL) was added to wells for a total reaction volume of 110 µL. After 4 h incubation, the supernatants were aspirated. The formazan crystals in each well were dissolved in 50 µL of DMSO, and the absorbance was measured using a microplate reader (Multiskan GO, Thermo Scientific, Waltham, MA, USA) at the wavelength of 490 nm. Relative cell viability was evaluated based on the quantity of MTT converted to the insoluble formazan salt. The optical density of formazan generated in the control cells represented 100% viability. The data were expressed as mean percentages of the viable cells compared to the respective control.

After pre-incubation of RAW 264.7 cells (1.5 × 10^5^ cells/mL) with LPS (1 µg/mL) and samples at 37 °C for 24 h, the quantity of nitrite accumulated in the culture medium was measured as an indicator of NO production. Briefly, 50 µL of cell culture medium were mixed with 100 µL Griess reagent, and incubated at room temperature for 10 min. The absorbance was determined at 540 nm wavelength with a microplate reader (Multiskan GO, Thermo Scientific, Waltham, MA, USA).

### 3.8. Statistical Analysis

Each experiment was performed at least three times independently, and the resulting data are presented as the mean ± standard deviation. The comparison of three or more groups used one-way analysis of variance, followed by Tukey’s multiple comparison tests. Statistical analysis was performed using GraphPad Prism software, version 3.03 (GraphPad Software Inc, GraphPad Software Inc., San Diego, CA, USA).

## 4. Conclusions

The fungal genus *Alternaria* are a significant source of pharmacologically active metabolites with interesting structural properties. Chemical investigation of an extract of marine-derived fungus *Alternaria* sp. 5102 from rice medium led to the discovery of two new benzofurans, alternabenzofurans A and B (**1** and **2**) and two new sesquiterpenoids, alternaterpenoids A and B (**3** and **4**), along with 18 known polyketides (**5**–**22**). Their structures were clearly elucidated by extensive spectroscopic analyses and X-ray crystallography, as well as the modified Mosher’s method. Most of isolated molecules (**2**, **3**, **5**, **7**, **9**–**18**, and **20**–**22**) exhibited potent anti-inflammatory activity by inhibiting the production of NO in RAW264.7 cells activated by lipopolysaccharide with IC_50_ values in the range from 1.3 to 41.1 μM. 

## Figures and Tables

**Table 1:** ^1^H (400MHz) and ^13^C (100 MHz) NMR data of **1** and **2** in CDCl_3_.

No.	1		2	
	*δ*_C_, Type	*δ*_H_, (*J* in Hz)	*δ*_C_, Type	*δ*_H_, (*J* in Hz)
1	171.5, C		171.6, C	
3	78.3, CH	5.90, t (6.6)	78.3, CH	5,87, t (6.5)
3a	149.0, C		149.0, C	
4	116.2, CH	6.96, dd (2.5, 7.3)	116.2, CH	6.96, dd (4.6, 7.0)
5	137.4, CH	7.56, t (7.9)	137.4, CH	7.56, t (7.8)
6	113.4, CH	6.96, dd (2.5, 7.3)	113.5, CH	6.96, dd (4.6, 7.0)
7	156.7, C		156.8, C	
7a	111.1, C		111.1, C	
8	39.7, CH_2_	2.95, m	39.7, CH_2_	2.94, m
9	168.8, C		168.9, C	
10	76.3, CH	4.86, m	76.2, CH	4.85, m
11	70.1, CH	3.73, m	70.0, CH	3.75, m
12	19.1, CH_3_	1.18, d (6.4)	19.2, CH_3_	1.19, d (6.3)
13	16.4, CH_3_	1.25, d (6.4)	16.3, CH_3_	1.20, d (6.3)

**Table 2:** ^1^H (400MHz) and ^13^C (100 MHz) NMR data of **1** and **2** in CDCl_3_.

No.	3		4	
	*δ*_C_, Type	*δ*_H_, (*J* in Hz)	*δ*_C_, Type	*δ*_H_, (*J* in Hz)
1	34.1, CH_2_	1.39, m 1.29, m	34.2, CH_2_	1.77, m 1.45, m
2	37.3, CH_2_	1.54, td (1.6, 3.3) 1.32, td (1.6, 3.4)	40.0, CH_2_	1.81, m 1.57, m
3	17.6, CH_2_	1.83, dt (3.5, 13.6) 1.61, t (3.6)	17.6, CH_2_	1.60, m 1.79, m
4	34.0, C		51.0, C	
5	36.7, C		42.9, C	
6	25.0, CH	1.97, dd (4.7, 9.1)	39.3, CH	1.37, m
7	164.7, C		164.6, C	
8	118.0, CH	5.53, s	123.1, CH	6.00, s
9	202.5, C		209.9, C	
10	46.8, C		41.4, C	
11	24.6, CH_3_	2.09, s	71.3, CH_2_	3.73, d (11.4) 3.32, d (11.4)
12	27.5, CH_2_	1.69, d (4.1) 0.05, t (4.3)	33.1, CH_2_	1.94, m 1.84, m
13	22.6, CH_3_	1.18, s	30.4, CH_3_	1.16, s
14	72.1, CH_2_	3.22, d (11.3) 3.14, d (11.3)	27.0, CH_3_	1.15, s
15	21.9, CH_3_	1.45, s	27.4, CH_3_	1.34, s

**Table 3:** Inhibitory activity of all compounds **1**–**22** against lipopolysaccharide (LPS)-induced NO production in the murine macrophage cell line (RAW 264.7 cells).

Compounds	IC_50_ (μM)	CC_50_ (μM) ^a^	SI ^b^
1	>50	>100	
18.7 ± 2.35	>100	
2.4 ± 0.79	24.9 ± 1.2	10.4
4	≈50	>100	
41.1 ± 4.78	>100	
5.2 ± 1.96	20.6 ± 2.3	4.0
8	>50	>100	
9	1.3 ± 0.10	>100	
10	23.9 ± 3.30	27.2 ± 1.6	1.1
11	39.0 ± 1.92	>100	
12	16.6 ± 1.60	50.4 ± 2.1	3.0
13	24.5 ± 4.51	48.2 ± 2.6	2.0
14	5.9 ± 0.48	>100	
15	26.3 ± 3.99	48.7 ± 1.8	1.9
17	16.2 ± 2.62	>100	
18	24.5 ± 1.06	28.3 ± 2.5	1.2
25.4 ± 3.03	>100	
14.9 ± 1.92	17.3 ± 2.2	1.2
22	14.9 ± 1.92	46.4 ± 1.7	3.1
Indometacin **^c^**	35.8 ± 5.7		

^a^ Values are taken as the means ± standard deviation, *n* = 3; ^b^ SI, selectivity index, calculated by CC_50_ /IC_50_; ^c^ Positive control.

## Supplementary Materials

The following are available online at https://www.mdpi.com/1660-3397/18/8/426/s1, HR-ESIMS, NMR of the new compounds as well as other supporting data.
