# Supplementary material for: Secondary Metabolites with Nitric Oxide Inhibition from Marine-Derived Fungus Alternaria sp. 5102
Source: Mar Drugs. 2020 Aug 14;18(8):426. doi: 10.3390/md18080426 (PMC7460390; doi:10.3390/md18080426)

Supporting Information

Secondary Metabolites with Nitric Oxide Inhibition from Marine-Derived Fungus *Alternaria* sp. 5102

Senhua Chen [2, 3,†], Yanlian Deng [1,†], Chong Yan [1], Zhenger Wu [2], Heng Guo [2], Lan Liu [2,3] and Hongju Liu [1,*]

[1] School of Pharmacy, Guangdong Medical University, Dongguan 523808, China; dengylian2016@163.com (Y.D.); jdsbj2000@163.com (C.Y.); liuhj8@mail2.sysu.edu.cn (H.L.)

[2] School of Marine Sciences, Sun Yat-sen University, Guangzhou 510006, China; chensenh@mail.sysu.edu.cn (S.C.); wuzher@mail2.sysu.edu.cn (Z.W.); hengeguo163@163.com (H.G.); cesllan@mail.sysu.edu.cn (L.L.);

[3] Southern Laboratory of Ocean Science and Engineering (Guangdong, Zhuhai), Zhuhai 519000, China.

* Correspondence: liuhj8@mail2.sysu.edu.cn; Tel.: *+86769-22896599*

‡ These authors contributed equally to this work.

Pages		
4	**Figure S1**	The HRESI-MS spectrum of compound **1**.
4	**Figure S2**	The ^{1}H NMR (400MHz) spectrum of compound **1** in $CDCl_3$
5	**Figure S3**	The ^{13}C NMR (100MHz) spectrum of compound **1** in $CDCl_3$
5	**Figure S4**	The HSQC spectrum of compound **1** in $CDCl_3$
6	**Figure S5**	The ^{1}H-^{1}H COSY spectrum of compound **1** in $CDCl_3$
6	**Figure S6**	The HMBC spectrum of compound **1** in $CDCl_3$
7	**Figure S7**	The NOESY spectrum of compound **1** in $CDCl_3$
7	**Figure S8**	The IR spectrum of compound **1**.
8	**Figure S9**	^{1}H (400 MHz) NMR spectrum of **1a** in pyridine-d_5
8	**Figure S10**	^{1}H (400 MHz) NMR spectrum of **1b** in pyridine-d_5
9	**Figure S11**	The HRESI-MS spectrum of compound **2**.
9	**Figure S12**	The ^{1}H NMR (400MHz) spectrum of compound **2** in $CDCl_3$-*d*.
10	**Figure S13**	The ^{13}C NMR (100MHz) spectrum of compound **2** in $CDCl_3$-*d*.
10	**Figure S14**	The HSQC spectrum of compound **2** in $CDCl_3$-*d*.
11	**Figure S15**	The ^{1}H-^{1}H COSY spectrum of compound **2** in $CDCl_3$
11	**Figure S16**	The HMBC spectrum of compound **2** in $CDCl_3$
12	**Figure S17**	The NOESY spectrum of compound **2** in $CDCl_3$
12	**Figure S18**	The IR spectrum of compound **2**.
13	**Figure S19**	^{1}H (400 MHz) NMR spectrum of **2a** in pyridine-d_5
13	**Figure S20**	^{1}H (400 MHz) NMR spectrum of **2b** in pyridine-d_5
14	**Figure S21**	The HRESI-MS/MS spectrum of compound **3**.
14	**Figure S22**	The ^{1}H NMR (400MHz) spectrum of compound **3** in $CDCl_3$
15	**Figure S23**	The ^{13}C NMR (100MHz) spectrum of compound **3** in $CDCl_3$
15	**Figure S24**	The HSQC spectrum of compound **3** in $CDCl_3$
16	**Figure S25**	The ^{1}H-^{1}H COSY spectrum of compound **3** in $CDCl_3$
16	**Figure S26**	The HMBC spectrum of compound **3** in $CDCl_3$
17	**Figure S27**	The NOESY spectrum of compound **3** in $CDCl_3$
17	**Figure S28**	The IR spectrum of compound **3**.
18	**Figure S29**	The HRESI-MS/MS spectrum of compound **4**.
18	**Figure S30**	The ^{1}H NMR (400MHz) spectrum of compound **4** in $CDCl_3$
19	**Figure S31**	The ^{13}C NMR (100MHz) spectrum of compound **4** in $CDCl_3$
19	**Figure S32**	The HSQC spectrum of compound **4** in $CDCl_3$
20	**Figure S33**	The ^{1}H-^{1}H COSY spectrum of compound **4** in $CDCl_3$
20	**Figure S34**	The HMBC spectrum of compound **4** in $CDCl_3$
21	**Figure S35**	The NOESY spectrum of compound **4** in $CDCl_3$
21	**Figure S36**	The IR spectrum of compound **4**.
22	**Figure S37**	The ^{1}H NMR (400MHz) spectrum of compound **5** in acetone-d_6.
22	**Figure S38**	The ^{13}C NMR (100MHz) spectrum of compound **5** in acetone-d_6.
23	**Figure S39**	The ^{1}H NMR (400MHz) spectrum of compound **6** in acetone-d_6.
23	**Figure S40**	The ^{13}C NMR (100MHz) spectrum of compound **6** in acetone-d_6.
24	**Figure S41**	The ^{1}H NMR (400MHz) spectrum of compound **7** in acetone-d_6.
24	**Figure S42**	The ^{13}C NMR (100MHz) spectrum of compound **7** in acetone-d_6.
25	**Figure S43**	The ^{1}H NMR (400MHz) spectrum of compound **9** in $CDCl_3$-*d*.

25	**Figure S44**	The ^{13}C NMR (100MHz) spectrum of compound **9** in $CDCl_3$-*d*.
26	**Figure S45**	The ^{1}H NMR (400MHz) spectrum of compound **10** in DMSO-d_6.
26	**Figure S46**	The ^{13}C NMR (100MHz) spectrum of compound **10** in DMSO-d_6.
27	**Figure S47**	The ^{1}H NMR (400MHz) spectrum of compound **11** in acetone-d_6.
27	**Figure S48**	The ^{13}C NMR (100MHz) spectrum of compound **11** in acetone-d_6.
28	**Figure S49**	The ^{1}H NMR (400MHz) spectrum of compound **13** in MeOH-d_4.
28	**Figure S50**	The ^{13}C NMR (100MHz) spectrum of compound **13** in MeOH-d_4.
29	**Figure S51**	The ^{1}H NMR (400MHz) spectrum of compound **14** in DMSO-d_6.
29	**Figure S52**	The ^{13}C NMR (100MHz) spectrum of compound **14** in DMSO-d_6.
30	**Figure S53**	The ^{1}H NMR (400MHz) spectrum of compound **16** in acetone-d_6.
30	**Figure S54**	The ^{13}C NMR (100MHz) spectrum of compound **16** in acetone-d_6.
31	**Figure S55**	The ^{1}H NMR (400MHz) spectrum of compound **17** in acetone-d_6.
31	**Figure S56**	The ^{13}C NMR (100MHz) spectrum of compound **17** in acetone-d_6.
32	**Figure S57**	The ^{1}H NMR (400MHz) spectrum of compound **18** in $CDCl_3$-*d*.
32	**Figure S58**	The ^{13}C NMR (100MHz) spectrum of compound **18** in $CDCl_3$-*d*.
33	**Figure S59**	The ^{1}H NMR (400MHz) spectrum of compound **19** in acetone-d_6.
33	**Figure S60**	The ^{13}C NMR (100MHz) spectrum of compound **19** in acetone-d_6.
34	**Figure S61**	The ^{1}H NMR (400MHz) spectrum of compound **20** in acetone-d_6.
34	**Figure S62**	The ^{13}C NMR (100MHz) spectrum of compound **20** in acetone-d_6.
35	**Figure S63**	The ^{1}H NMR (400MHz) spectrum of compound **21** in DMSO-d_6.
35	**Figure S64**	The ^{13}C NMR (100MHz) spectrum of compound **21** in DMSO-d_6.
36	**Figure S65**	The ^{1}H NMR (400MHz) spectrum of compound **22** in $CDCl_3$-*d*.
36	**Figure S66**	The ^{13}C NMR (100MHz) spectrum of compound **22** in $CDCl_3$-*d*.
37	**Experimental Section**	
37	**Table S1**	Energy Analysis for the Conformers of **(**4*S*,5*S*,6*S*,10*R***)-3.**
38	**Figure S67**	B3LYP/6-31G(d) optimized low-energy conformers of **(**4*S*,5*S*,6*S*,10*R***)-3**.
38	**Table S2**	Energy Analysis for the Conformers of **(**5*S*,6*R*,10*R***)-4.**
39	**Figure S68**	B3LYP/6-31G(d) optimized low-energy conformers of **(**5*S*,6*R*,10*R***)-4**.
39-40		NMR data of known compounds
41-43		Statistical Analysis

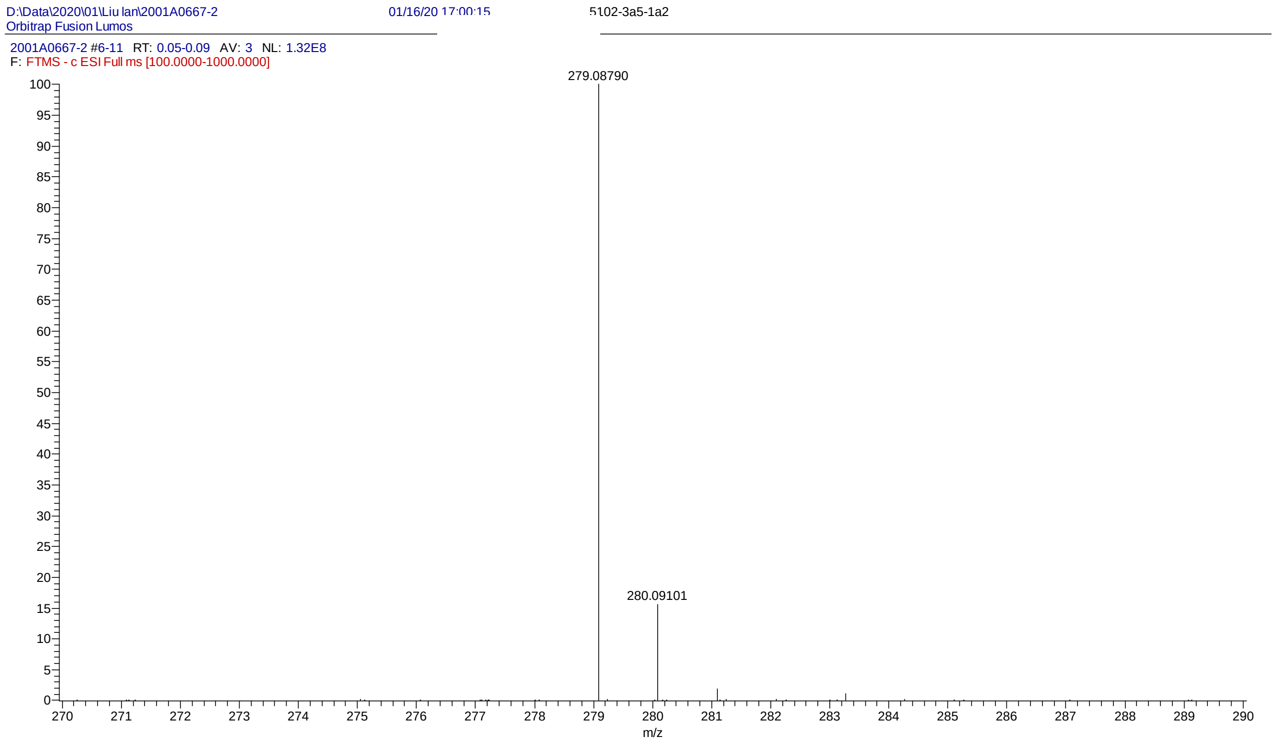

Figure S1. The HRESIMS spectrum of compound **1**.

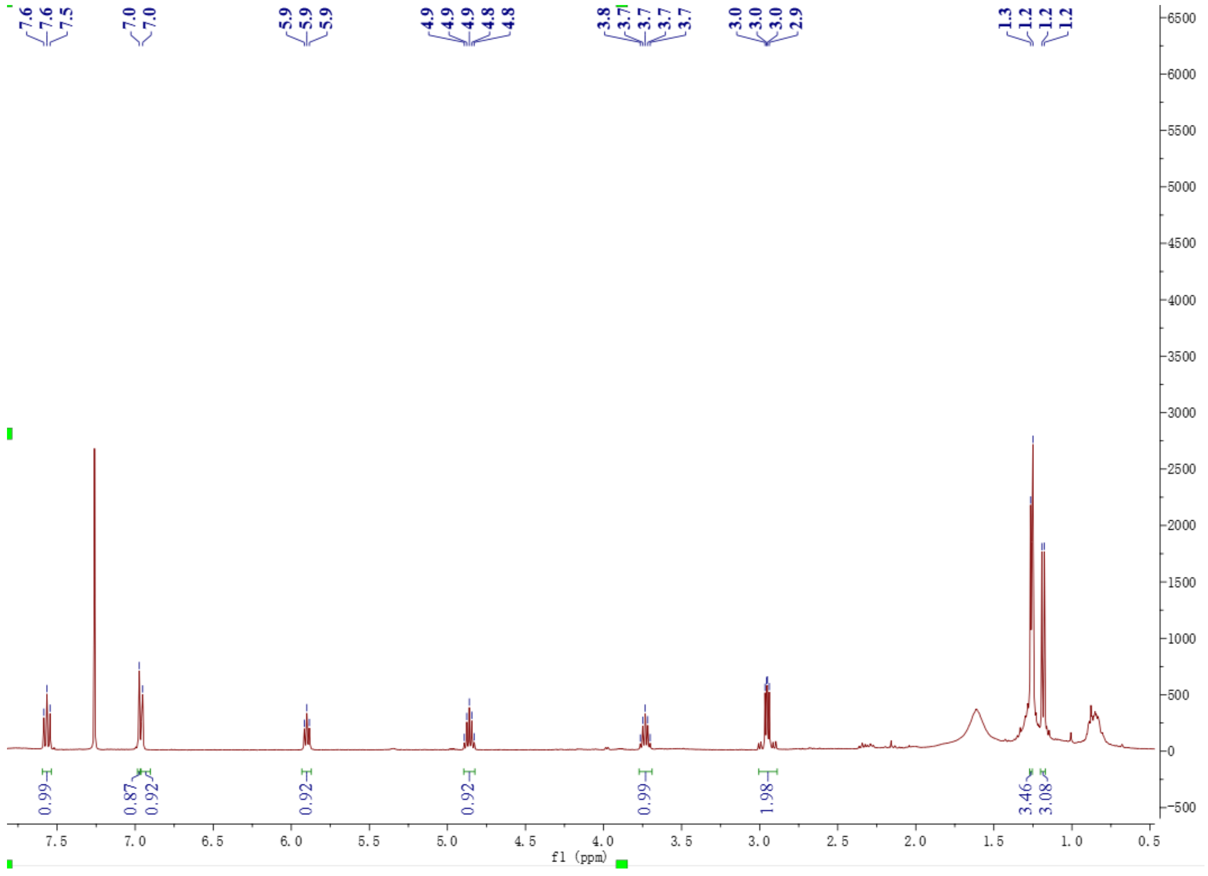

Figure S2. The ^{1}H NMR (400MHz) spectrum of compound **1** in $CDCl_3$.

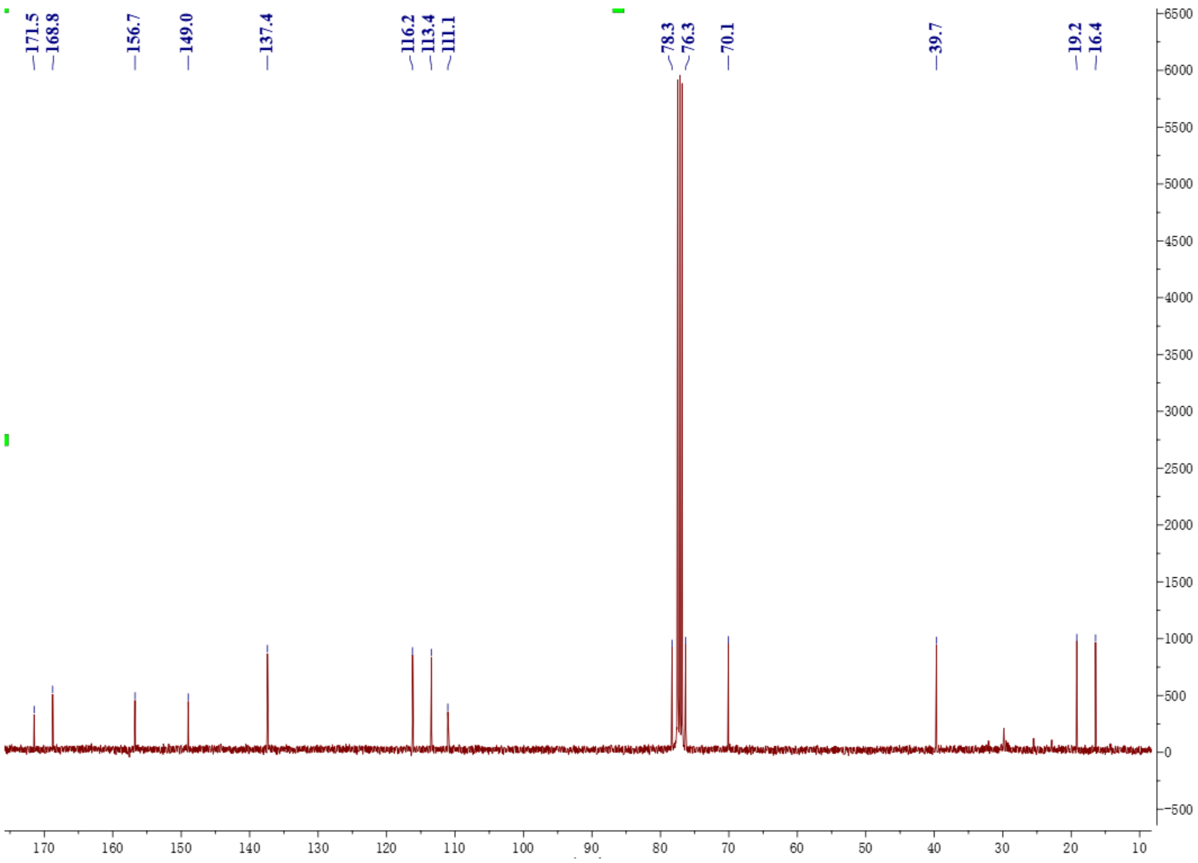

Figure S3. The ^{13}C NMR (100MHz) spectrum of compound **1** in $CDCl_3$.

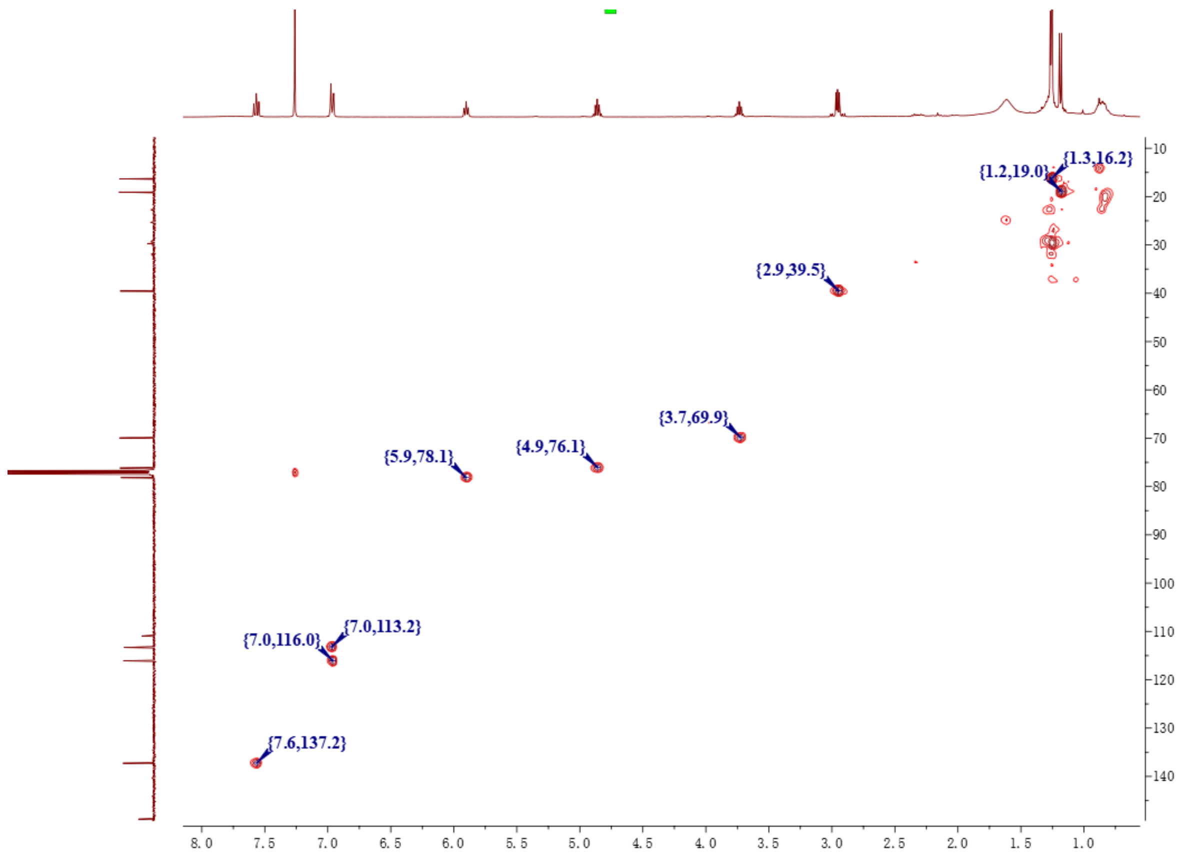

Figure S4. The HSQC spectrum of compound **1** in $CDCl_3$.

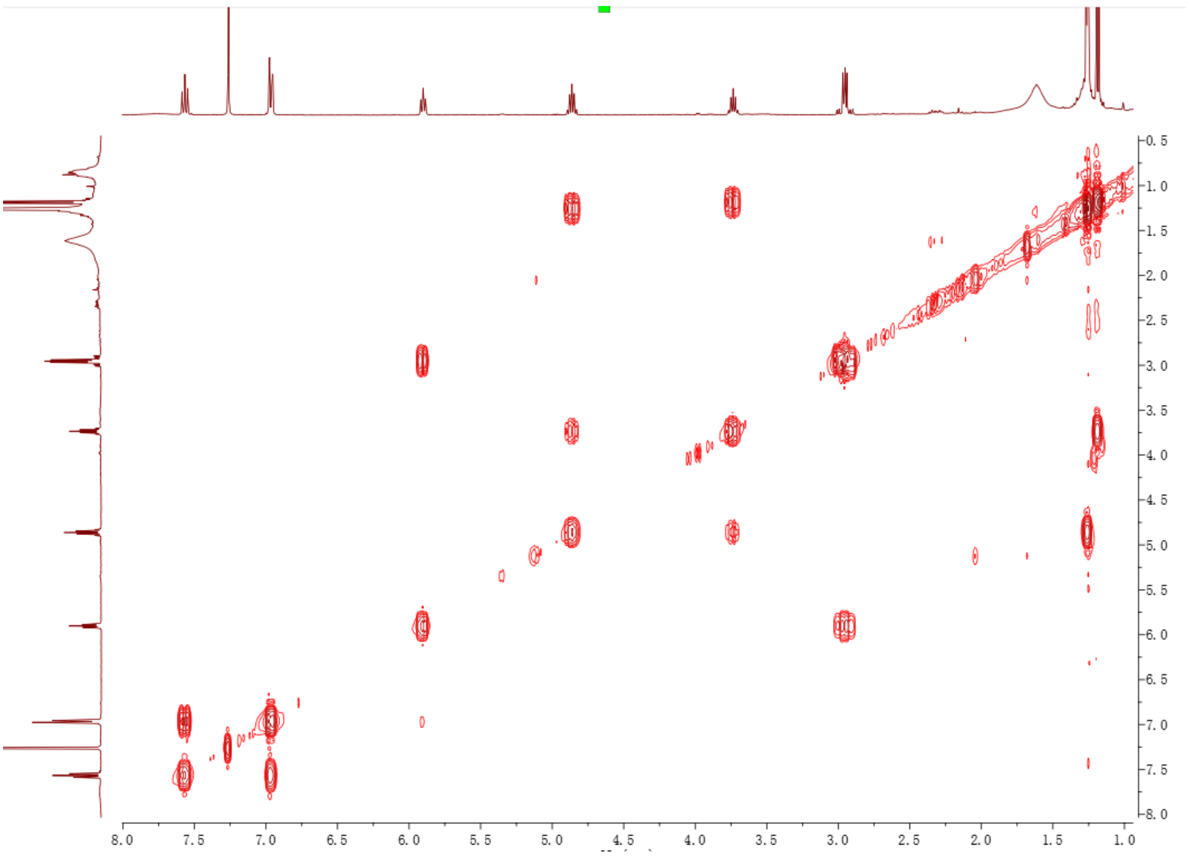

Figure S5. The ^{1}H-^{1}H COSY spectrum of compound **1** in $CDCl_3$.

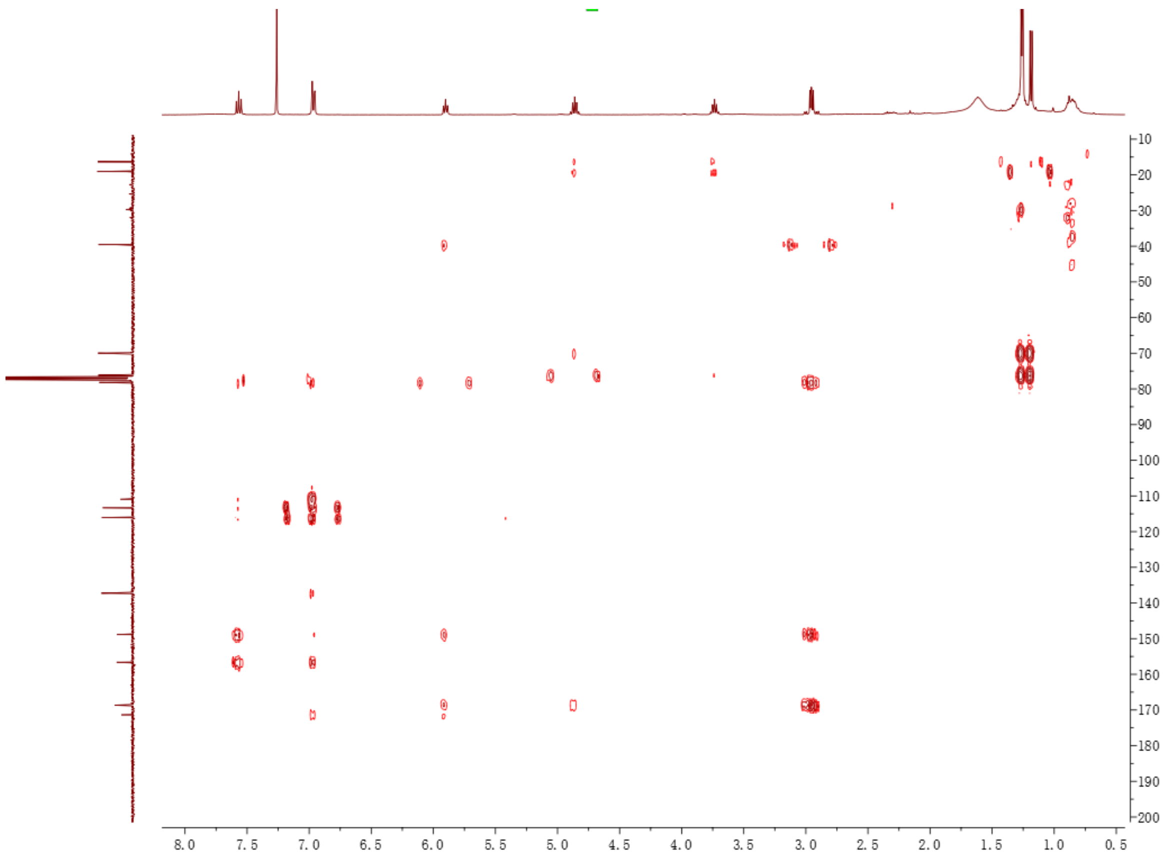

Figure S6. The HMBC spectrum of compound **1** in $CDCl_3$.

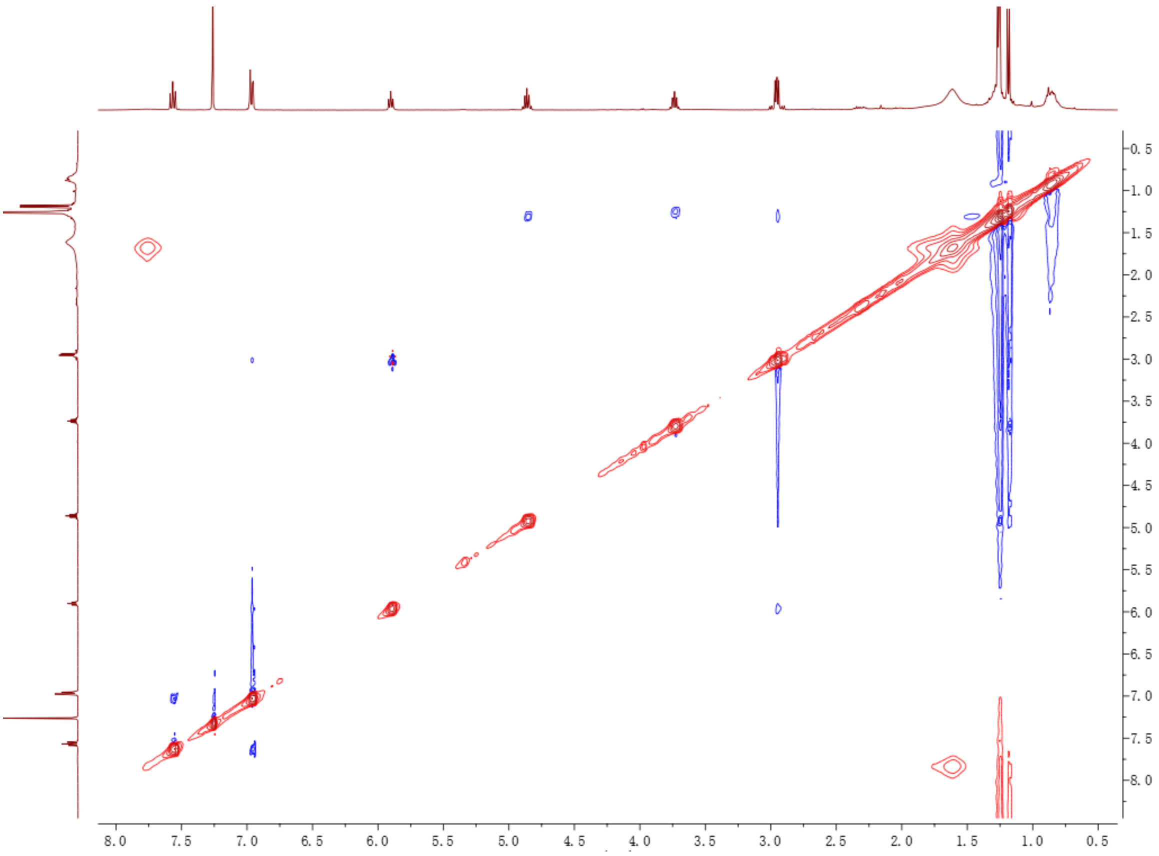

Figure S7. The NOESY spectrum of compound **1** in $CDCl_3$.

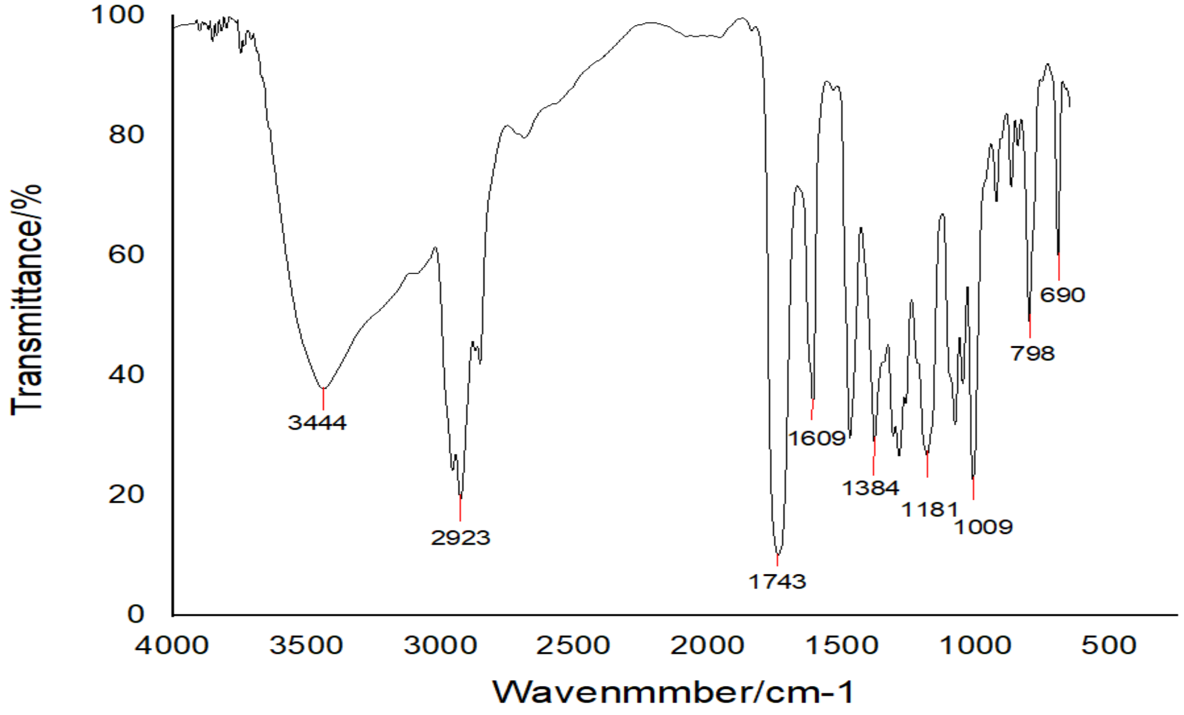

Figure S8. The IR spectrum of compound **1**

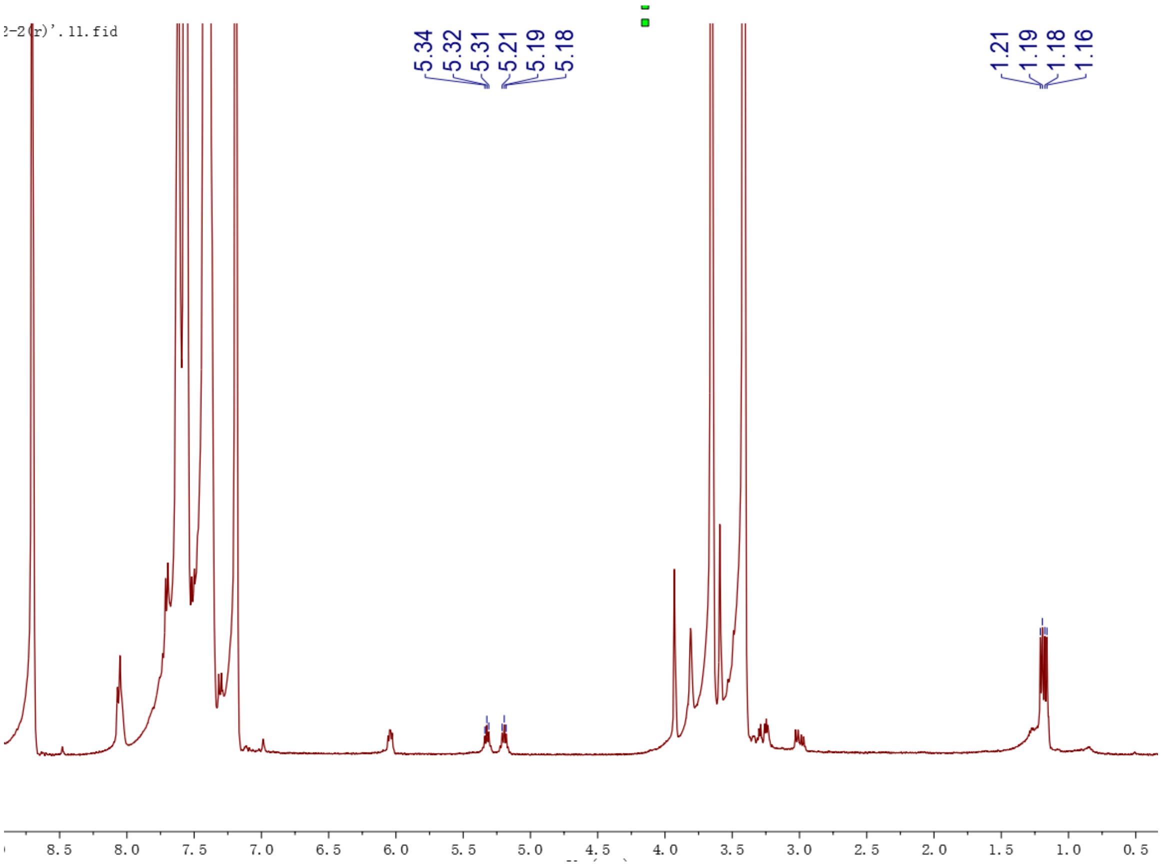

Figure S9. ^{1}H (400 MHz) NMR spectrum of **1a** in pyridine-d_5

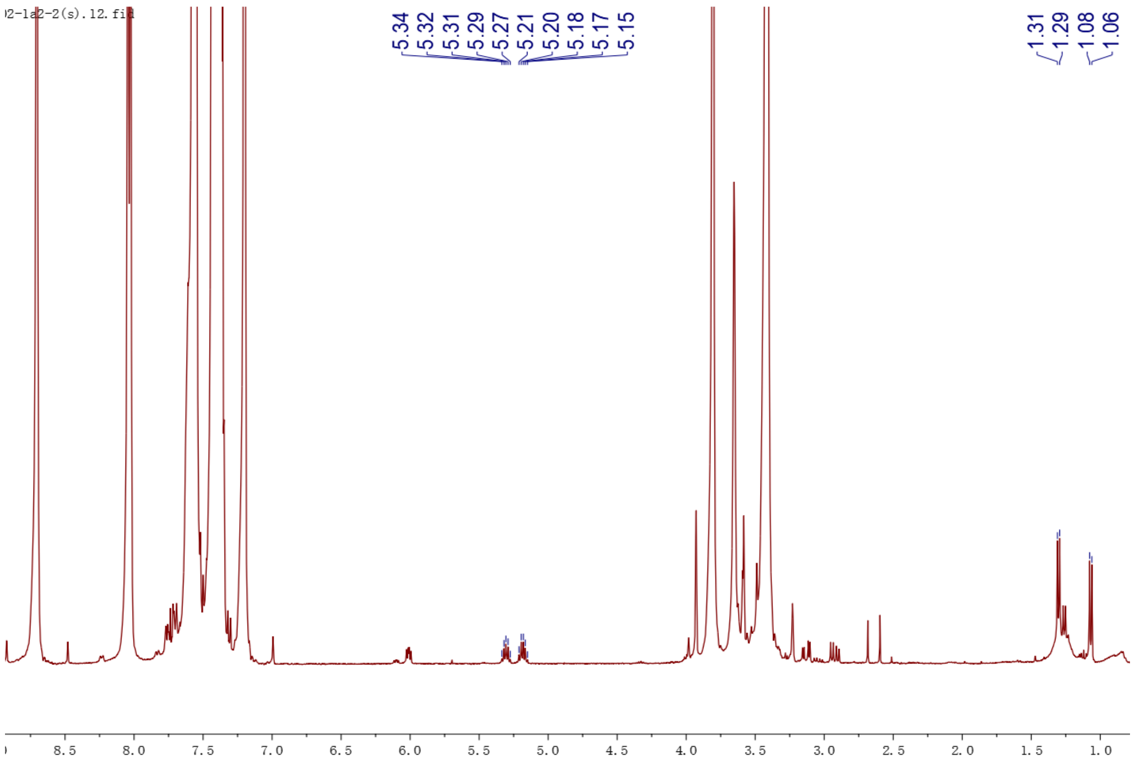

Figure S10. ^{1}H (400 MHz) NMR spectrum of **1b** in pyridine-d_5

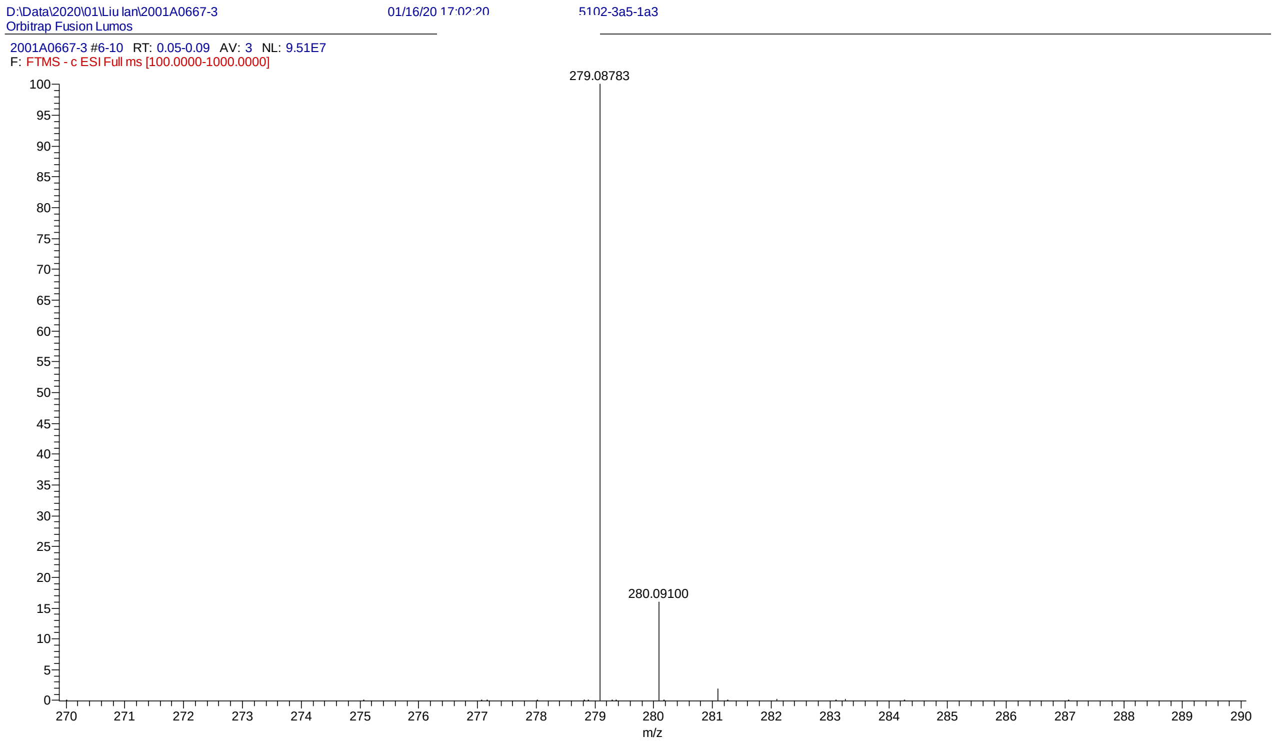

Figure S11. The HRESIMS spectrum of compound **2**.

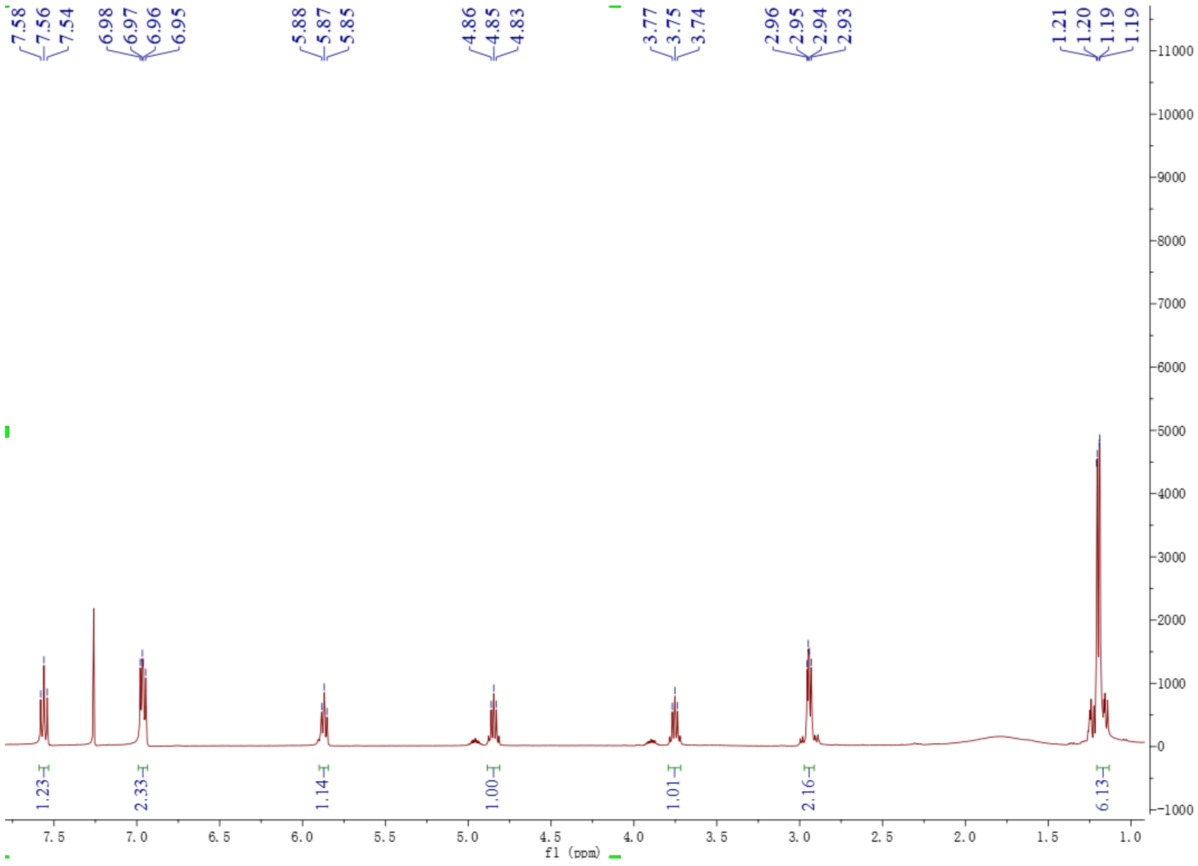

Figure S12. The ^{1}H NMR (400MHz) spectrum of compound **2** in $CDCl_3$.

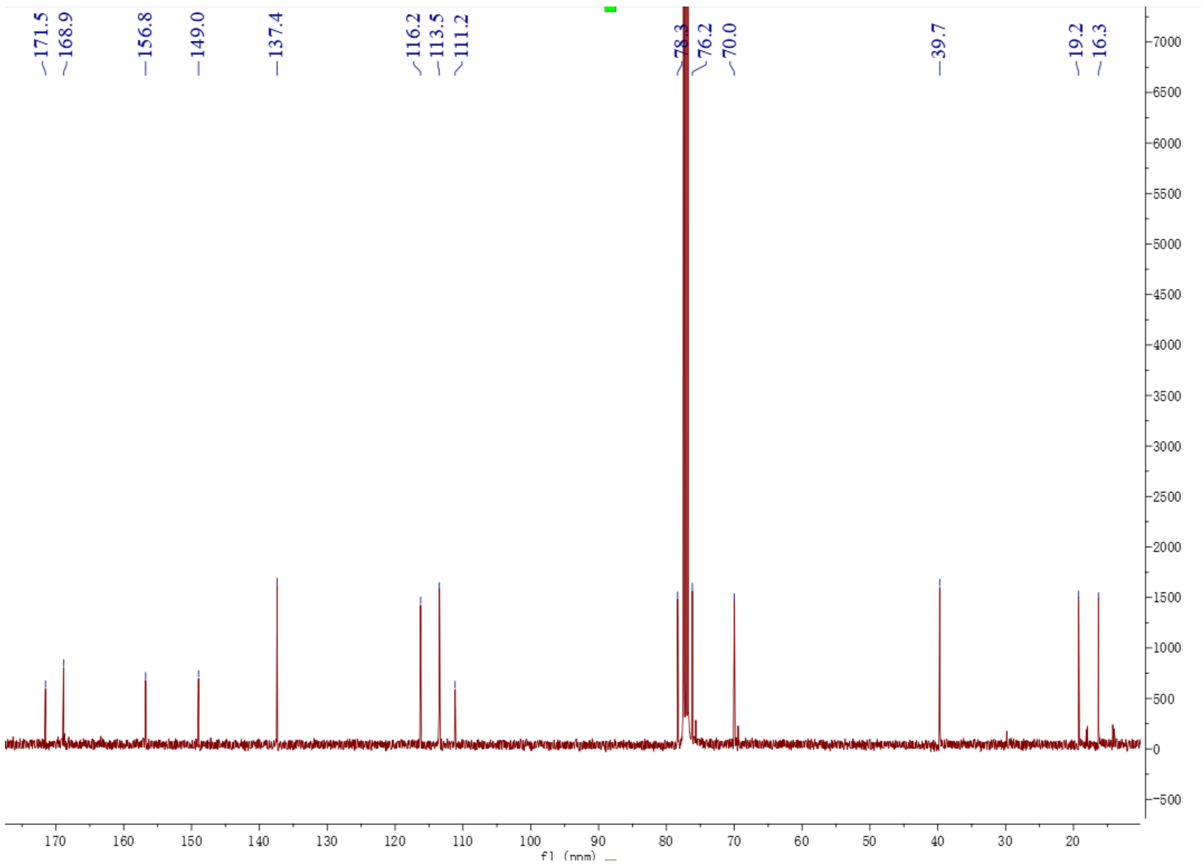

Figure S13. The ^{13}C NMR (100MHz) spectrum of compound **2** in $CDCl_3$.

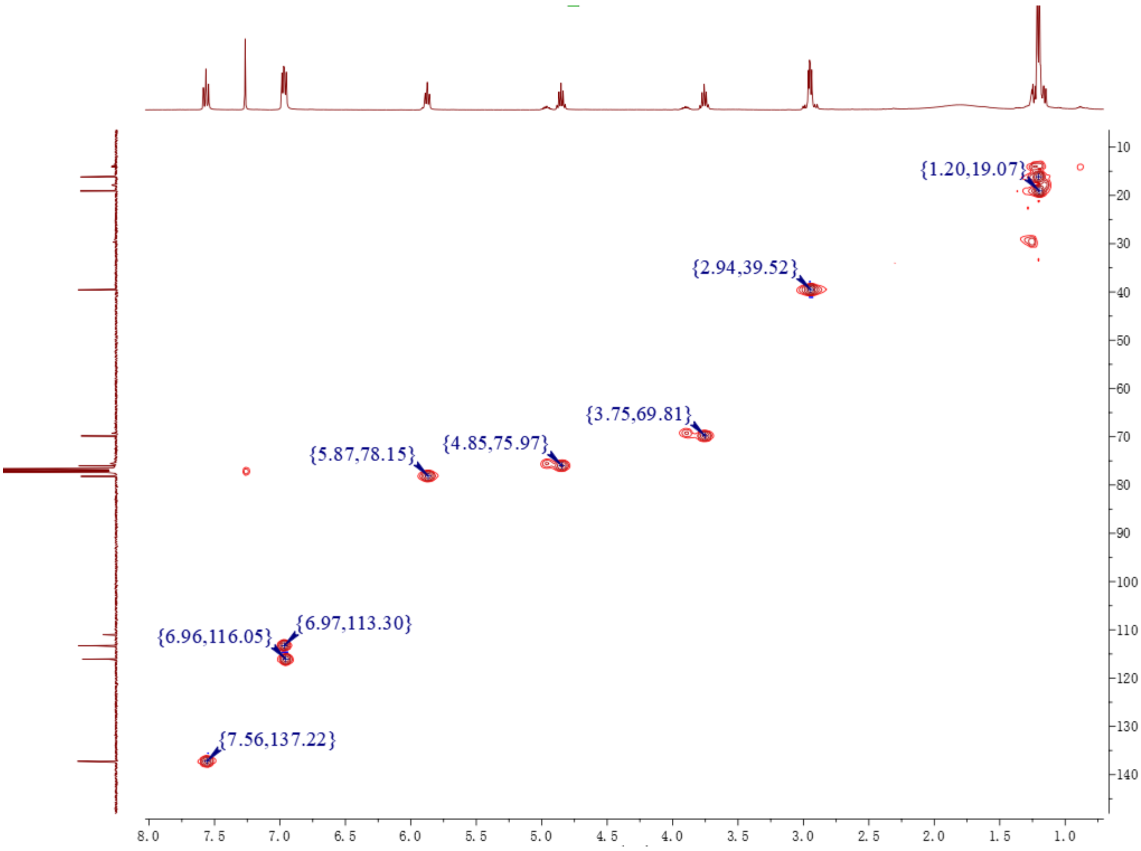

Figure S14. The HSQC spectrum of compound **2** in $CDCl_3$.

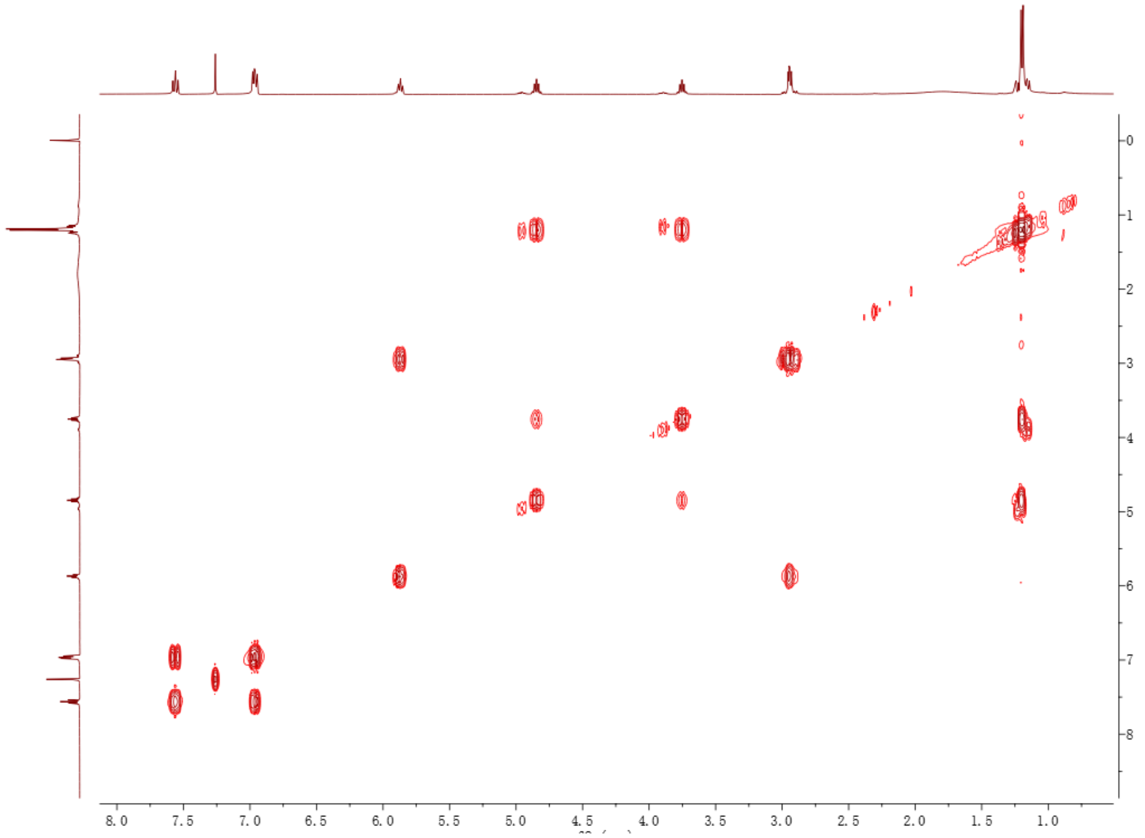

Figure S15. The ^{1}H-^{1}H COSY spectrum of compound **2** in $CDCl_3$.

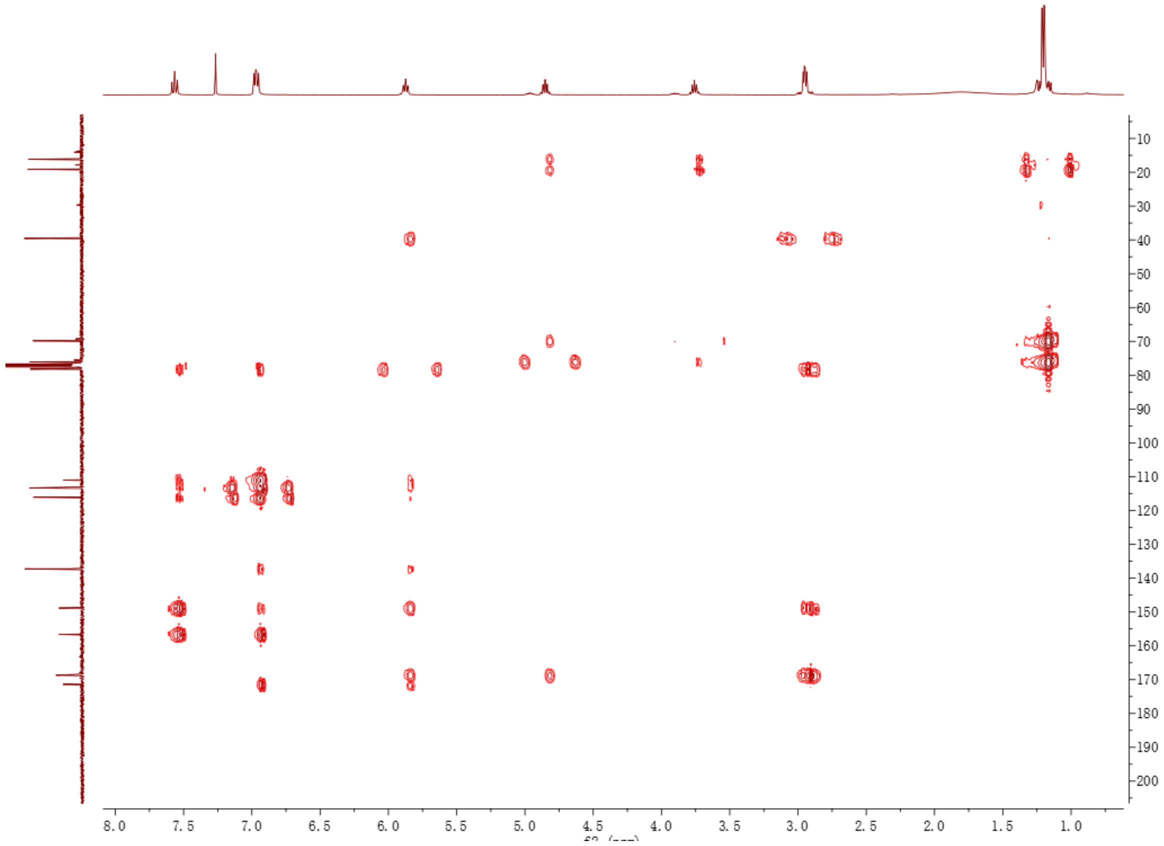

Figure S16. The HMBC spectrum of compound **2** in $CDCl_3$.

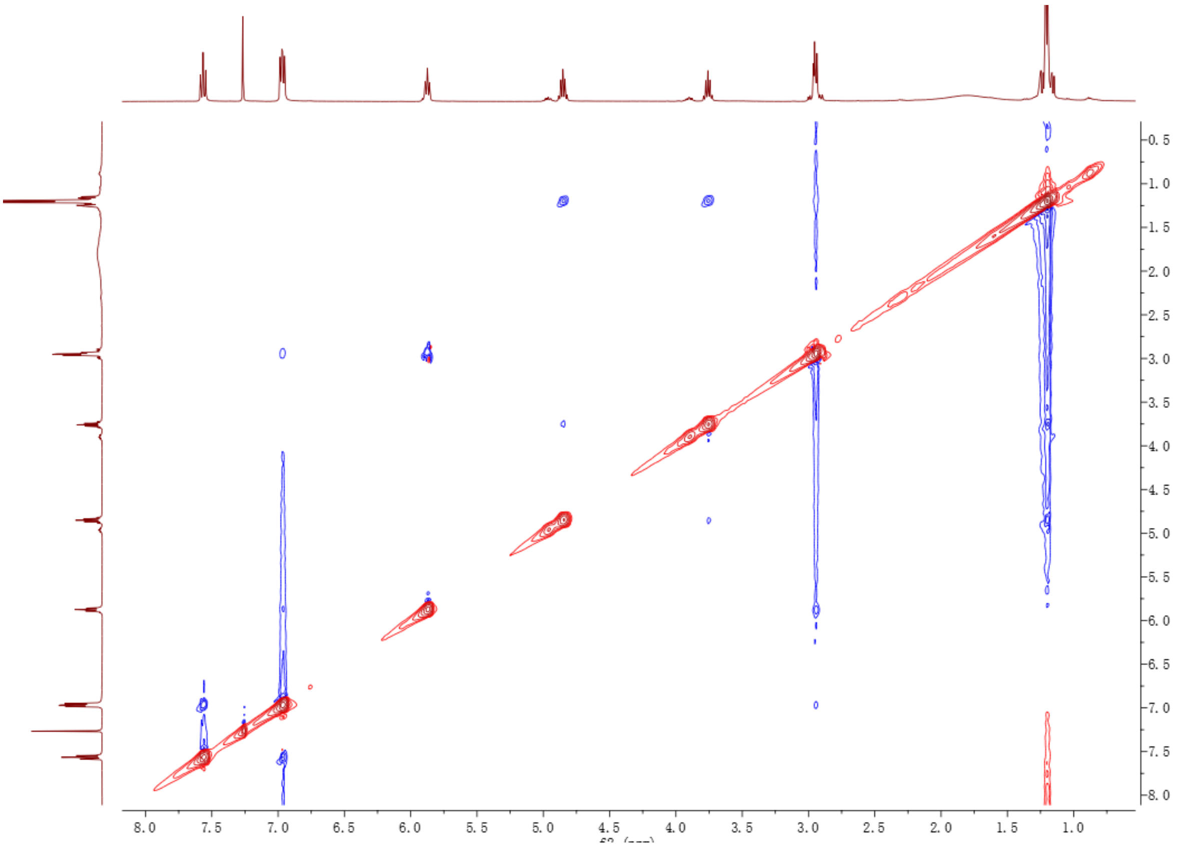

Figure S17. The NOESY spectrum of compound **2** in $CDCl_3$.

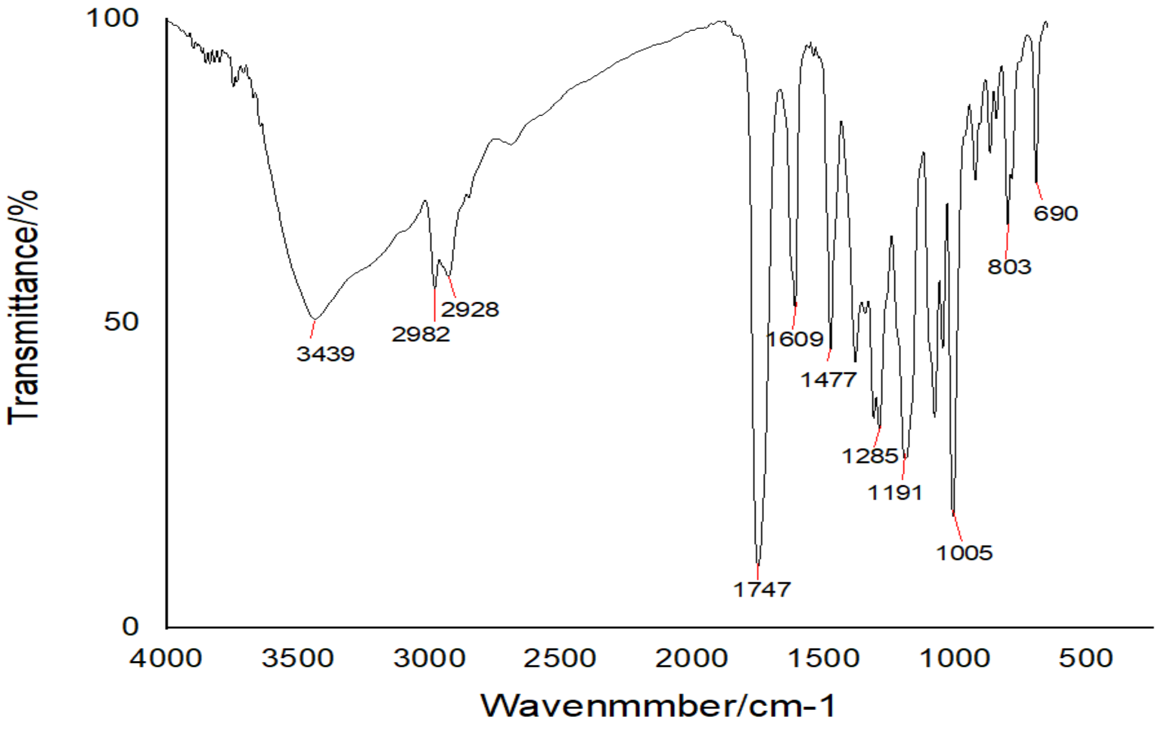

Figure S18. IR spectrum of compound **2**.

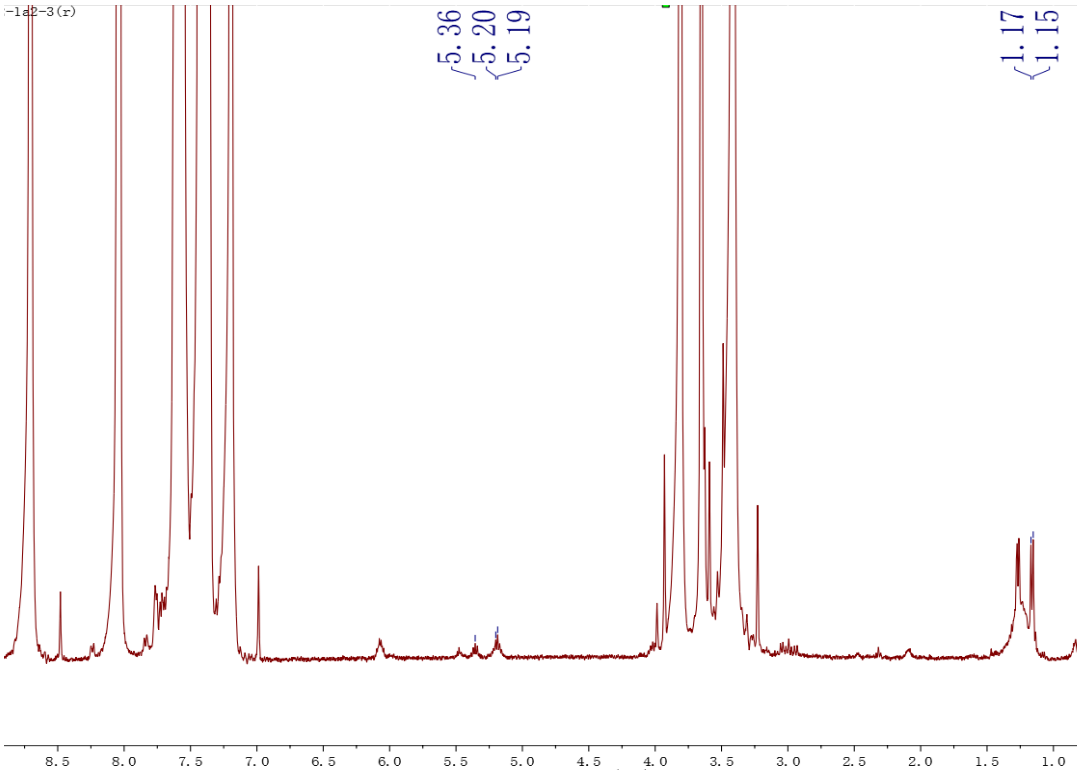

Figure S19. ^{1}H (400 MHz) NMR spectrum of **2a** in pyridine-d_5

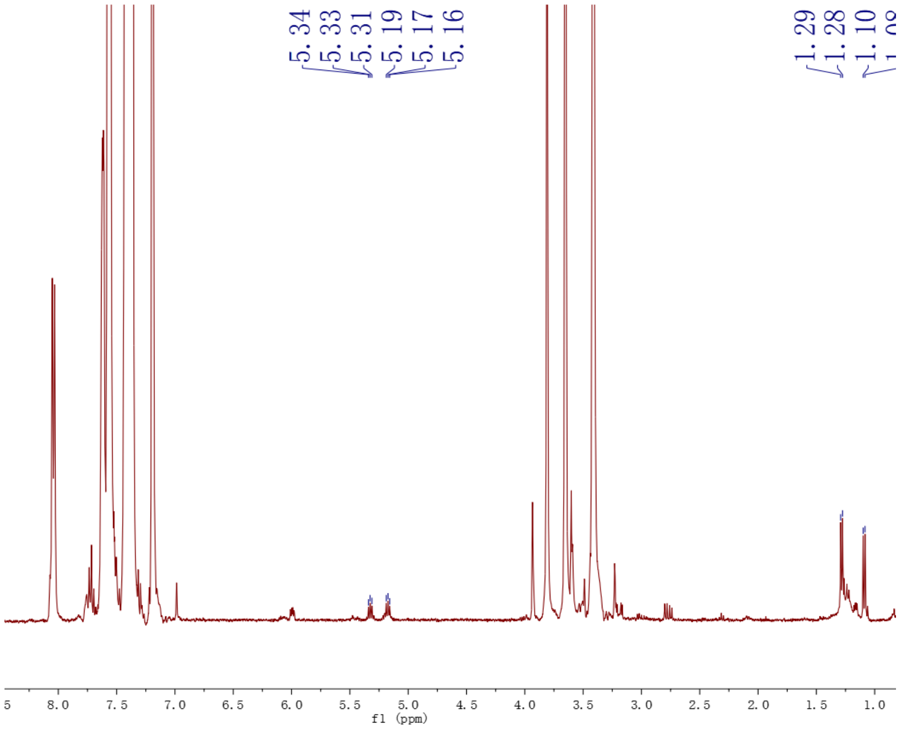

Figure S20. ^{1}H (400 MHz) NMR spectrum of **2b** in pyridine-d_5

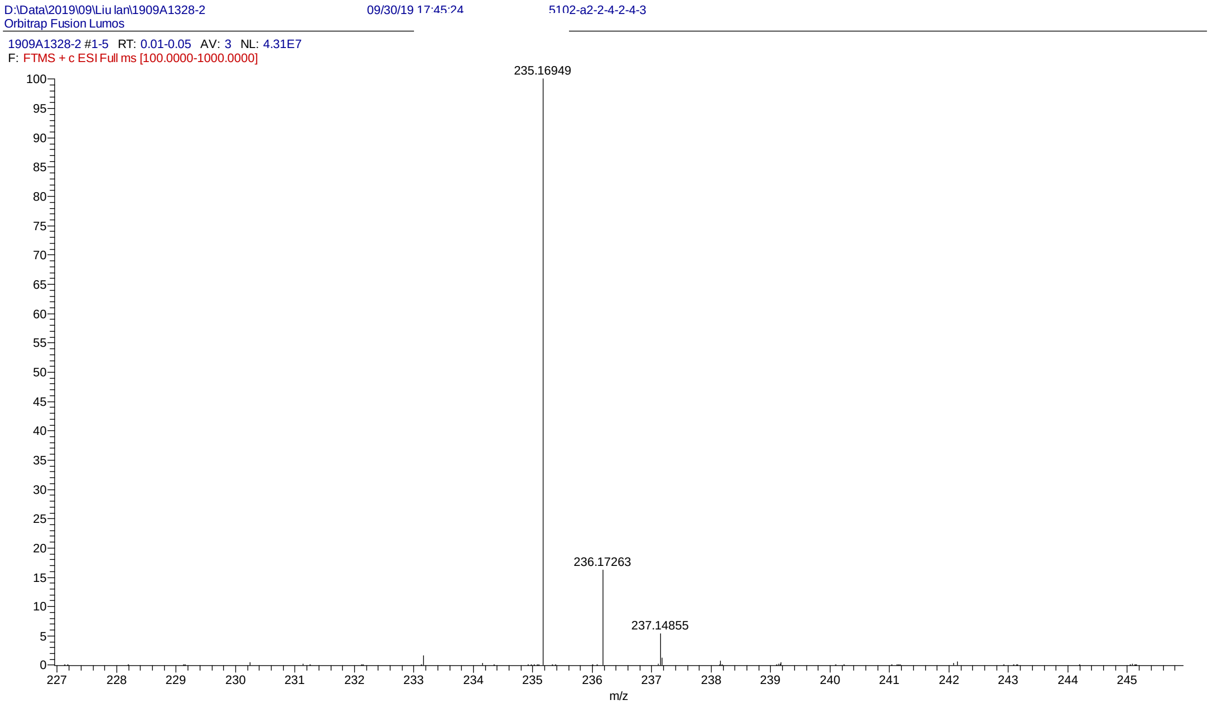

Figure S21. The HRESI-MS spectrum of compound **3**.

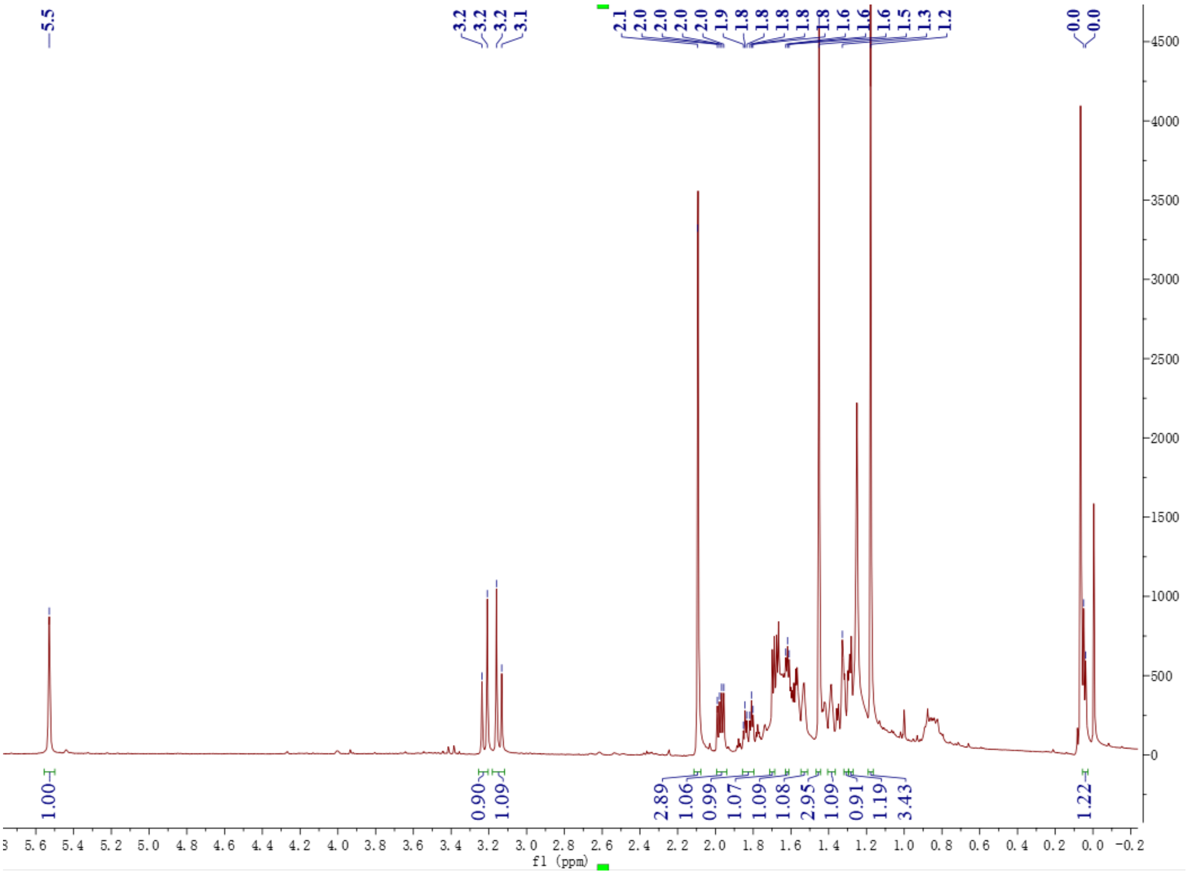

Figure S22. The ^{1}H NMR (400MHz) spectrum of compound **3** in $CDCl_3$.

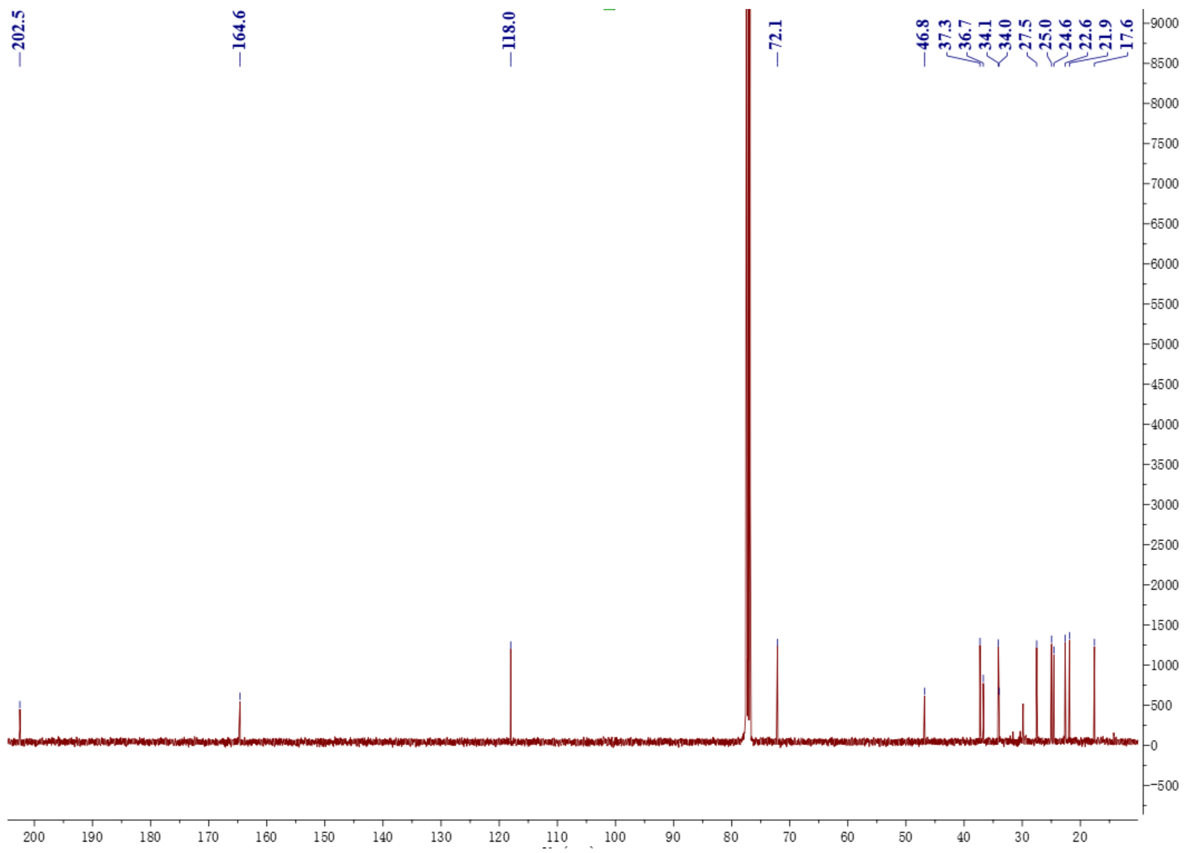

Figure S23. The ^{13}C NMR (100MHz) spectrum of compound **3** in $CDCl_3$.

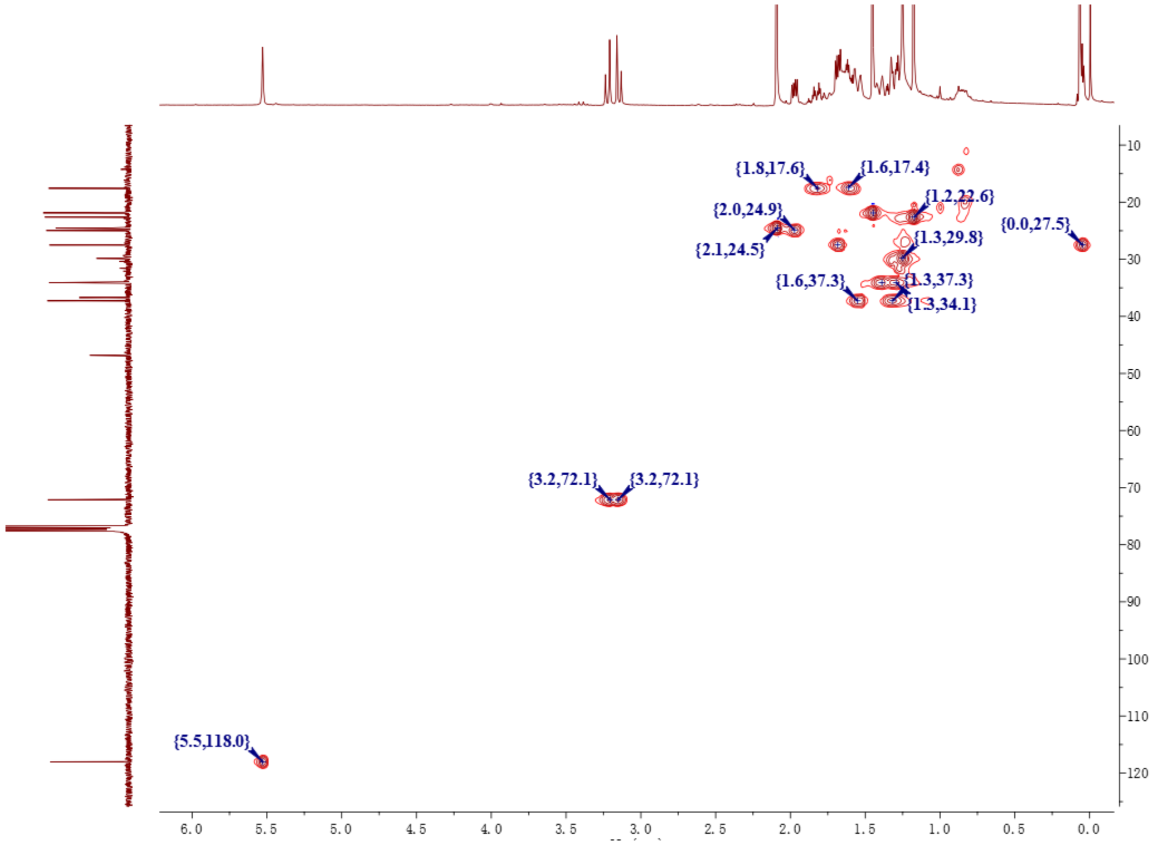

Figure S24. The HSQC spectrum of compound **3** in $CDCl_3$

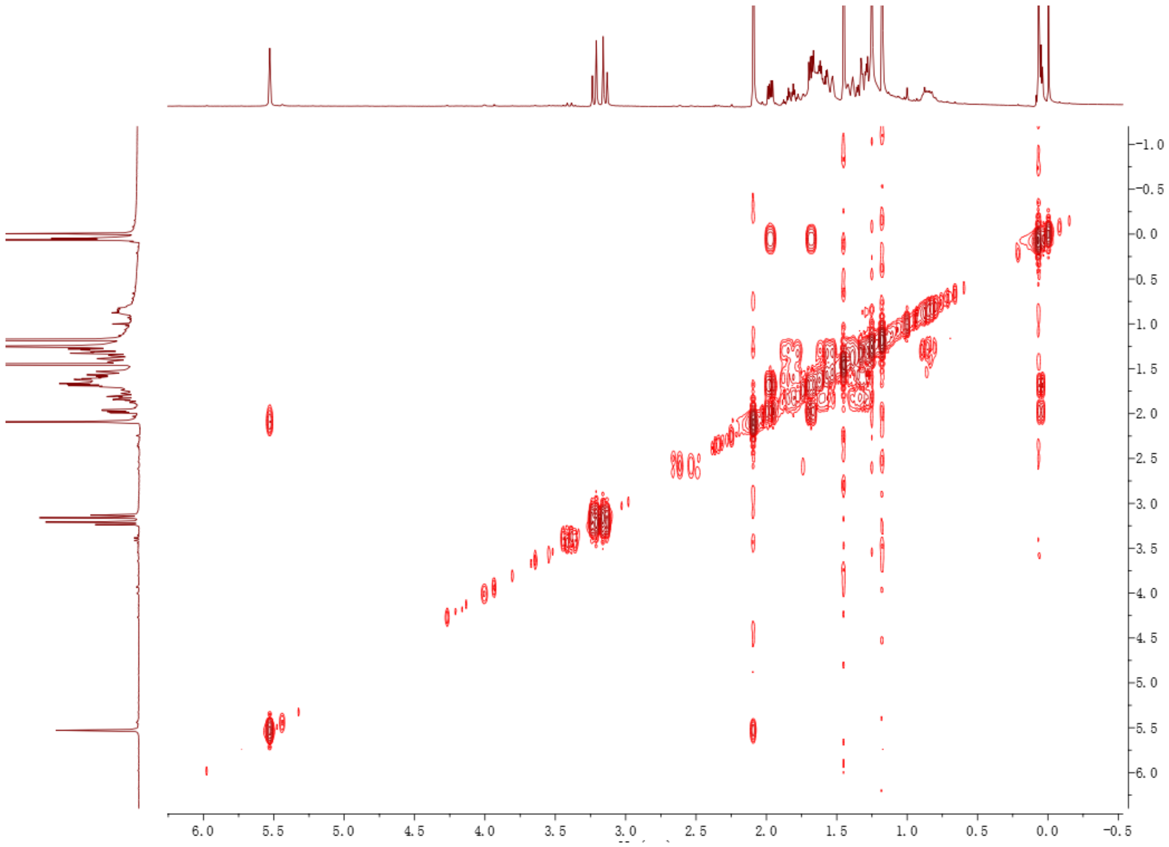

Figure S25. The ^{1}H-^{1}H COSY spectrum of compound **3** in $CDCl_3$.

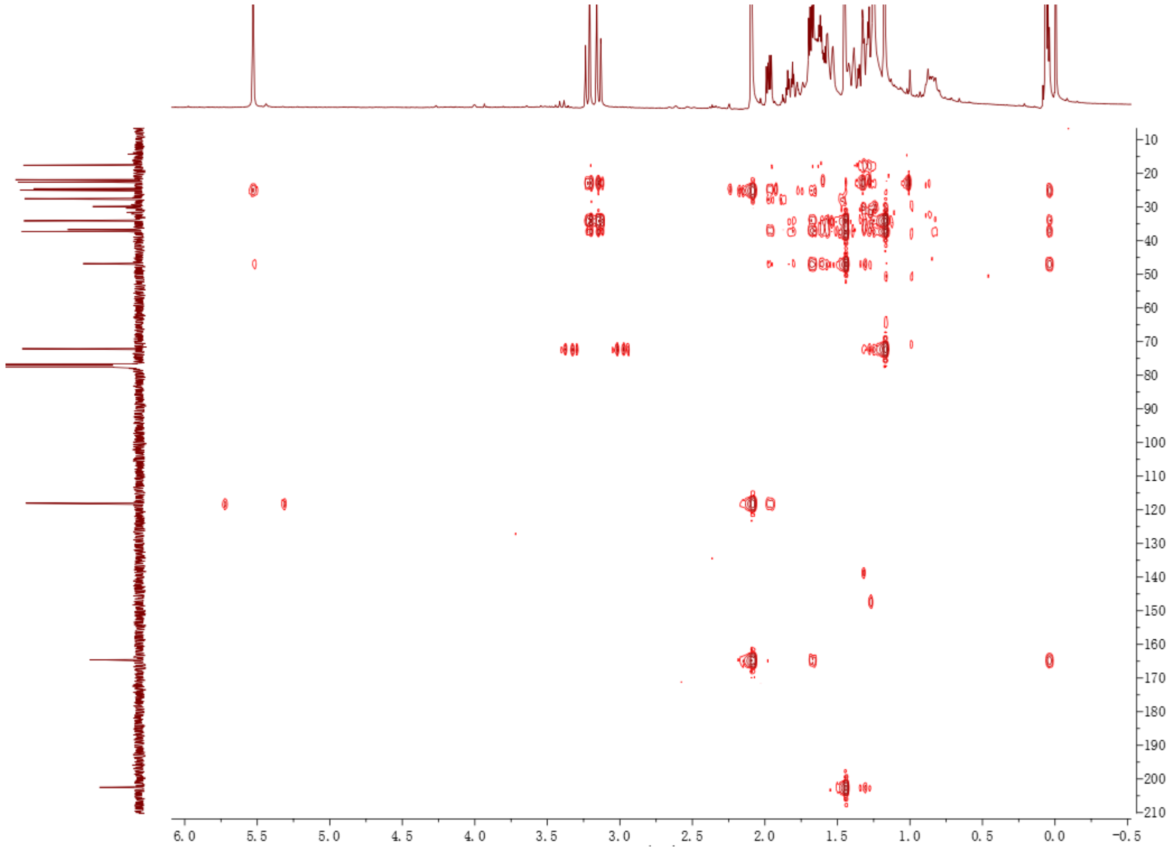

Figure S26. The HMBC spectrum of compound **3** in $CDCl_3$.

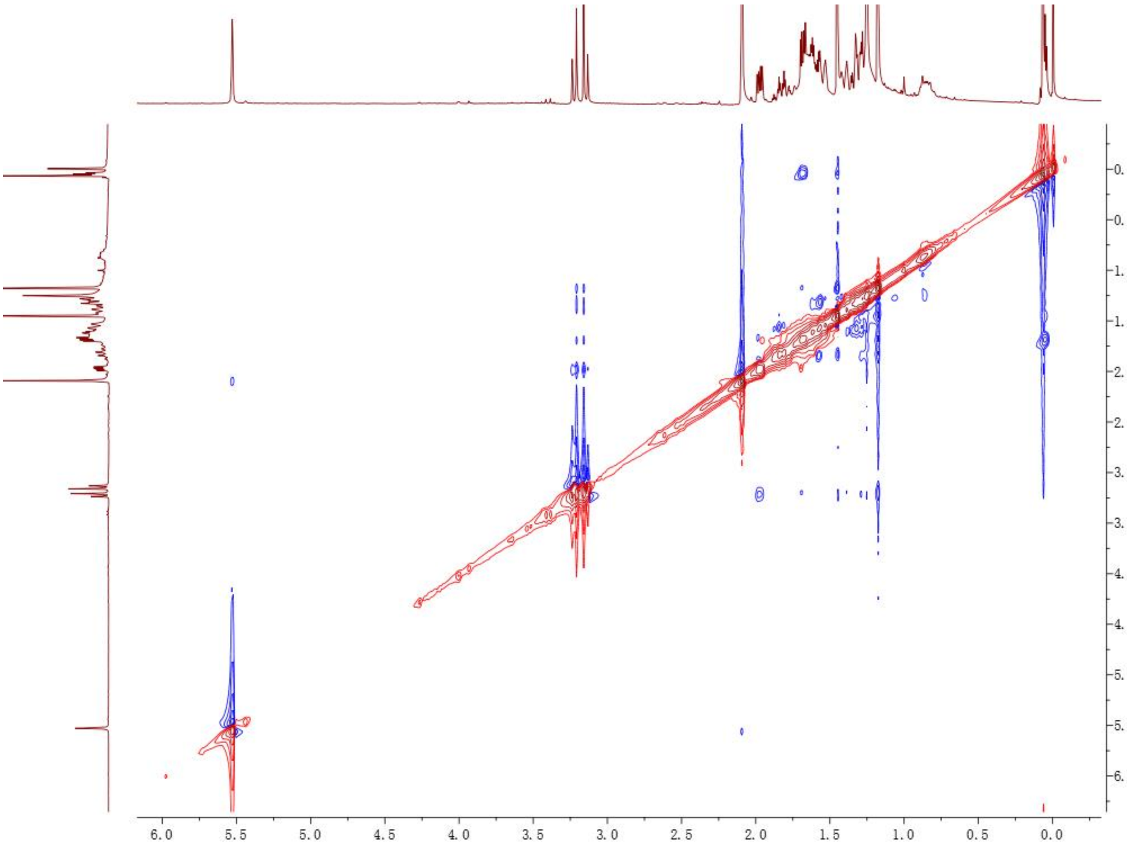

Figure S27. The NOESY spectrum of compound **3** in $CDCl_3$.

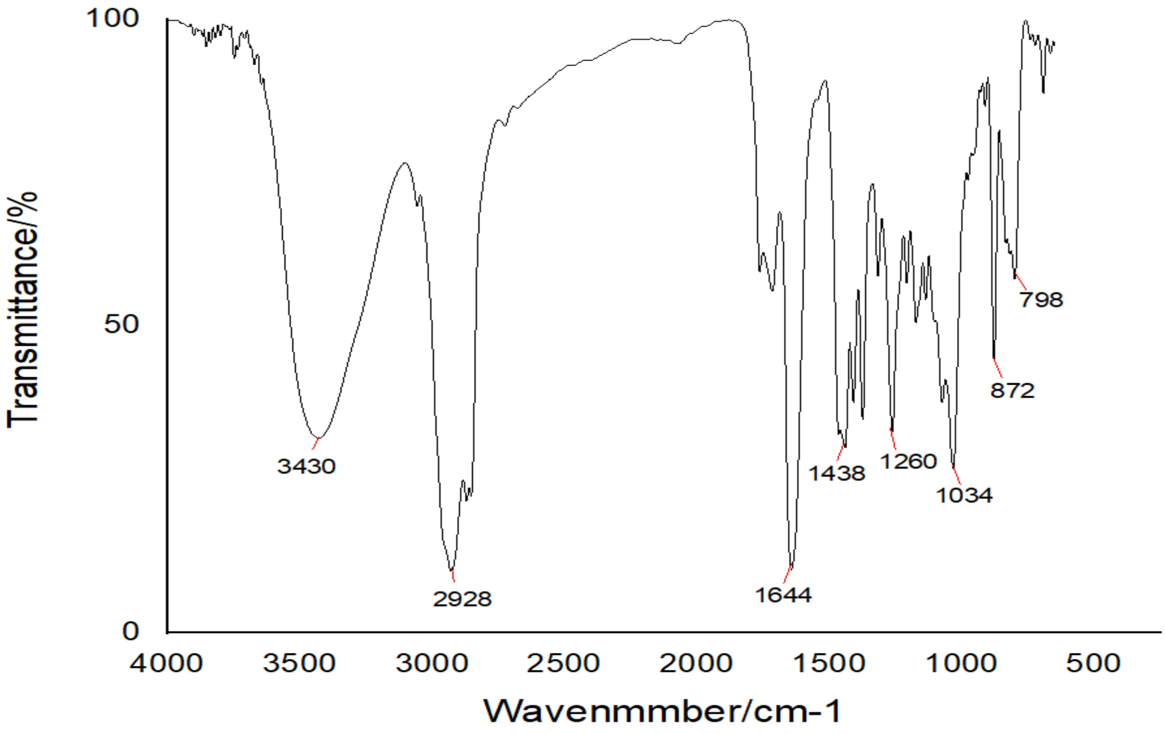

Figure S28. The IR spectrum of compound **3**.

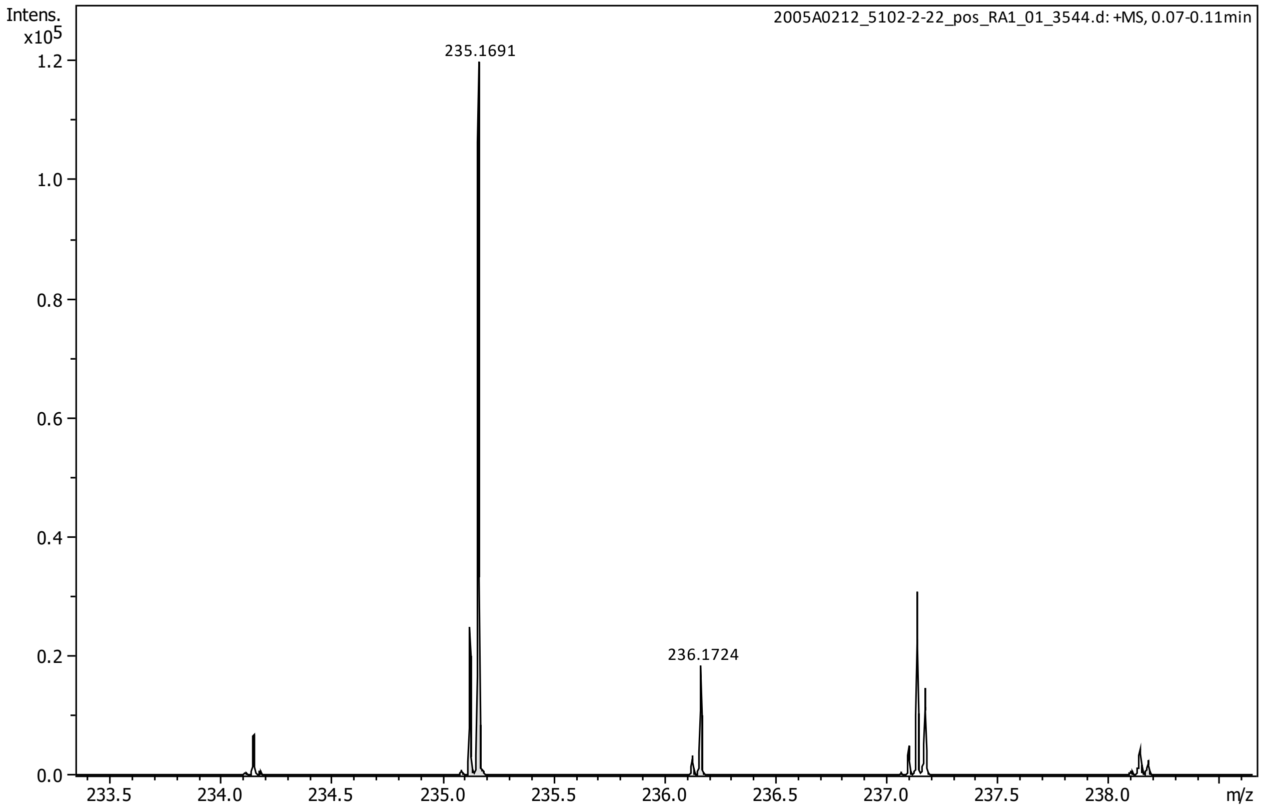

Figure S29. The HRESI-MS/MS spectrum of compound **4**.

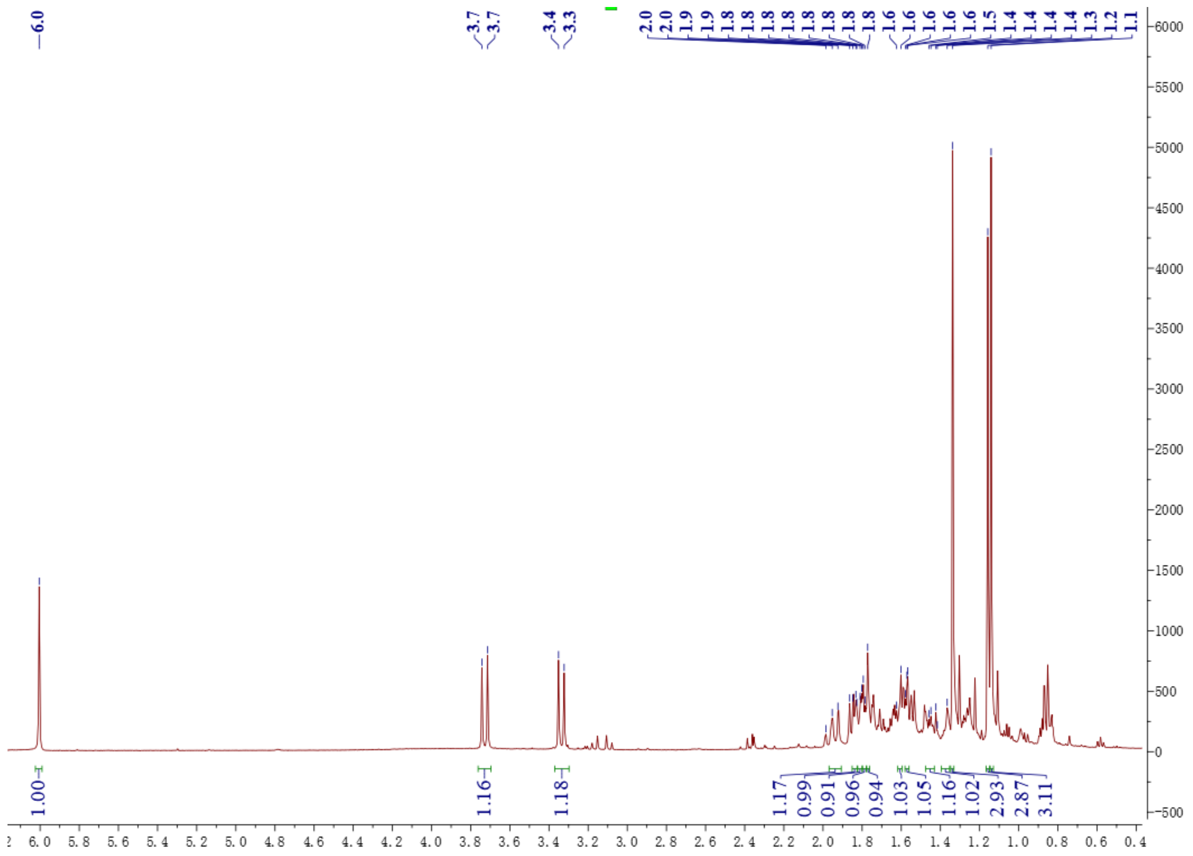

Figure S30. The ^{1}H NMR (400MHz) spectrum of compound **4** in $CDCl_3$.

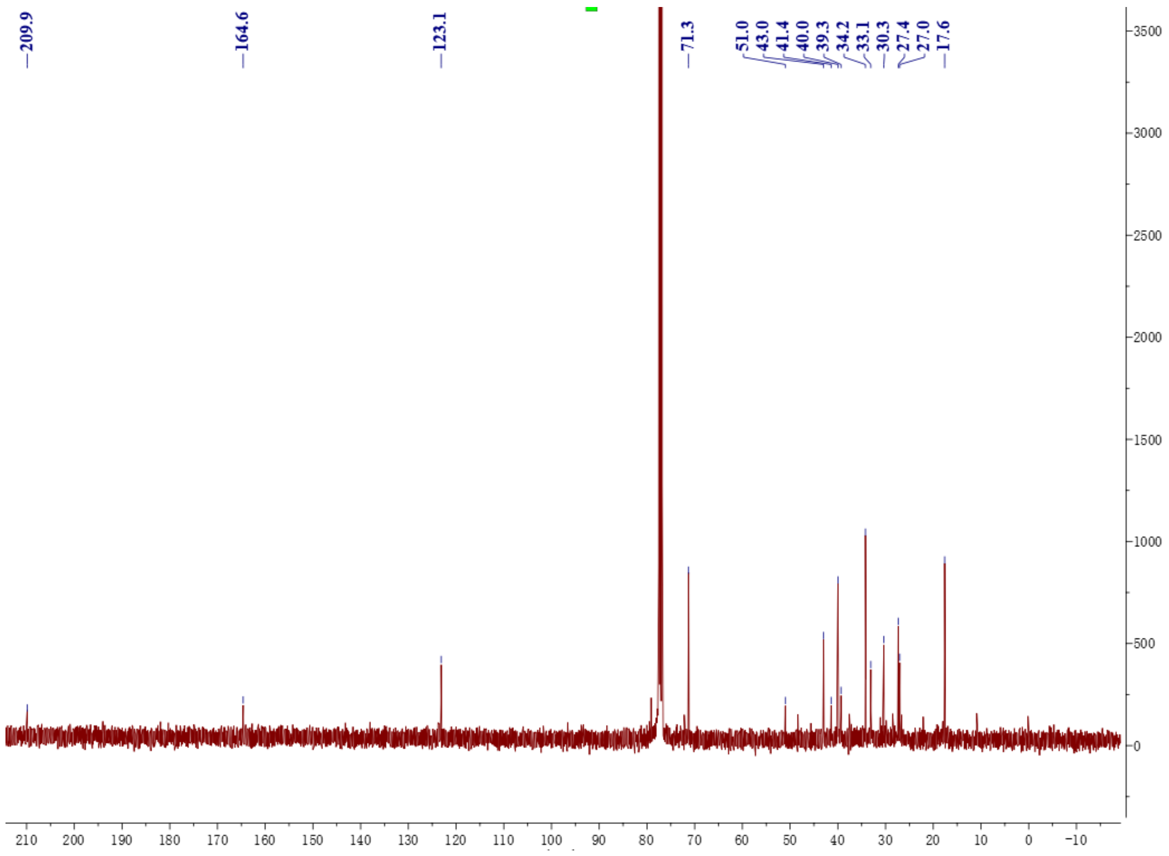

Figure S31. The ^{13}C NMR (100MHz) spectrum of compound **4** in $CDCl_3$.

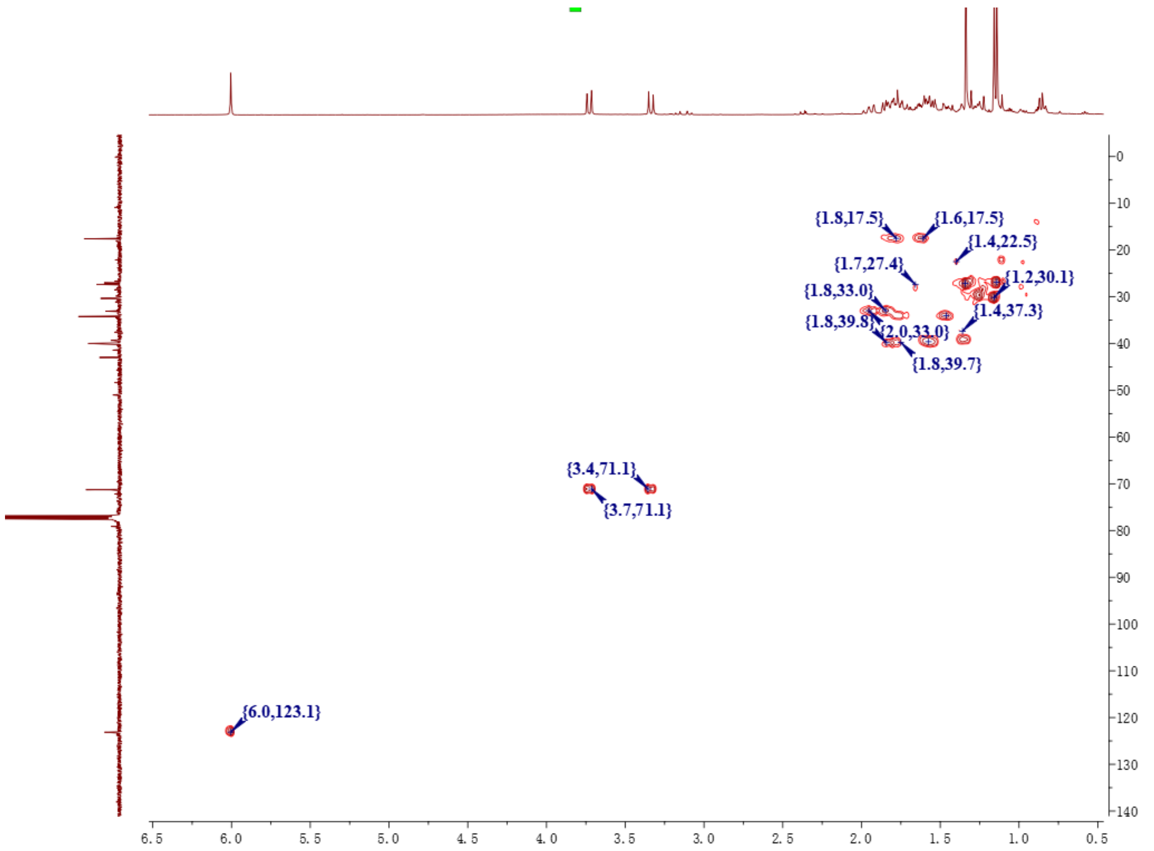

Figure S32. The HSQC spectrum of compound **4** in $CDCl_3$-*d*.

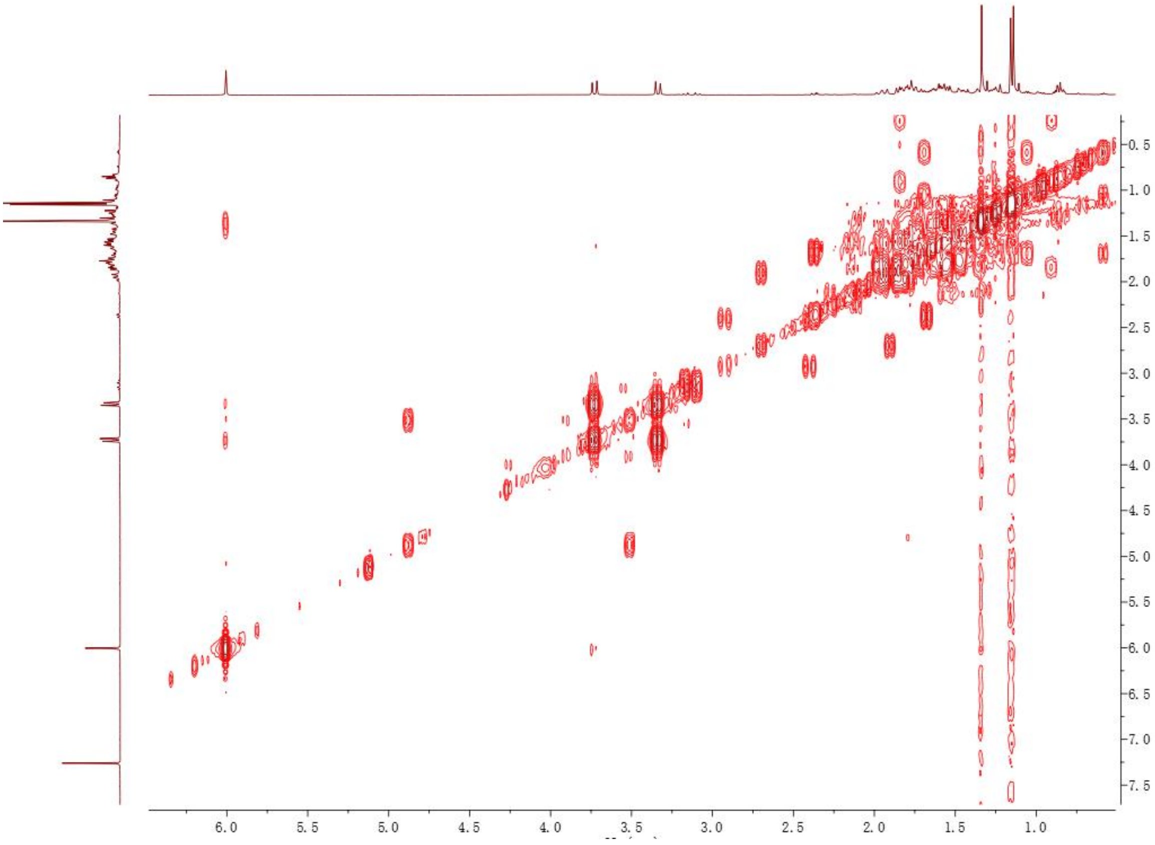

Figure S33. The ^1H-^1H COSY spectrum of compound **4** in $CDCl_3$-*d*.

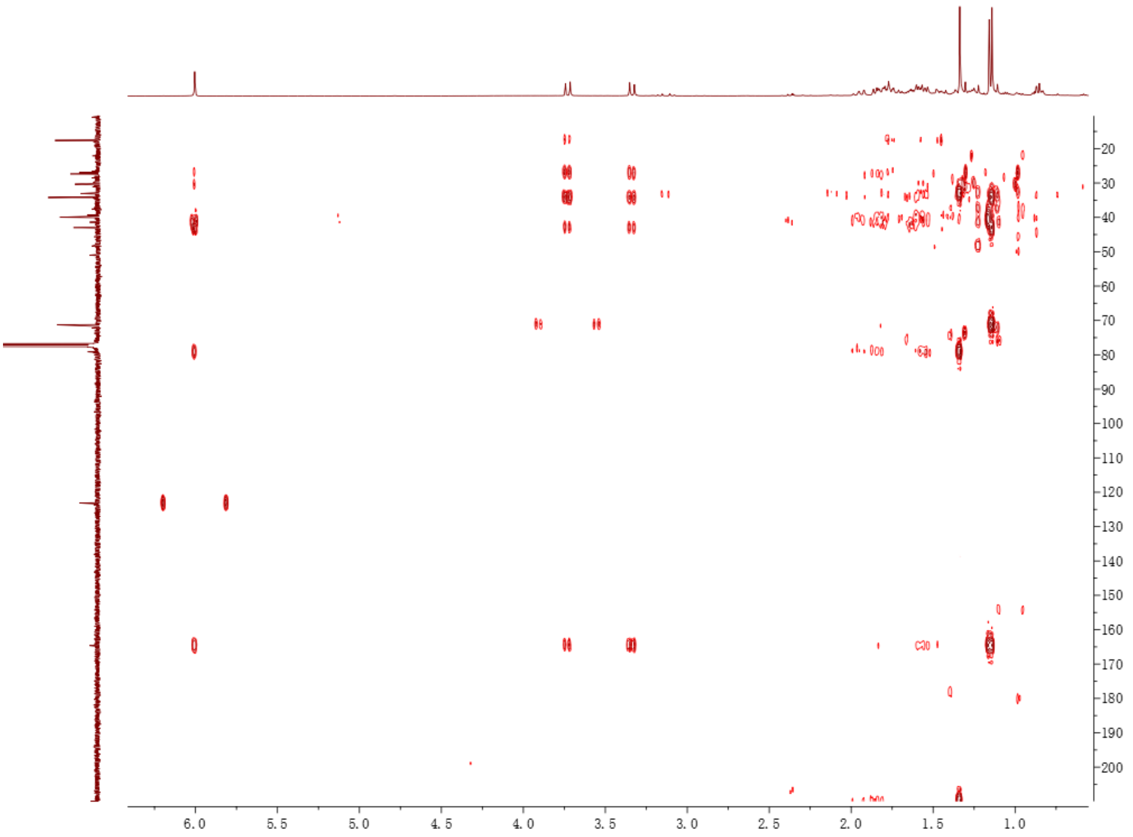

Figure 34. The HMBC spectrum of compound **4** in $CDCl_3$-*d*.

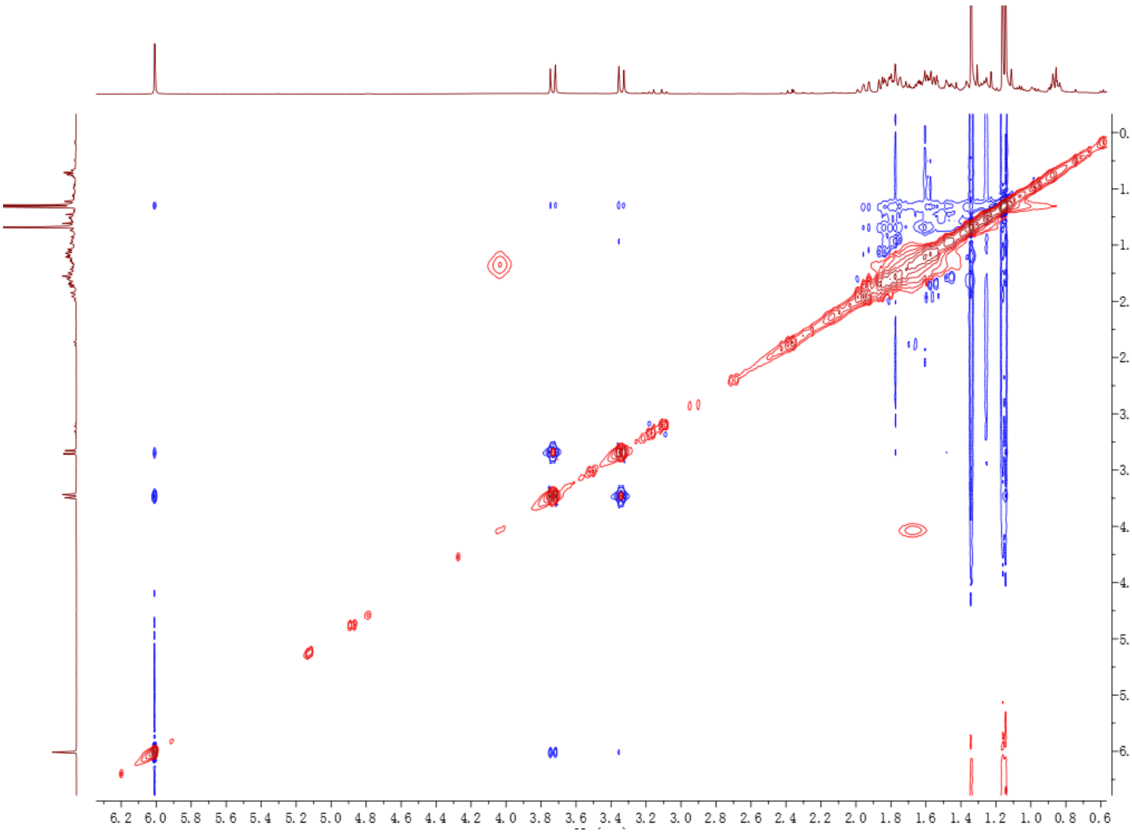

Figure 35. The NOESY spectrum of compound **4** in $CDCl_3$.

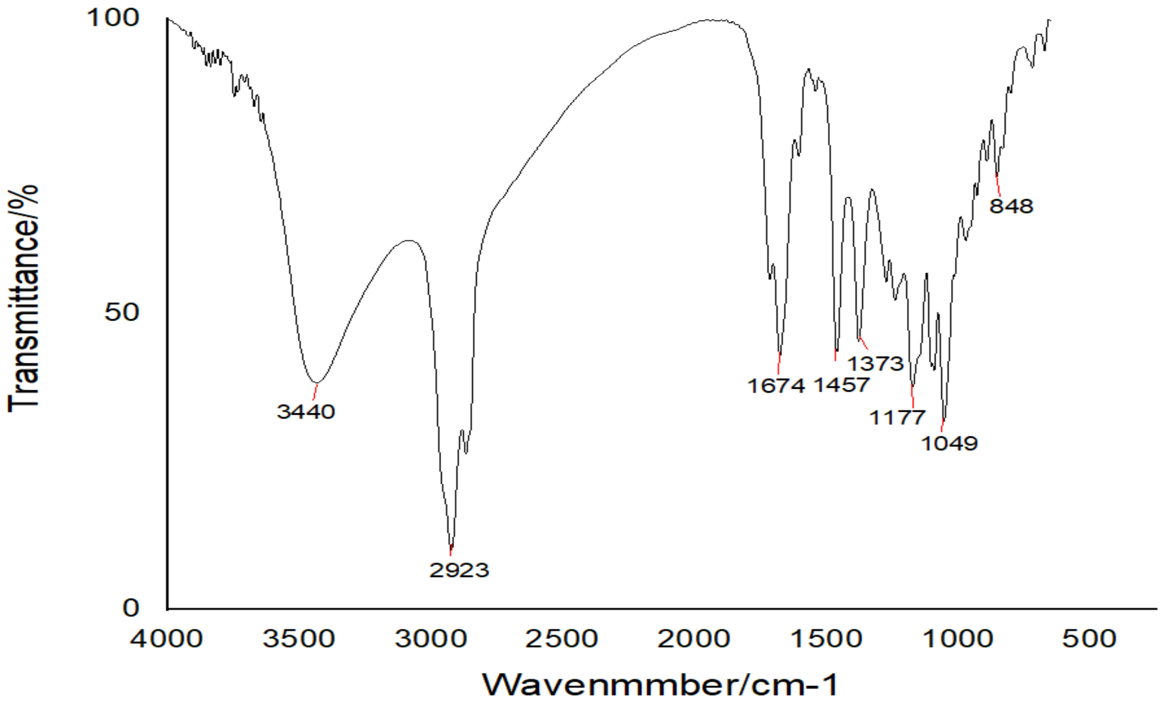

Figure 36. The IR spectrum of compound **4**.

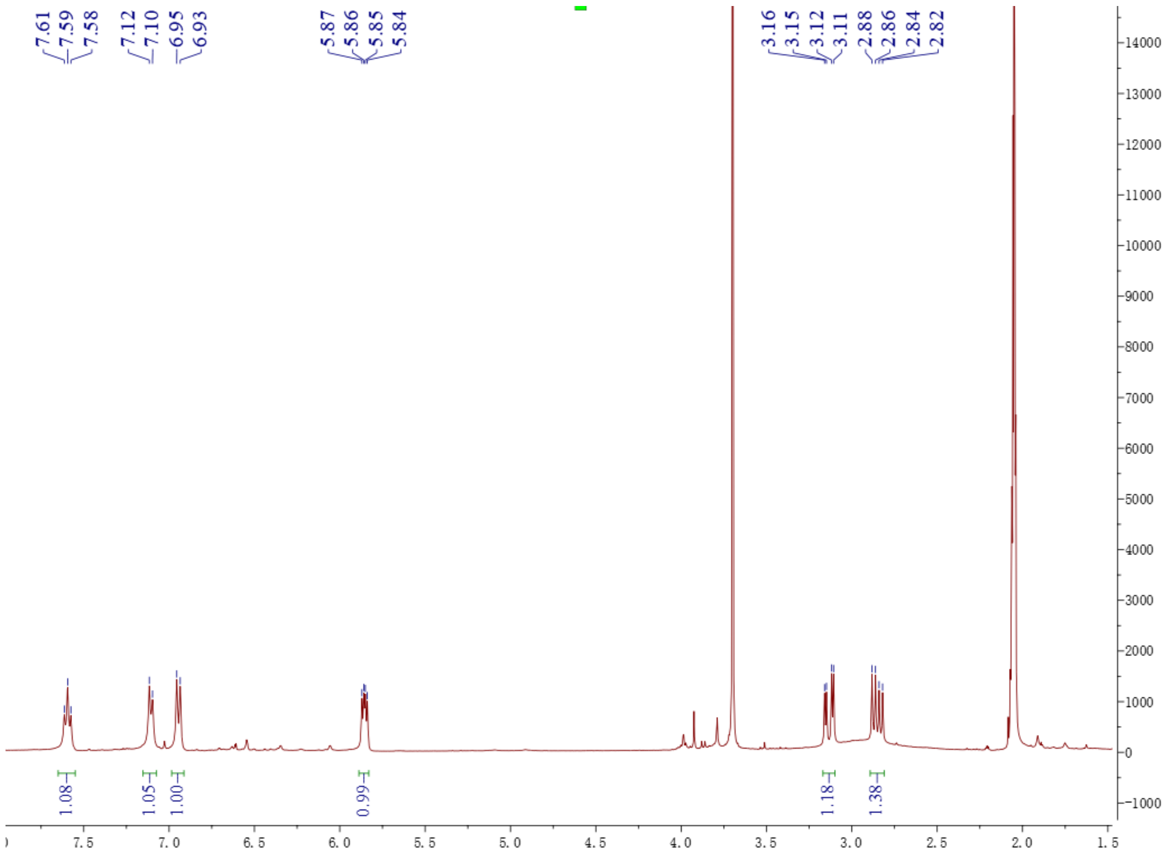

Figure 37. The ^{1}H NMR (400MHz) spectrum of compound **5** in acetone-d_6.

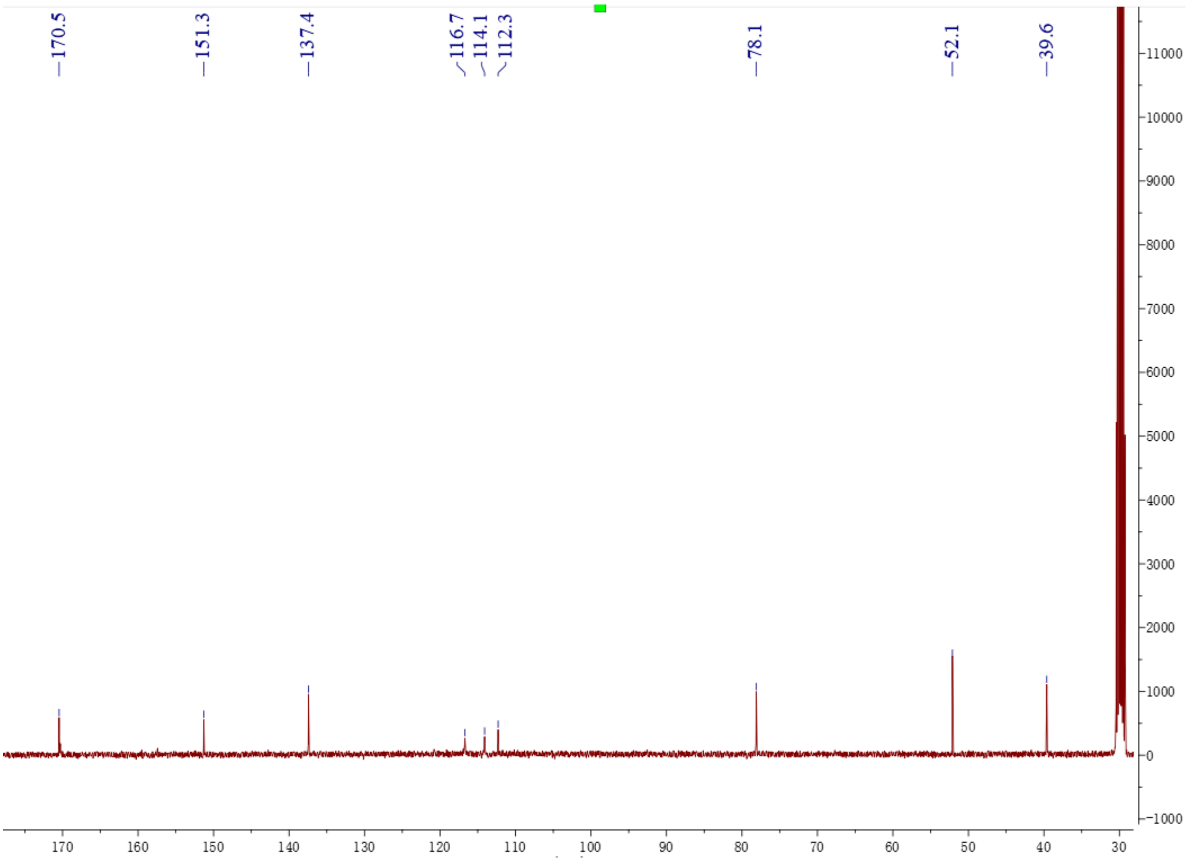

Figure 38. The ^{13}C NMR (100MHz) spectrum of compound **5** in acetone-d_6.

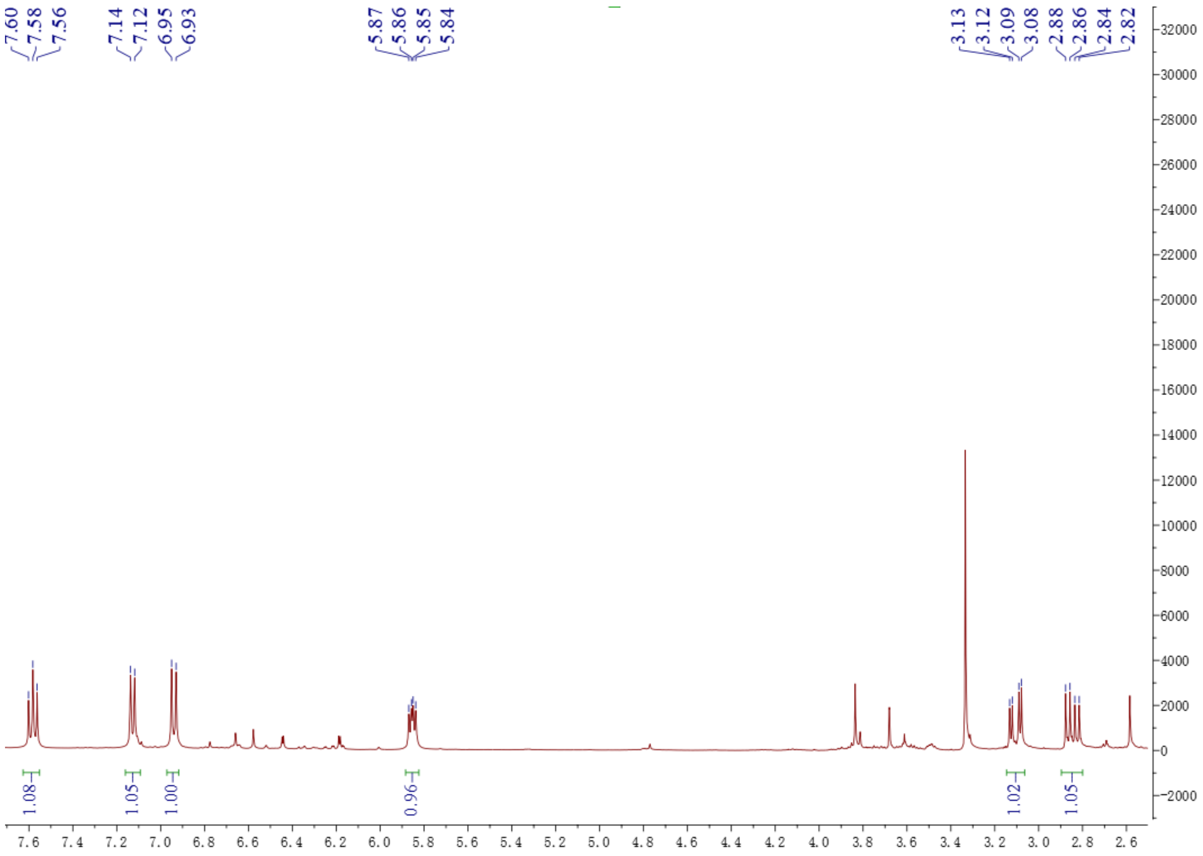

Figure 39. The ^{1}H NMR (400MHz) spectrum of compound **6** in acetone-d_6.

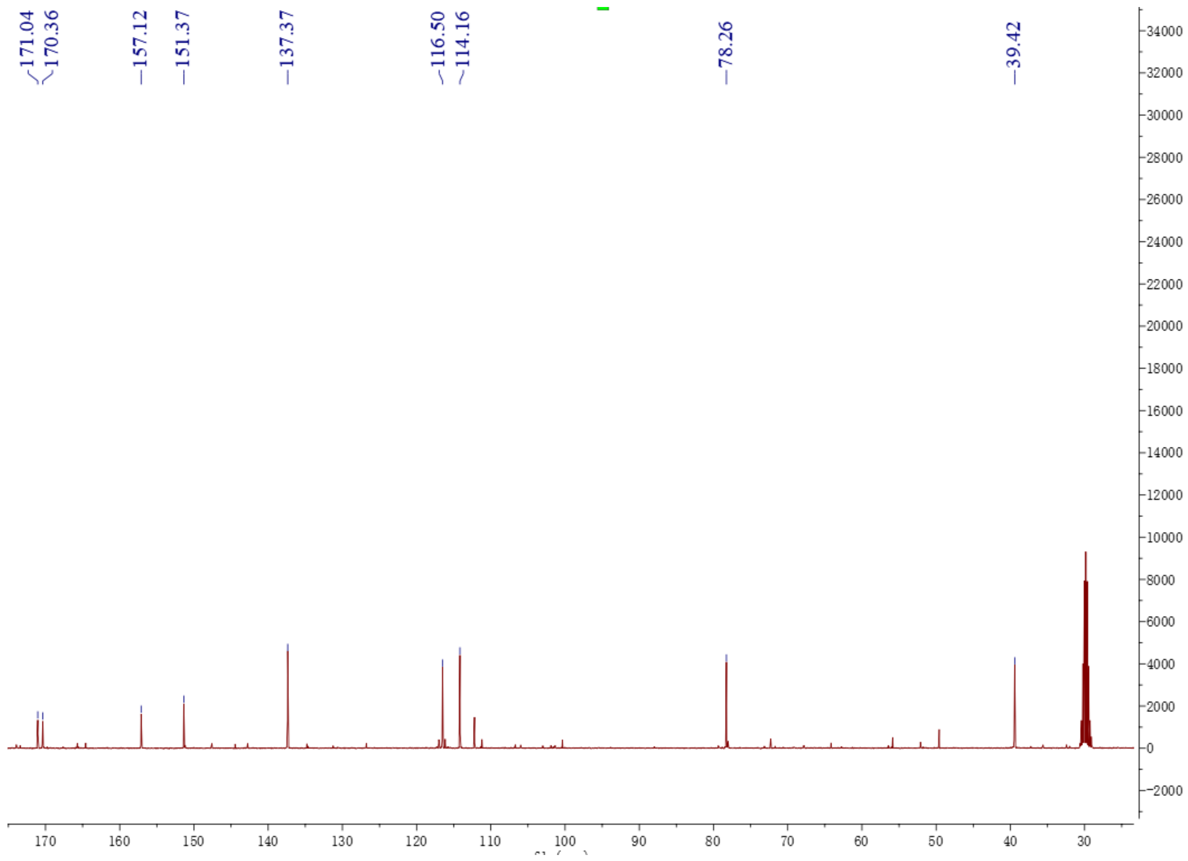

Figure 40. The ^{13}C NMR (100MHz) spectrum of compound **6** in acetone-d_6.

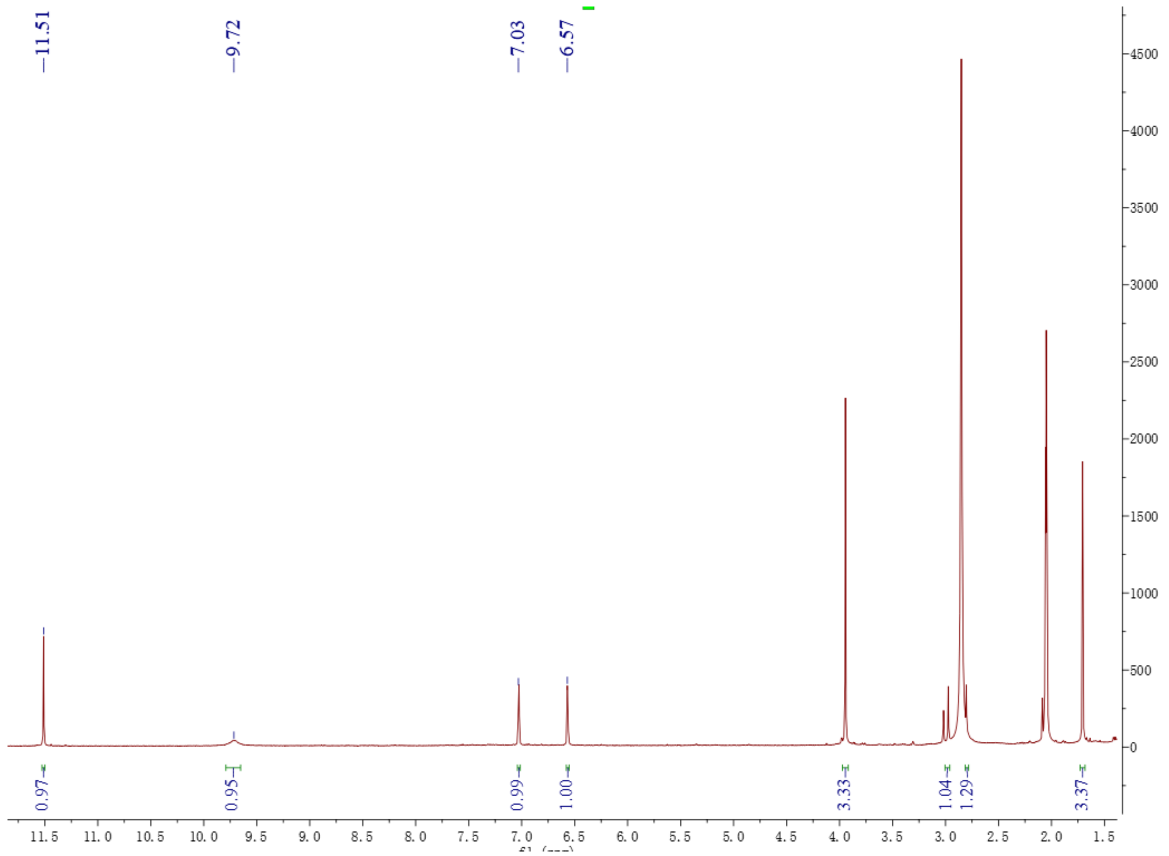

Figure 41. The ^{1}H NMR (400MHz) spectrum of compound **7** in acetone-d_6.

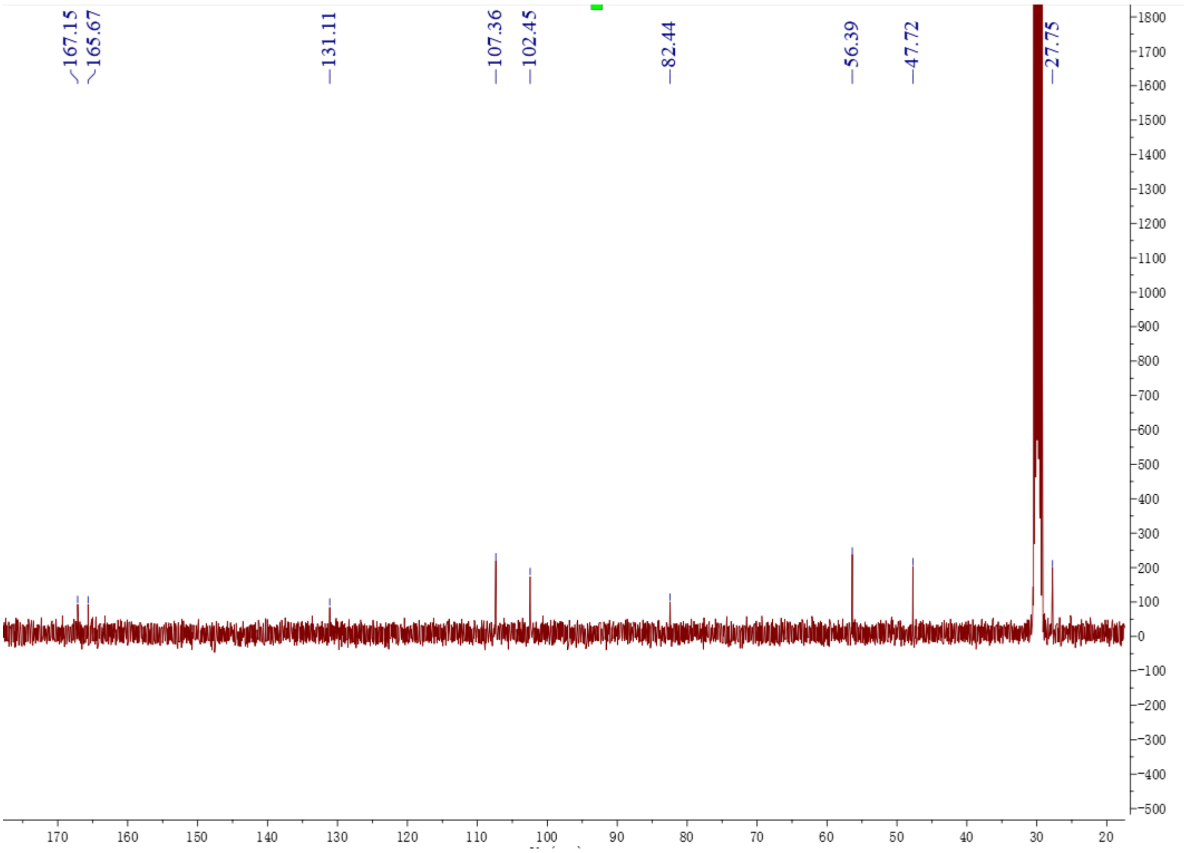

Figure 42. The ^{13}C NMR (100MHz) spectrum of compound **7** in acetone-d_6.

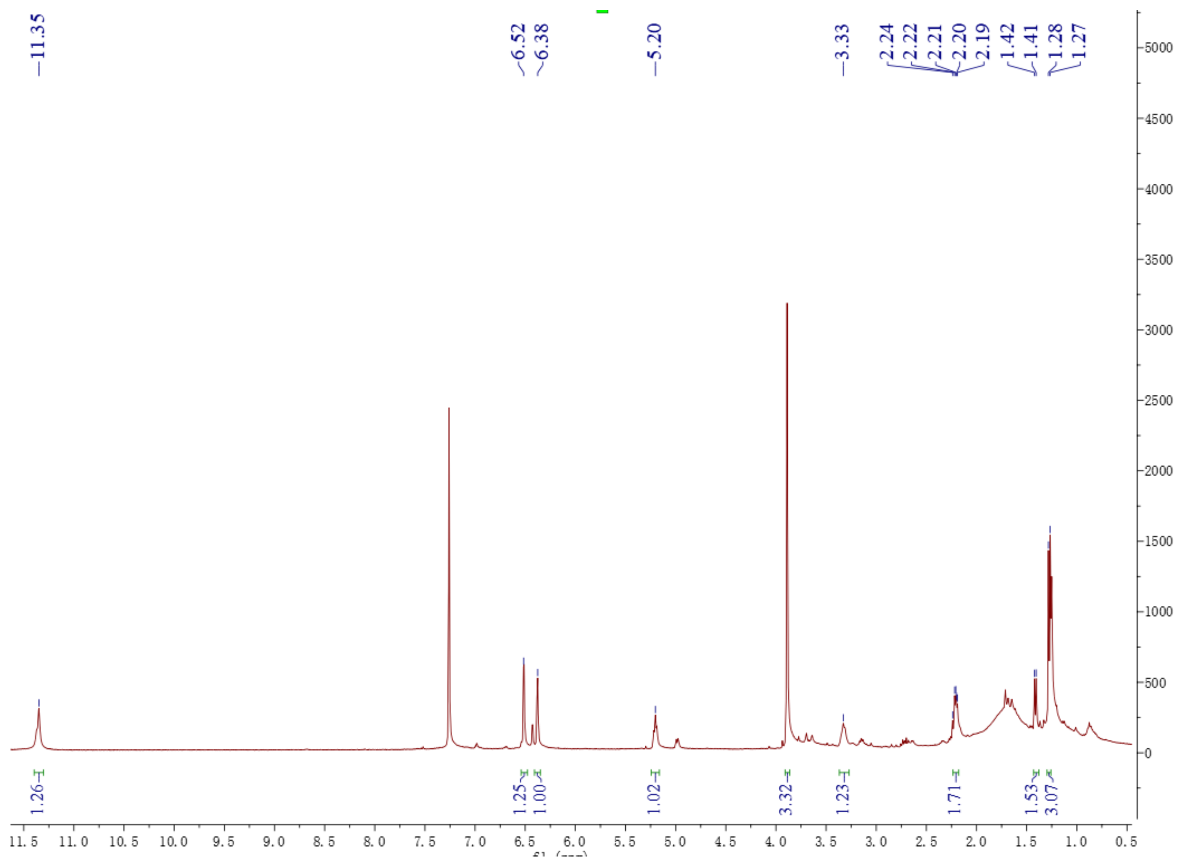

Figure 43. The ^{1}H NMR (400MHz) spectrum of compound **9** in $CDCl_3$-*d*.

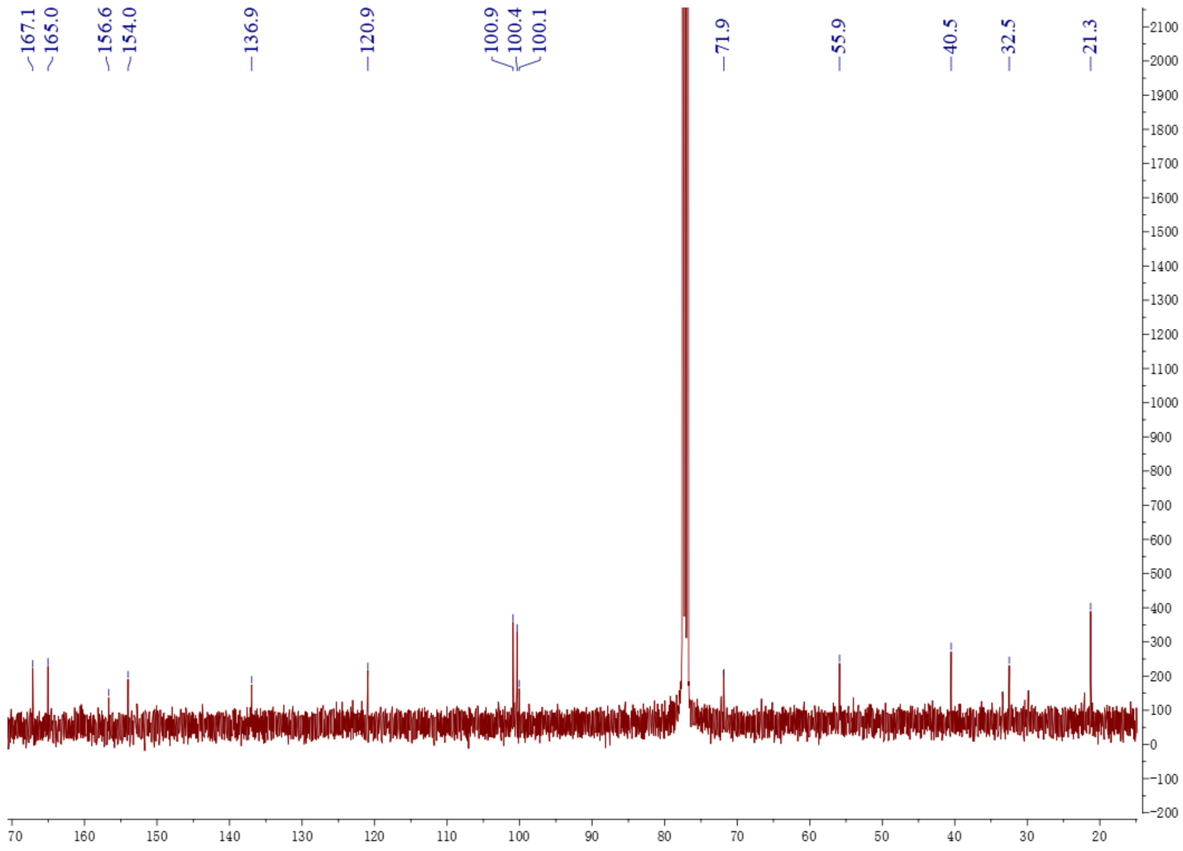

Figure 44. The ^{13}C NMR (100MHz) spectrum of compound **9** in $CDCl_3$-*d*.

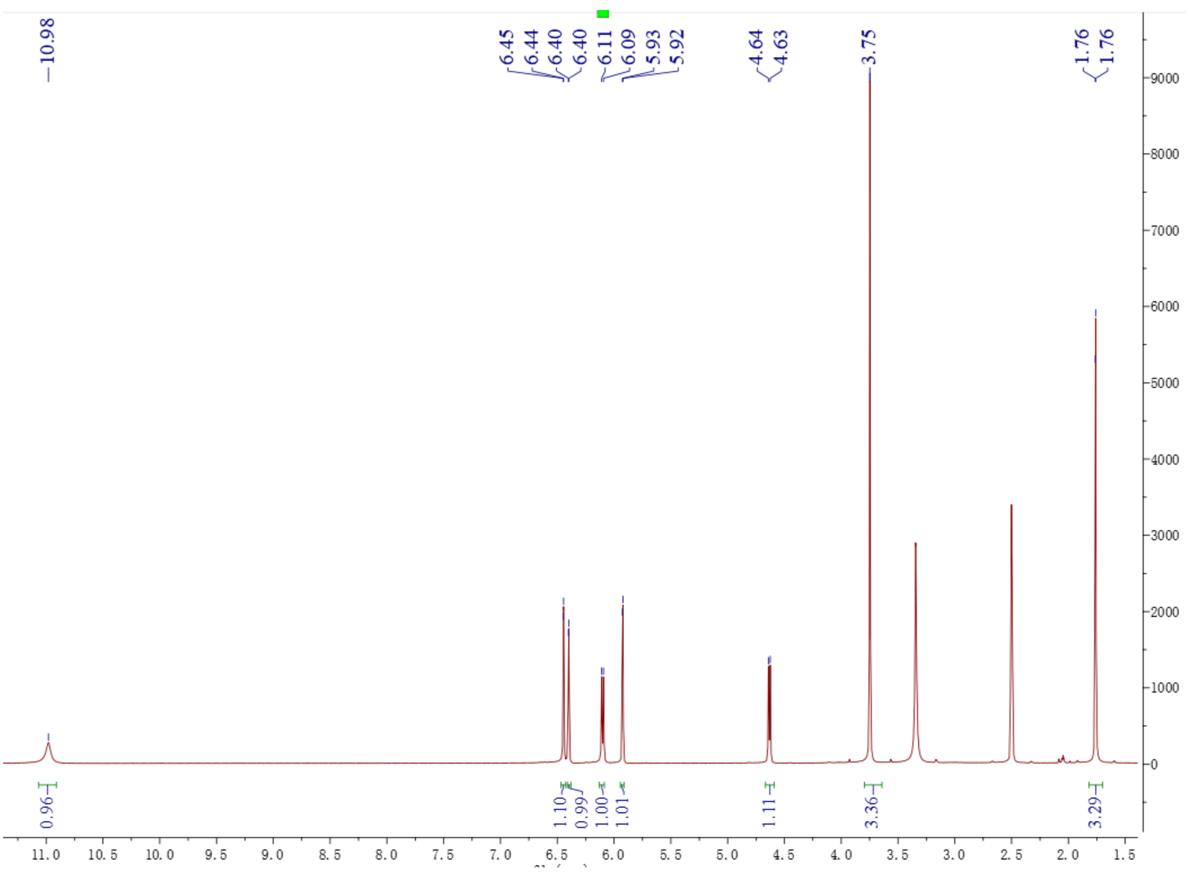

Figure 45. The ^{1}H NMR (400MHz) spectrum of compound **10** in DMSO-d_6.

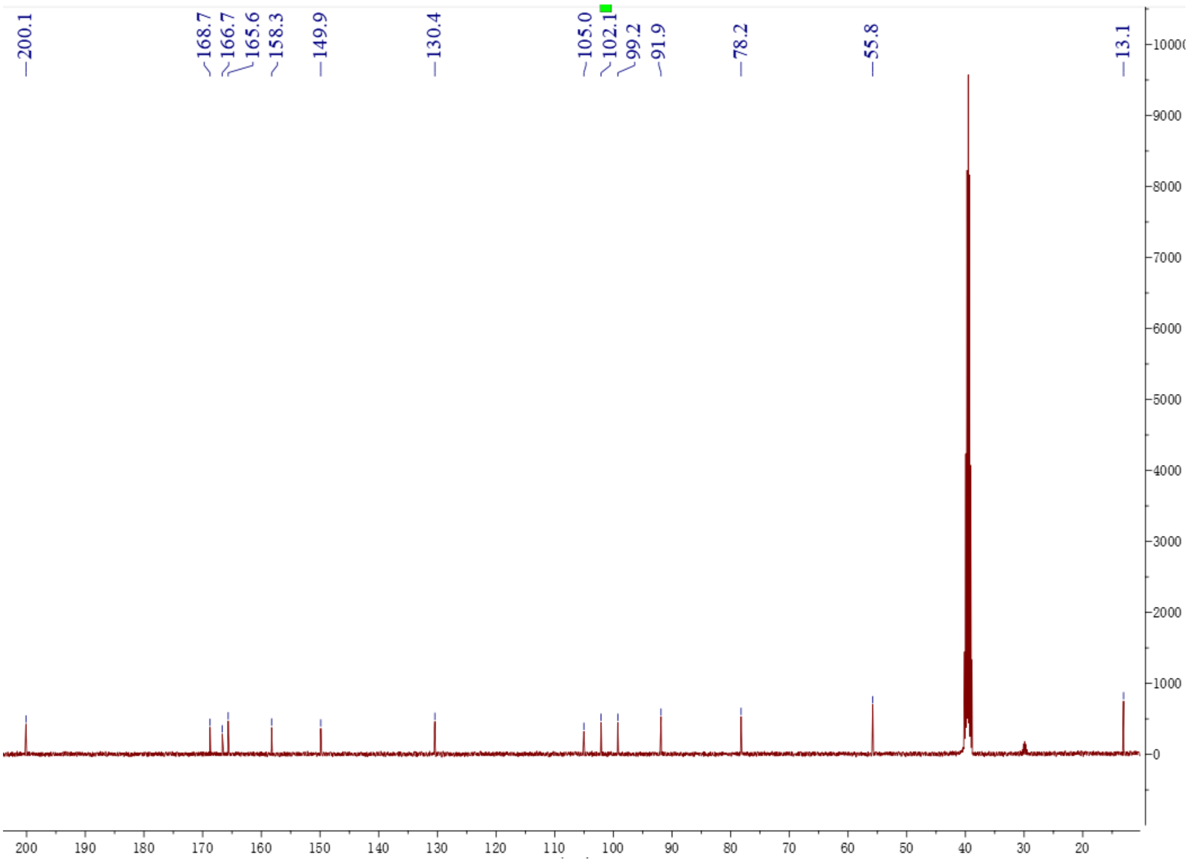

Figure 46. The ^{13}C NMR (100MHz) spectrum of compound **10** in DMSO-d_6.

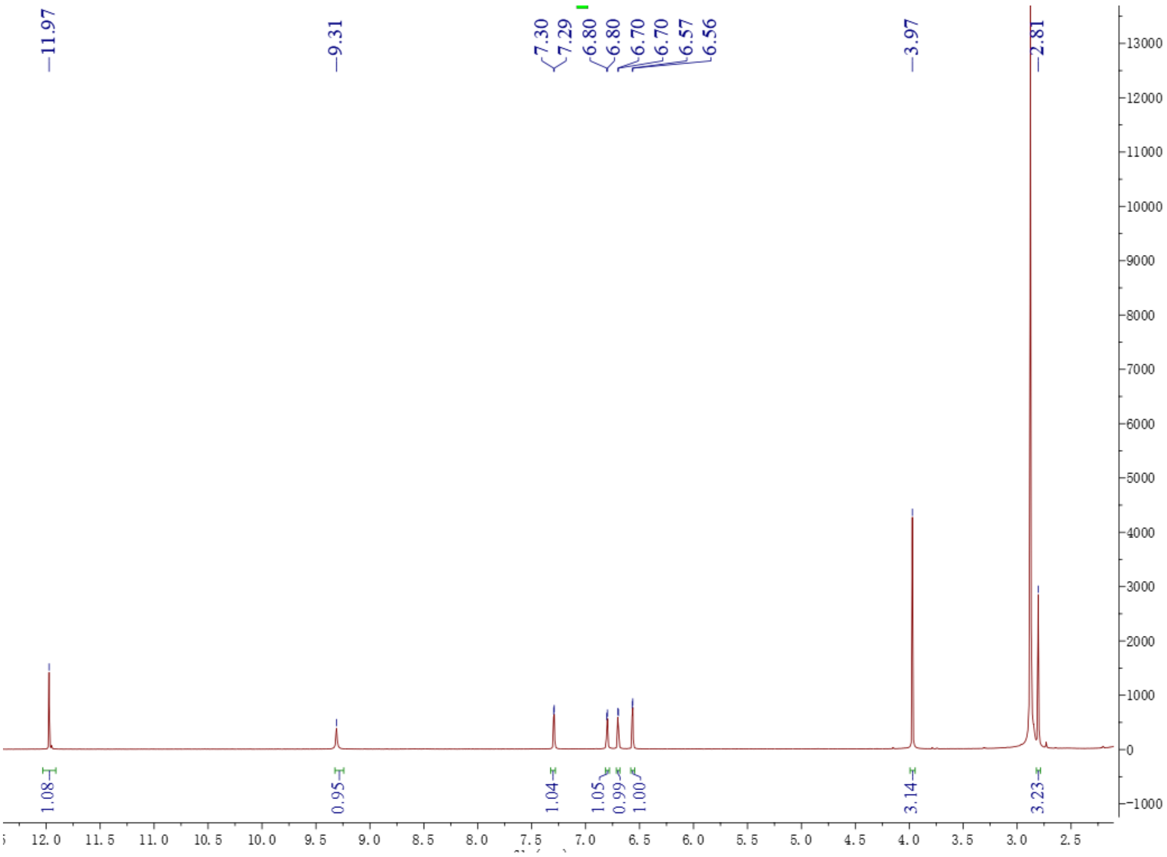

Figure 47. The ^{1}H NMR (400MHz) spectrum of compound **11** in acetone-d_6.

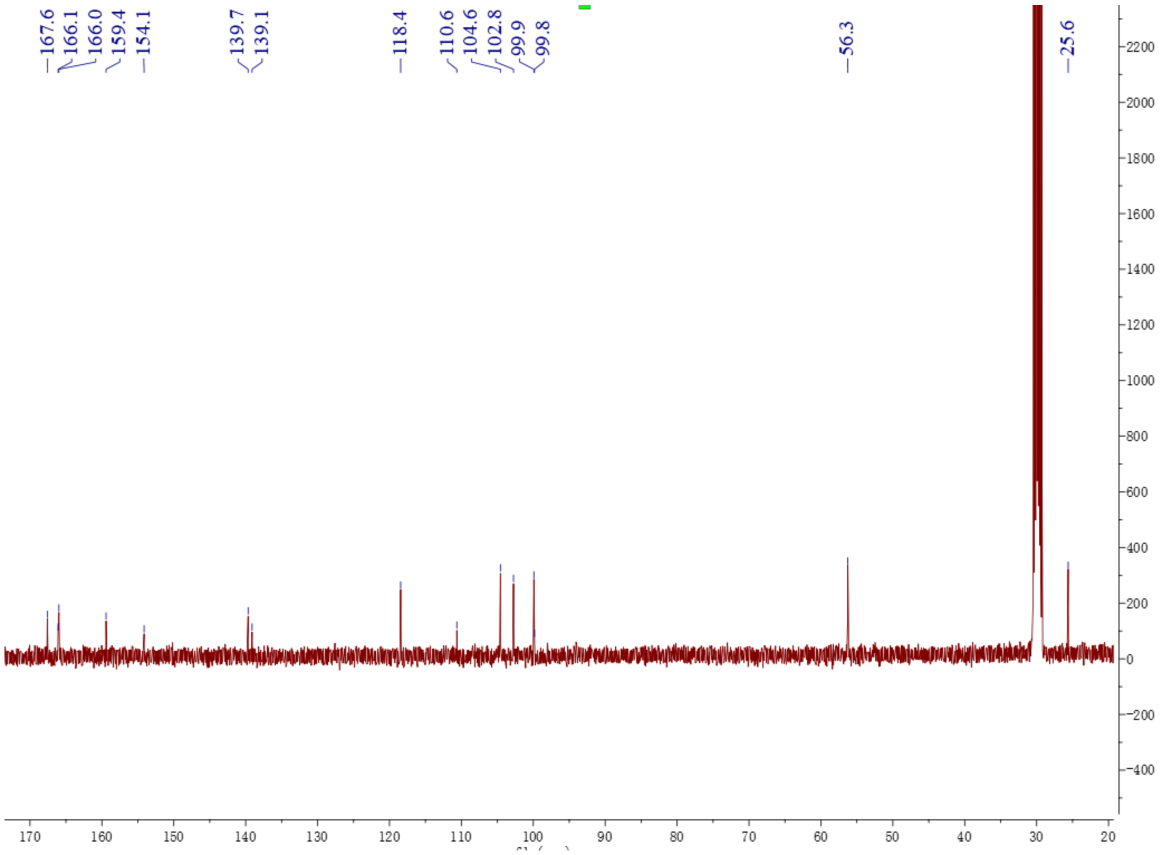

Figure 48. The ^{13}C NMR (100MHz) spectrum of compound **11** in acetone-d_6.

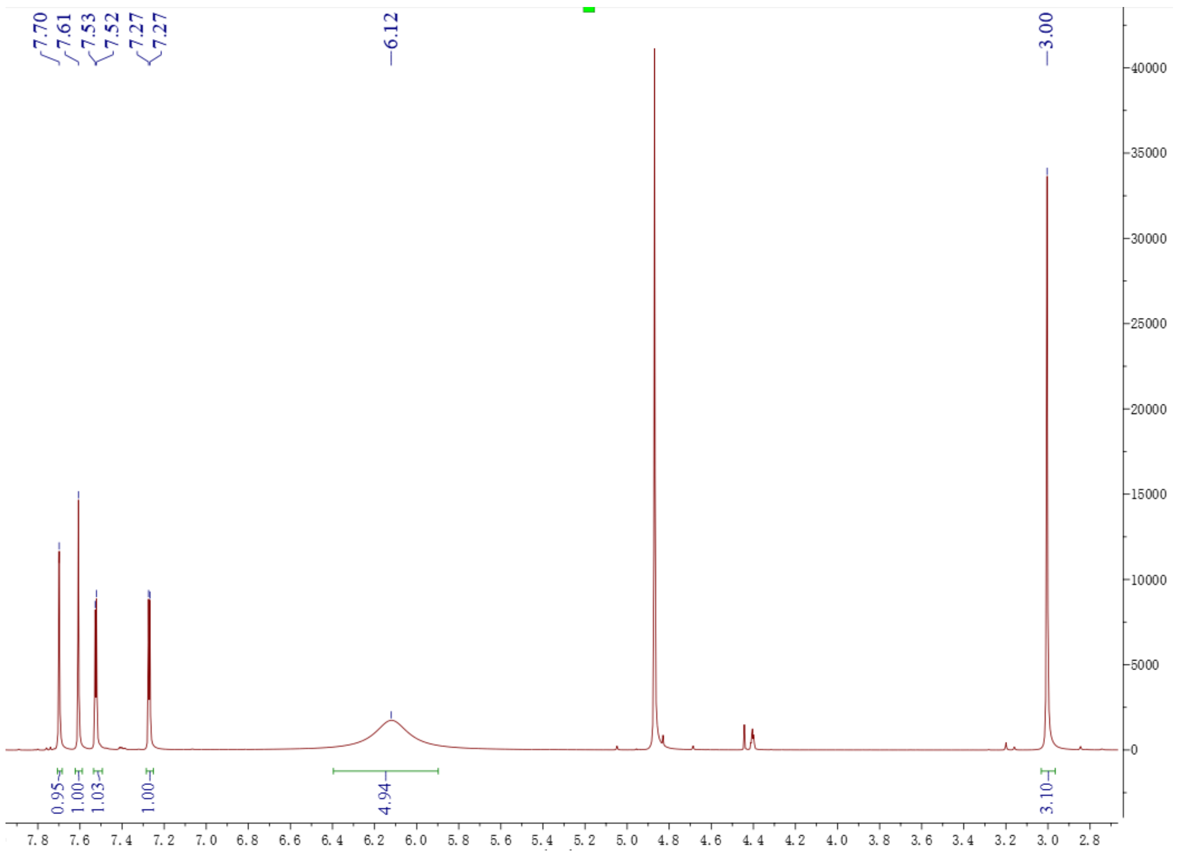

Figure 49. The ^{1}H NMR (400MHz) spectrum of compound **13** in MeOH-d_4.

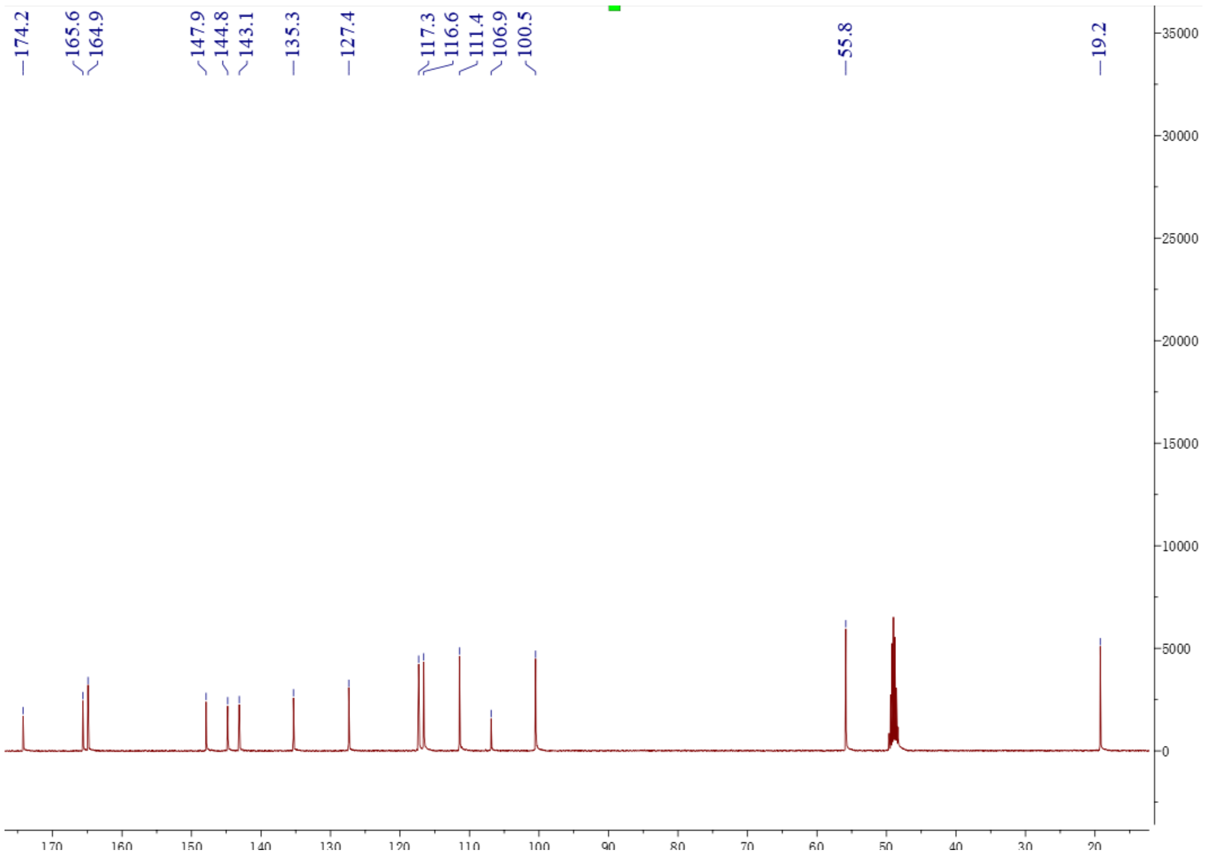

Figure 50. The ^{13}C NMR (100MHz) spectrum of compound **13** in MeOH-d_4.

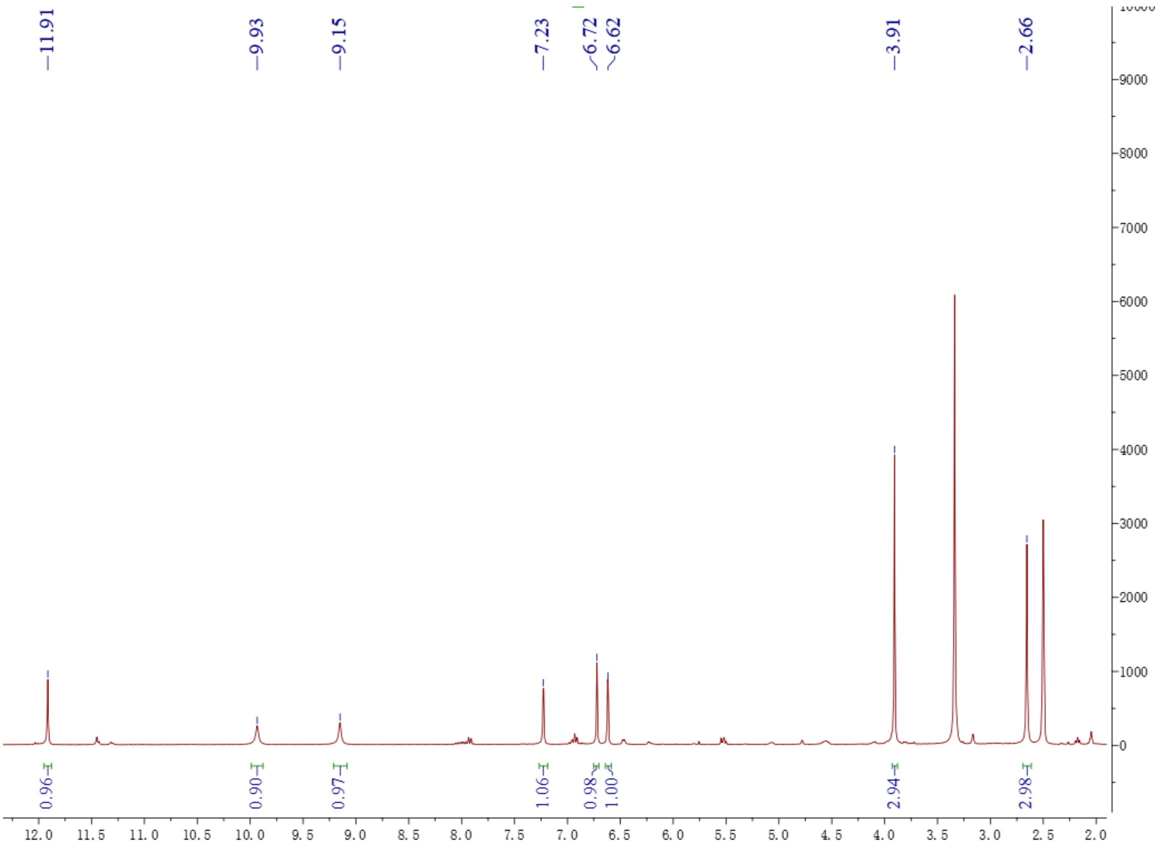

Figure 51. The ^{1}H NMR (400MHz) spectrum of compound **14** in DMSO-d_6.

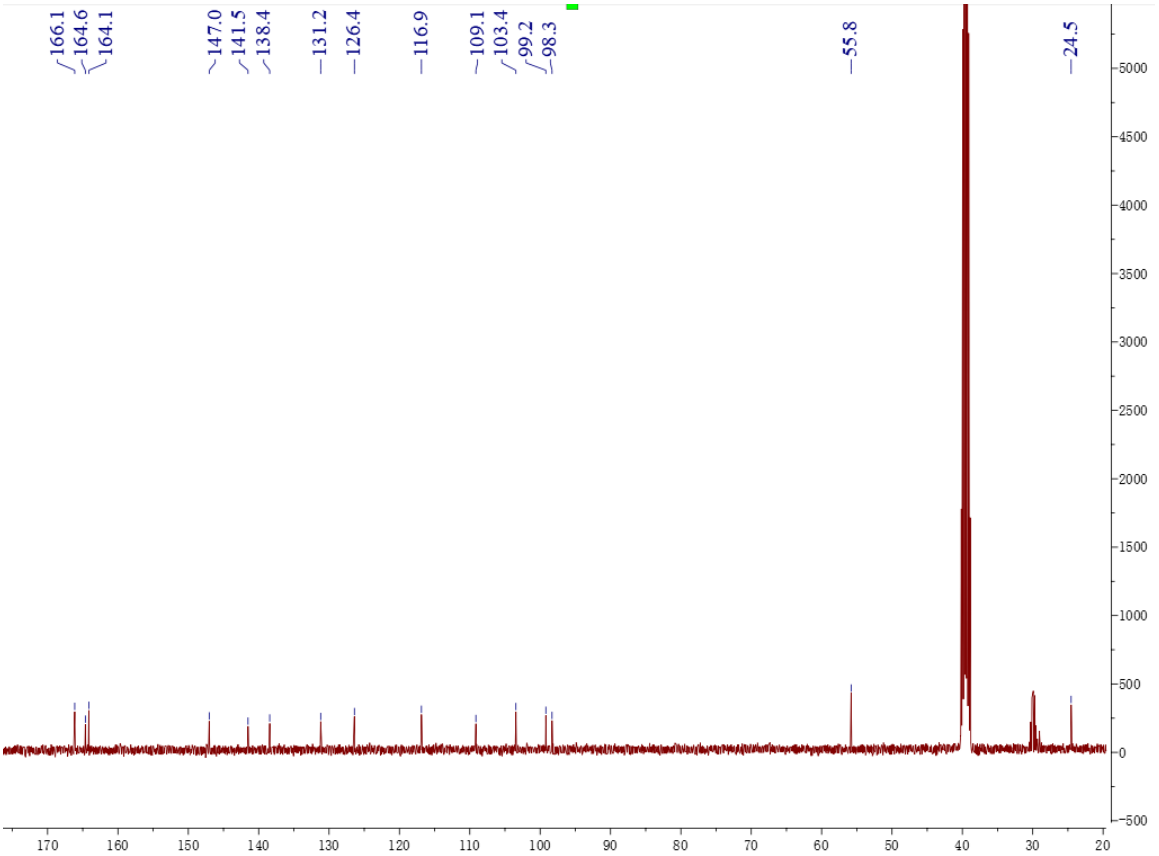

Figure 52. The ^{13}C NMR (100MHz) spectrum of compound **14** in DMSO-d_6.

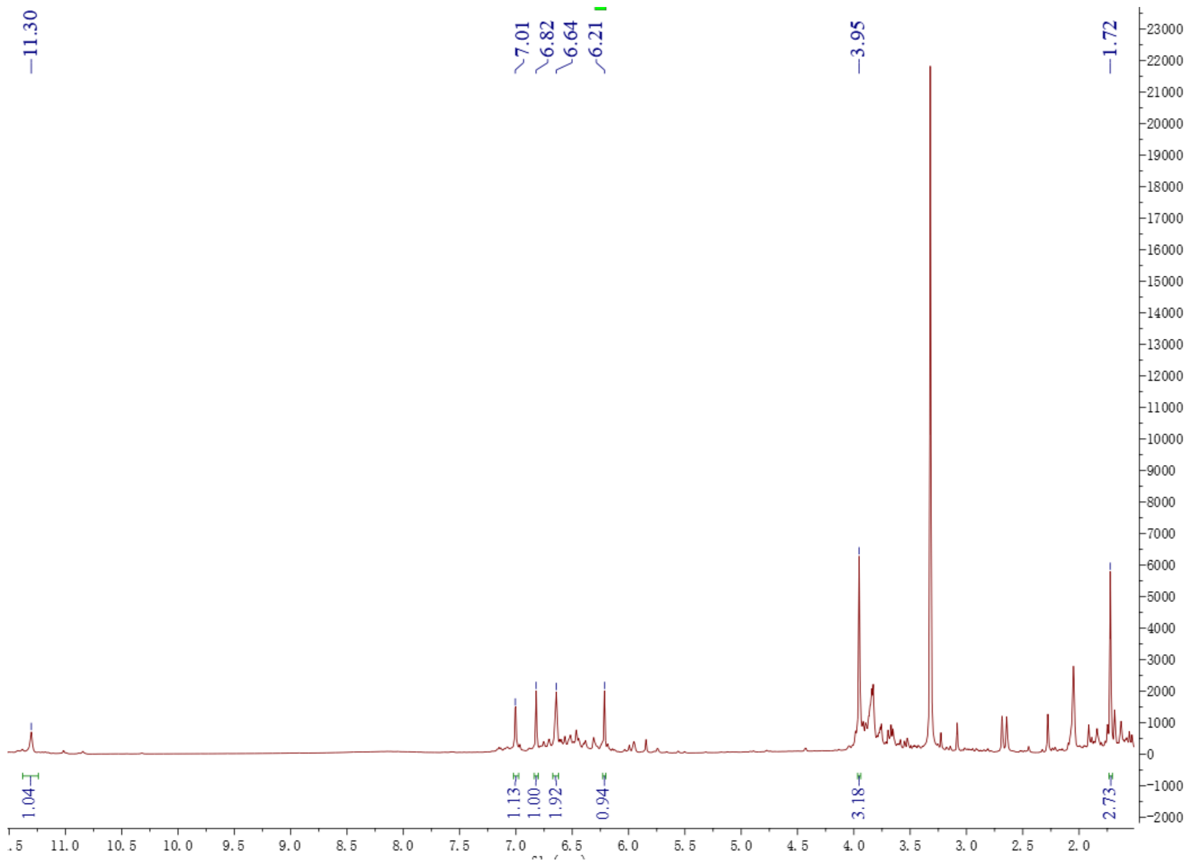

Figure 53. The ^{1}H NMR (400MHz) spectrum of compound **16** in acetone-d_6.

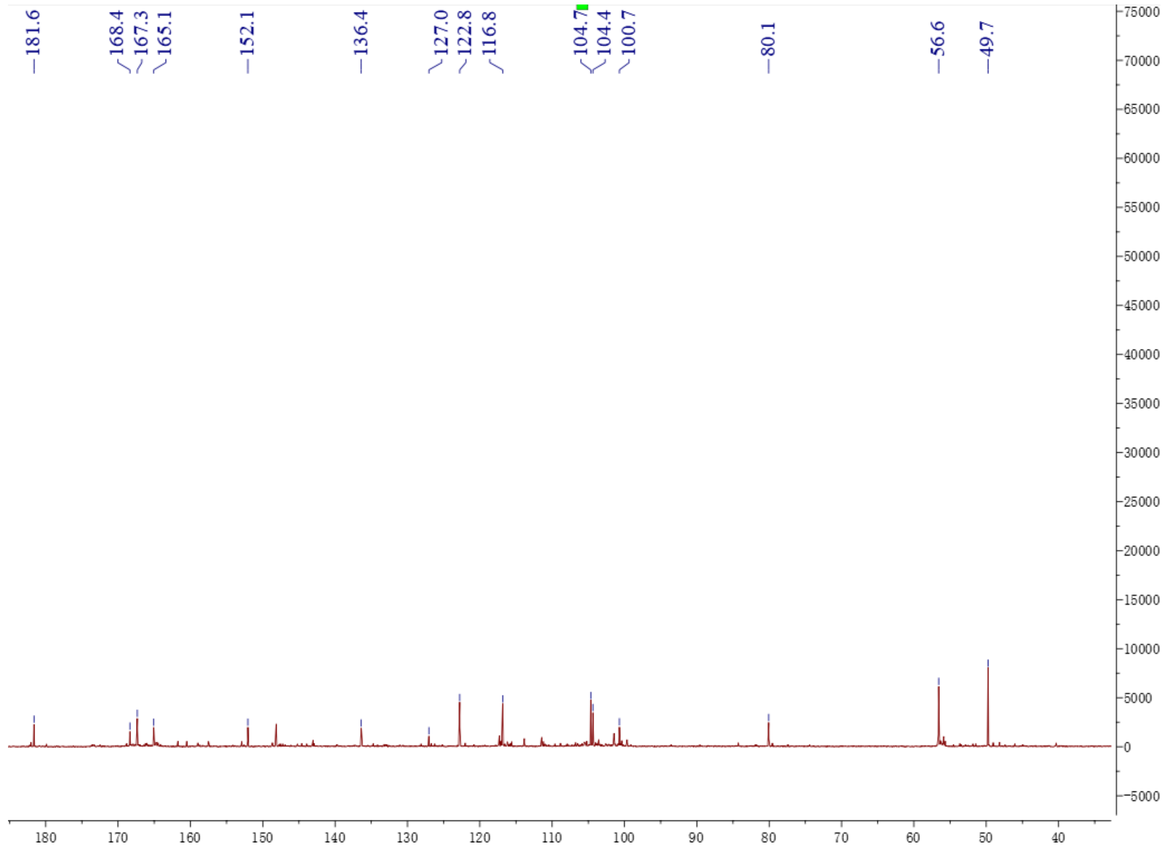

Figure 54. The ^{13}C NMR (100MHz) spectrum of compound **16** in acetone-d_6.

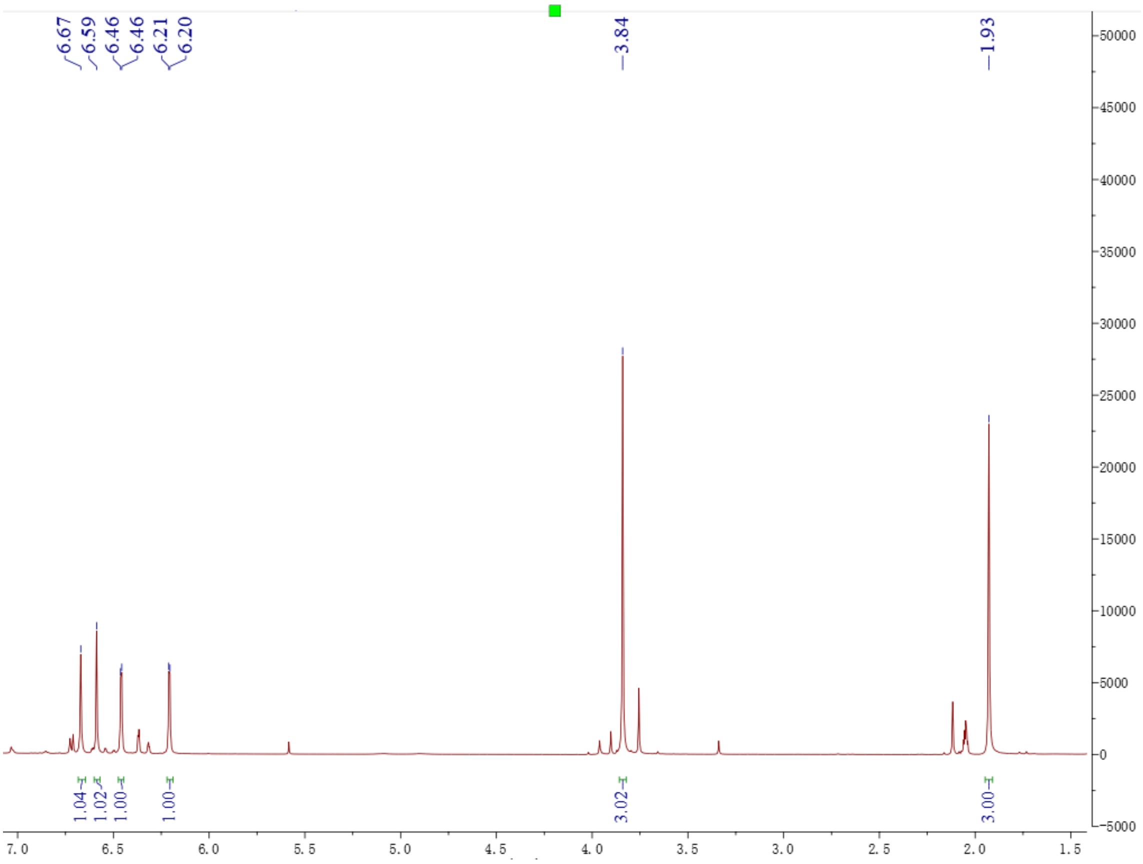

Figure 55. The ^{1}H NMR (400MHz) spectrum of compound **17** in acetone-d_6.

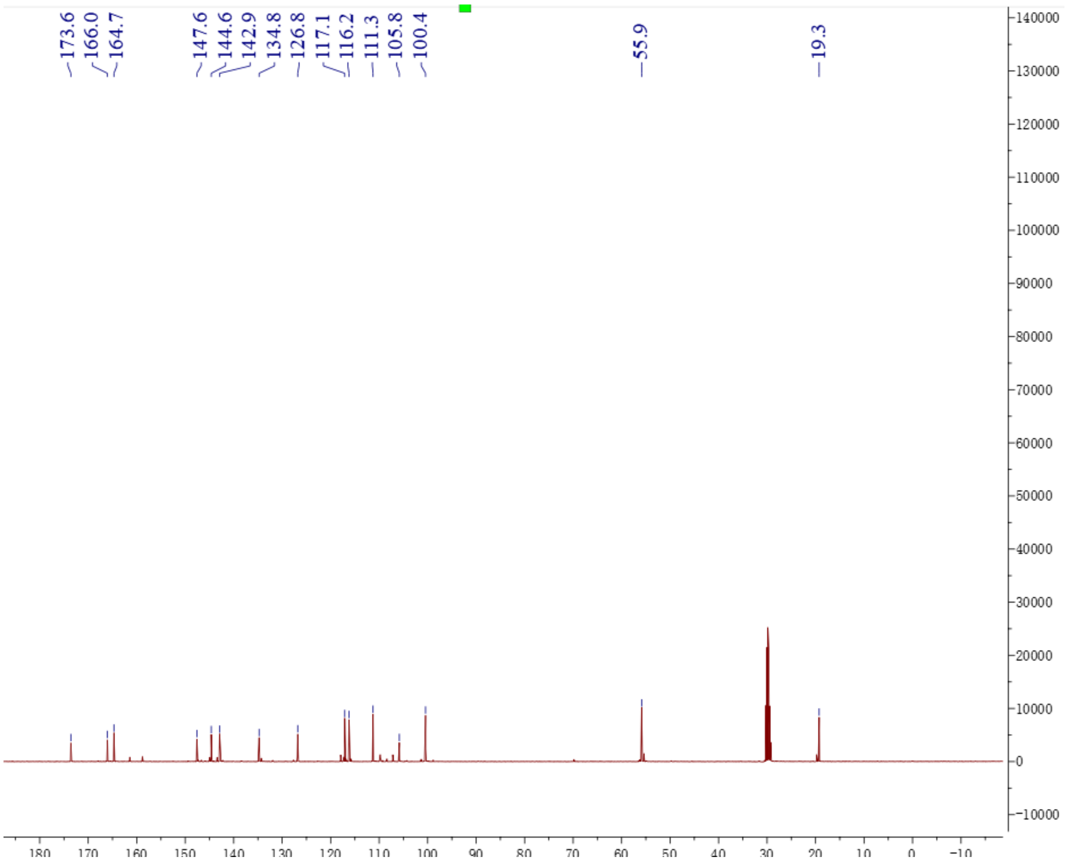

Figure 56. The ^{13}C NMR (100MHz) spectrum of compound **17** in acetone-d_6.

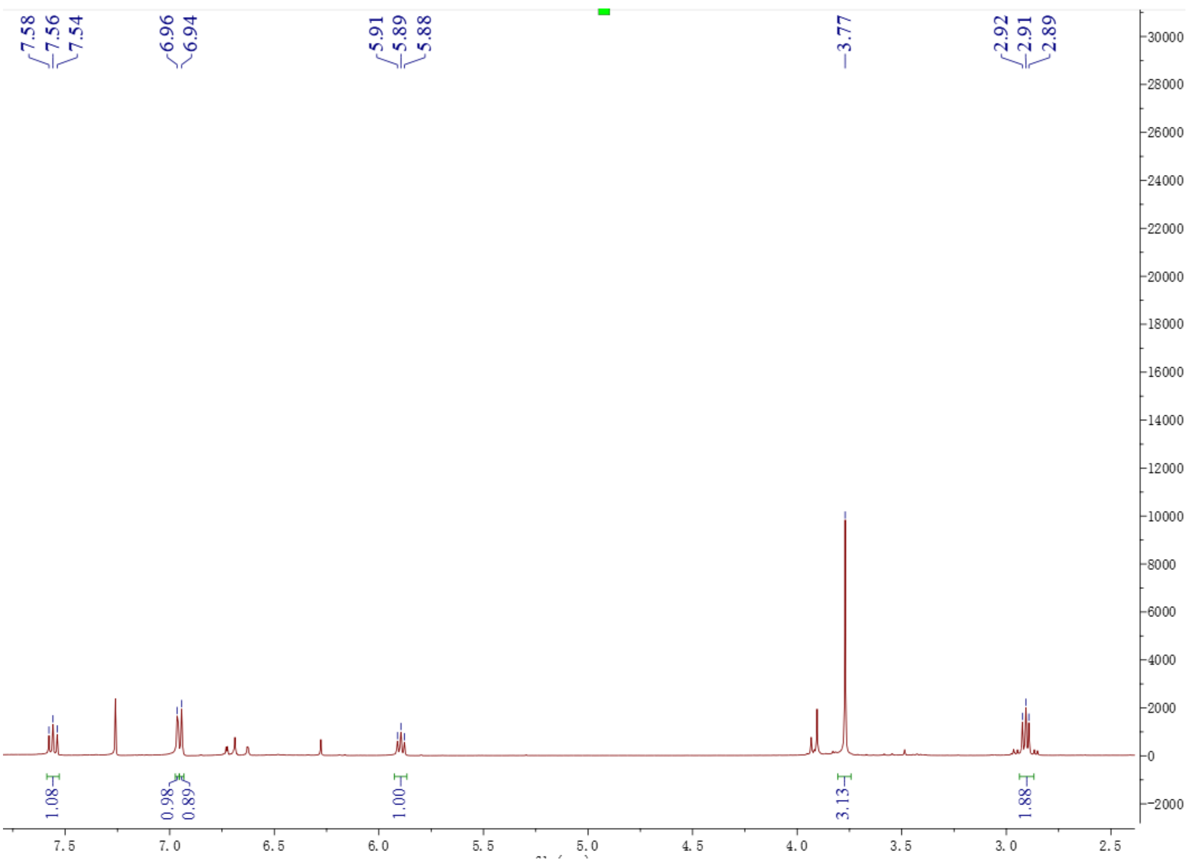

Figure 57. The ^{1}H NMR (400MHz) spectrum of compound **18** in $CDCl_3$-*d*.

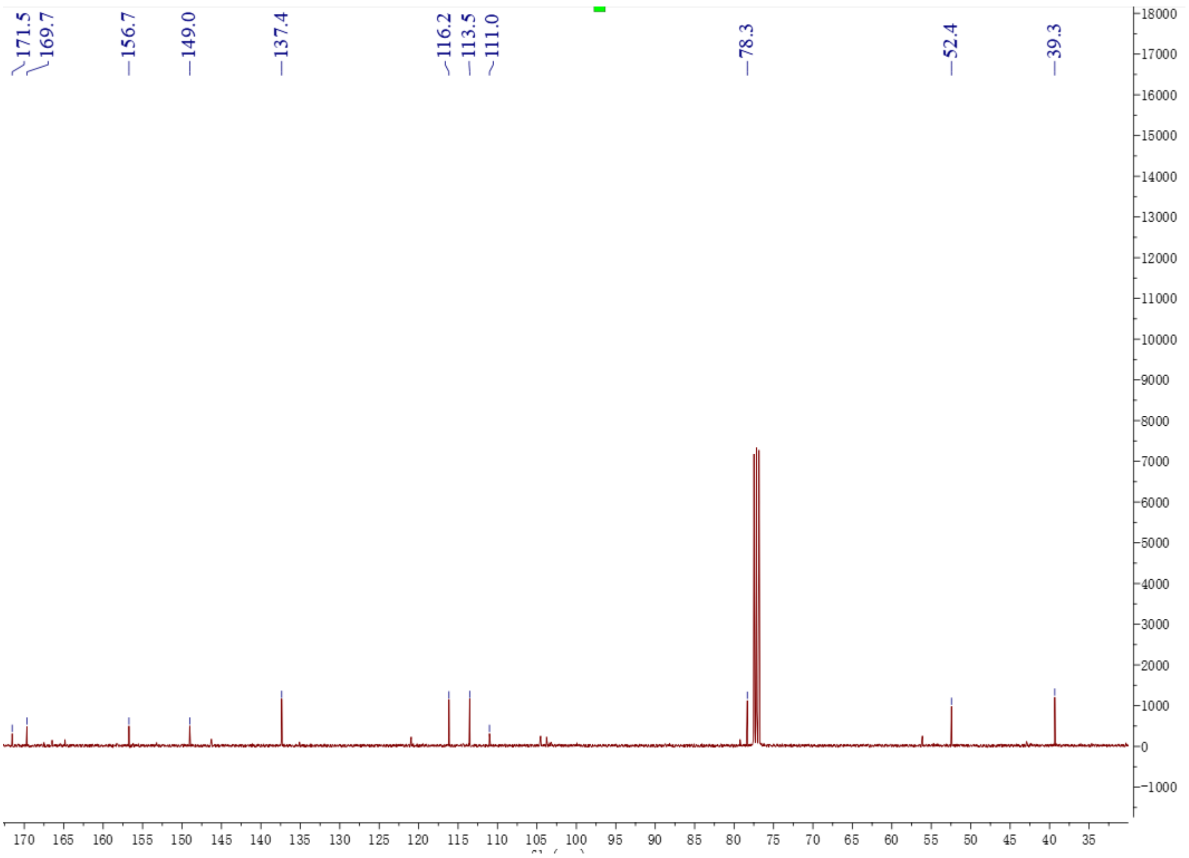

Figure 58. The ^{13}C NMR (100MHz) spectrum of compound **18** in $CDCl_3$-*d*.

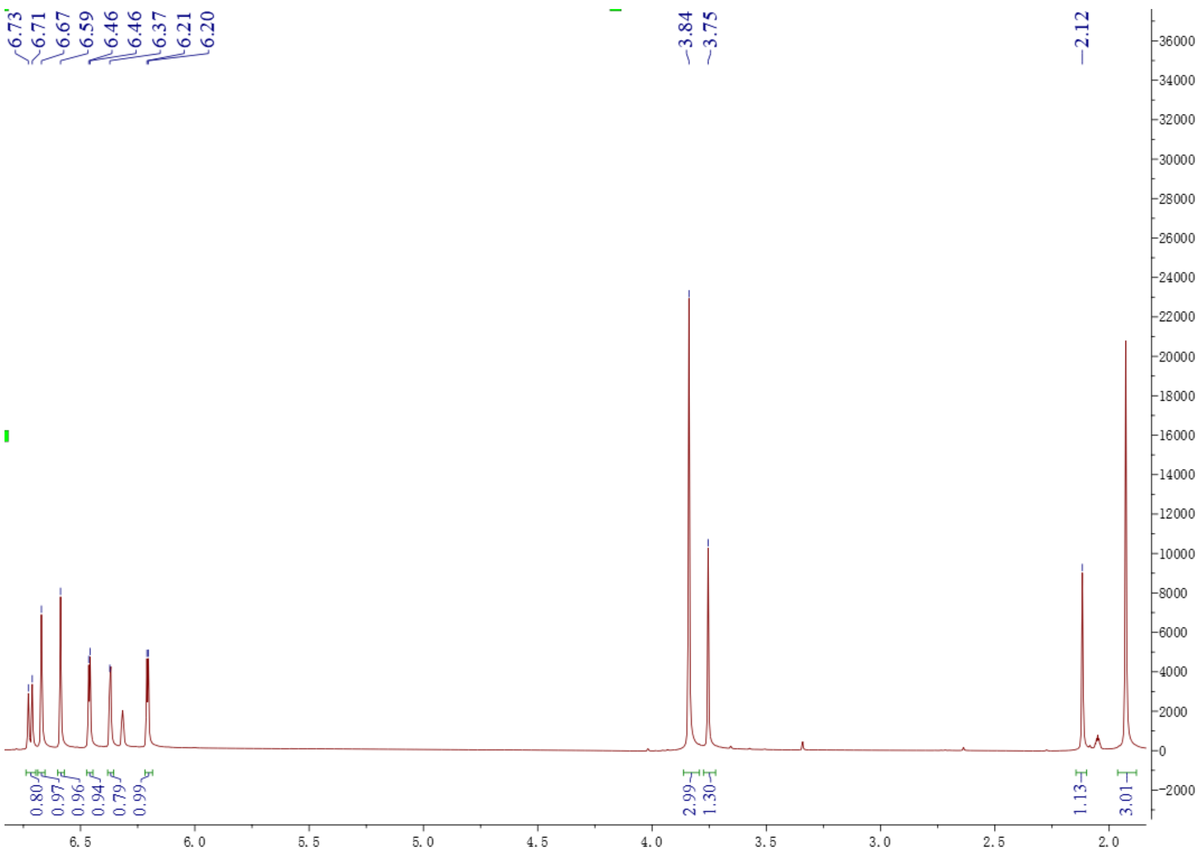

Figure 59. The ^{1}H NMR (400MHz) spectrum of compound **19** in acetone-d_6.

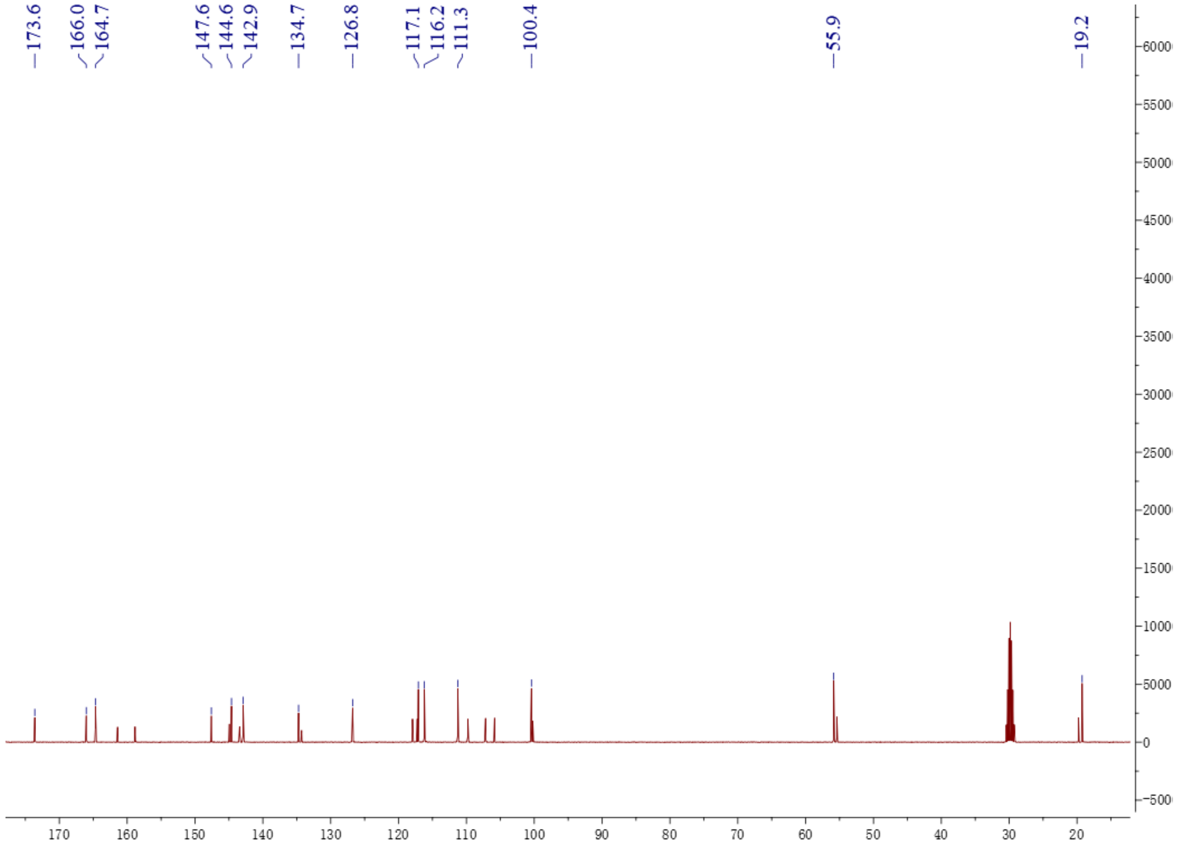

Figure 60. The ^{13}C NMR (100MHz) spectrum of compound **19** in acetone-d_6.

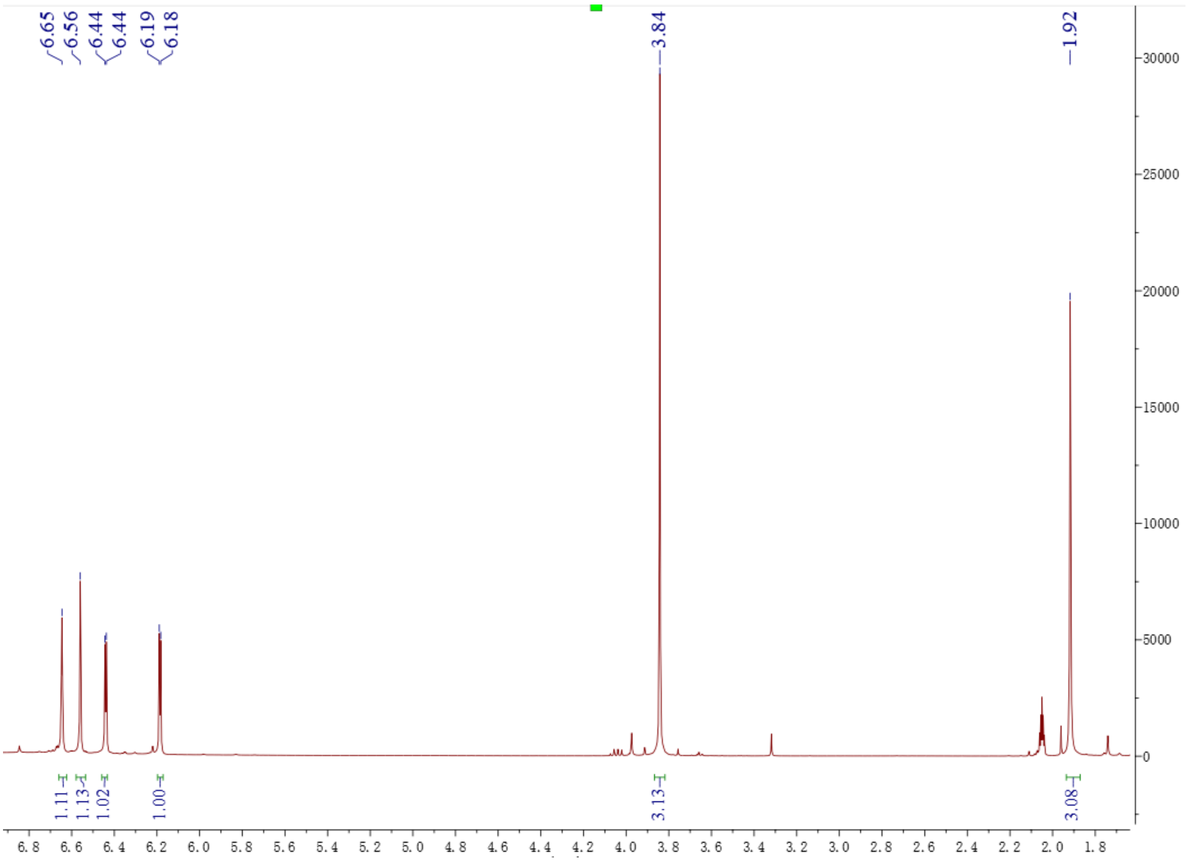

Figure 61. The ^{1}H NMR (400MHz) spectrum of compound **20** in acetone-d_6.

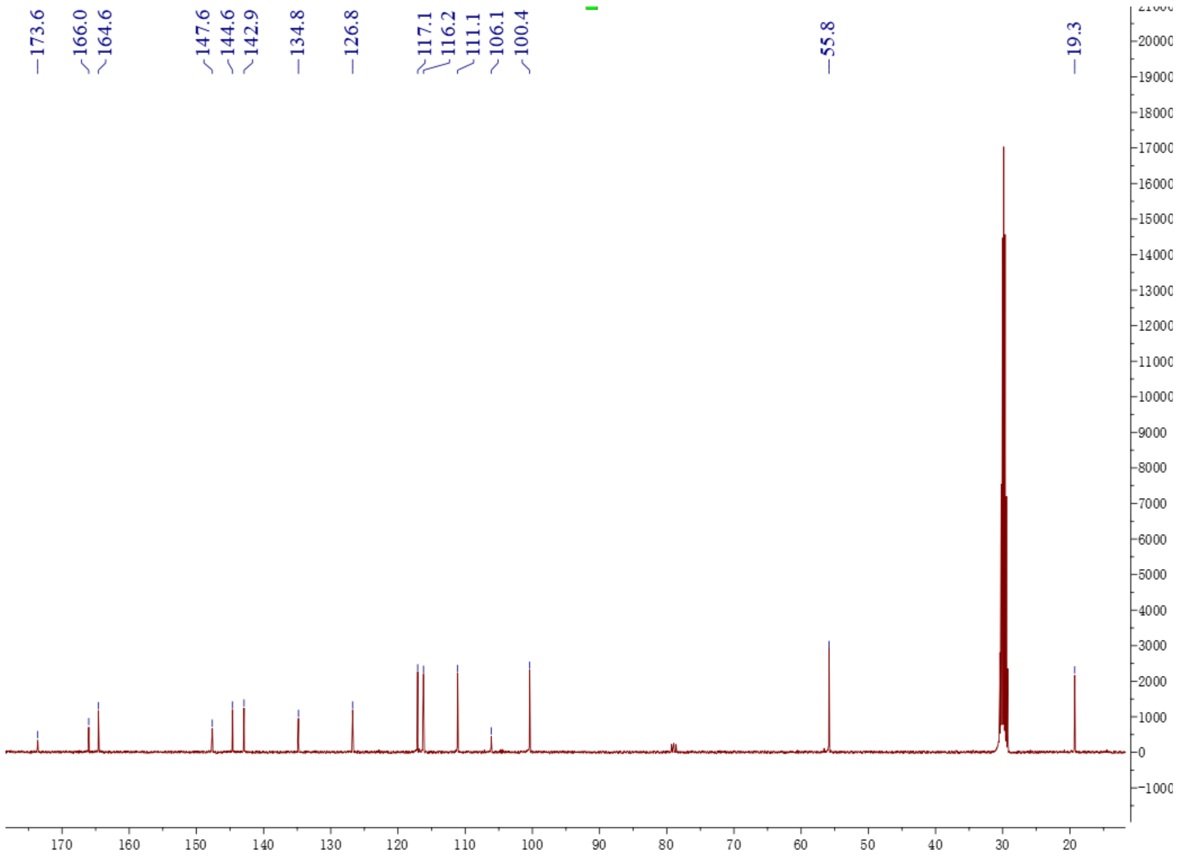

Figure 62. The ^{13}C NMR (100MHz) spectrum of compound **20** in acetone-d_6.

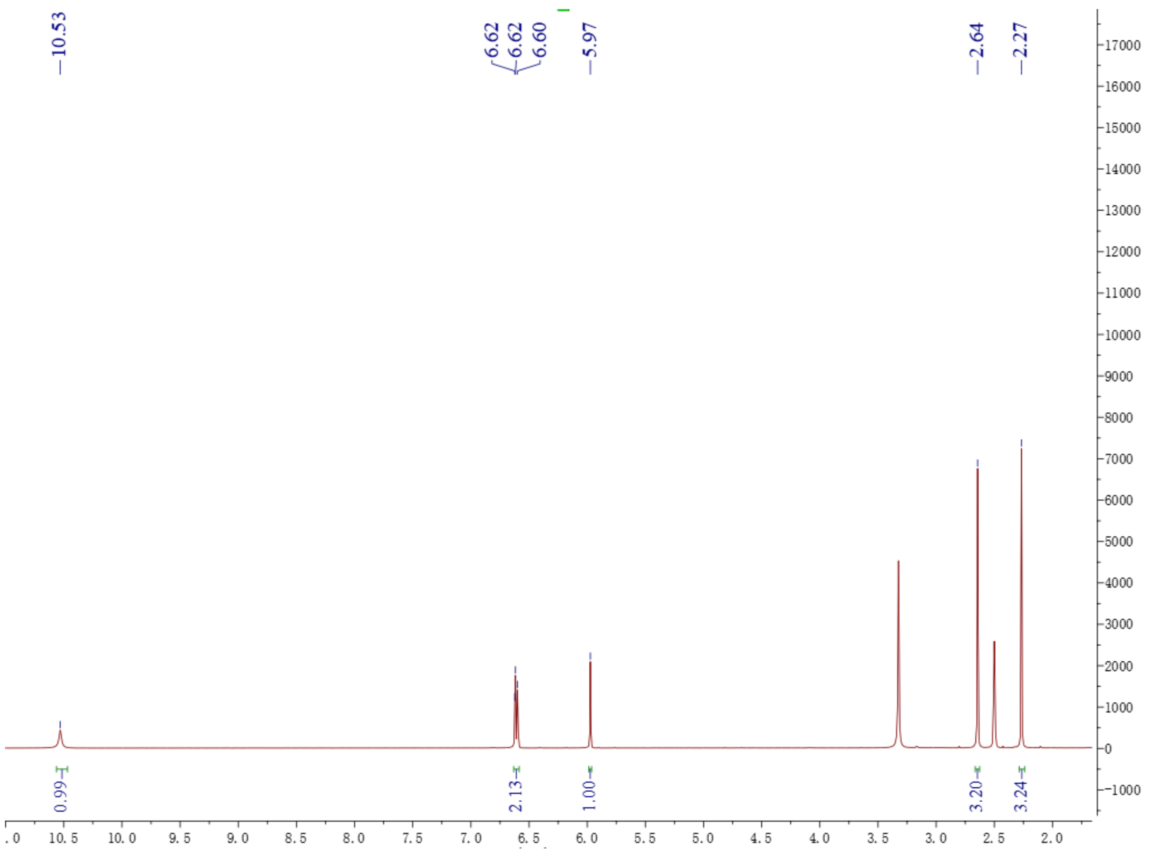

Figure 63. The ^{1}H NMR (400MHz) spectrum of compound **21** in DMSO-d_6.

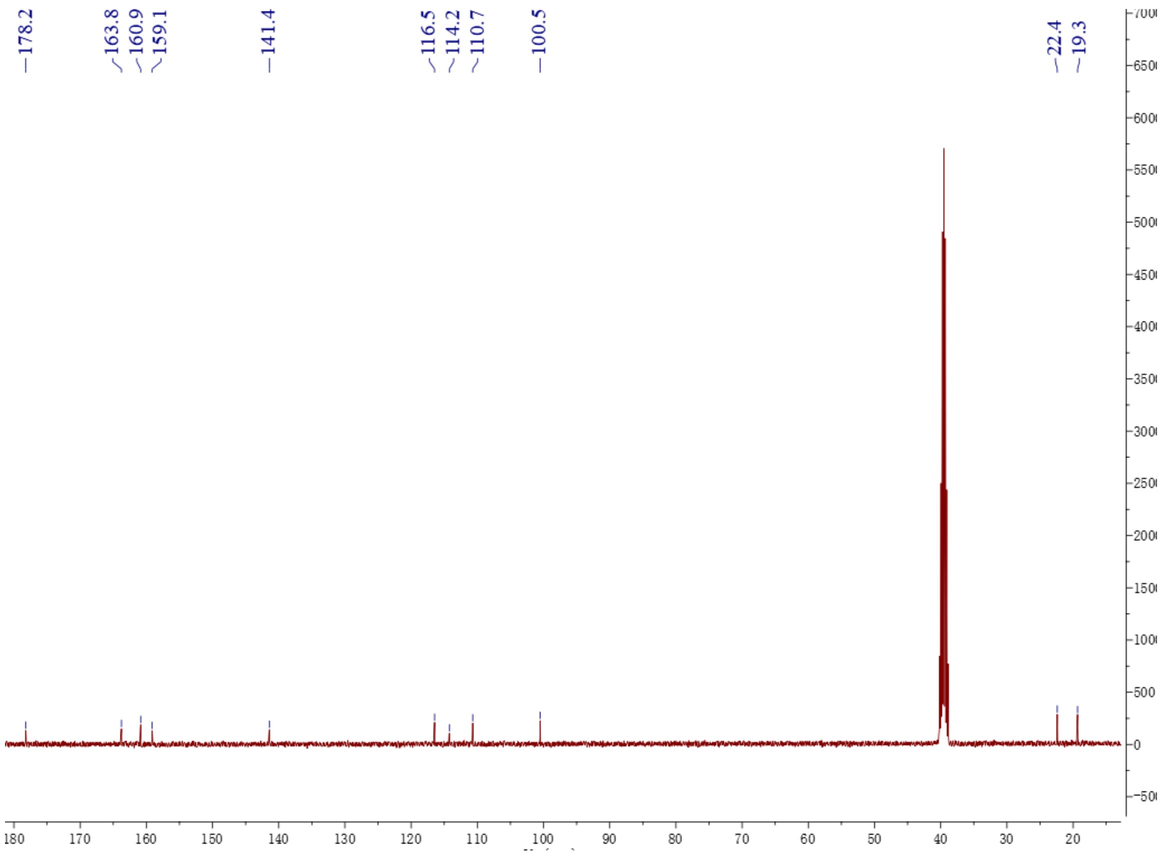

Figure 64. The ^{13}C NMR (100MHz) spectrum of compound **21** in DMSO-d_6.

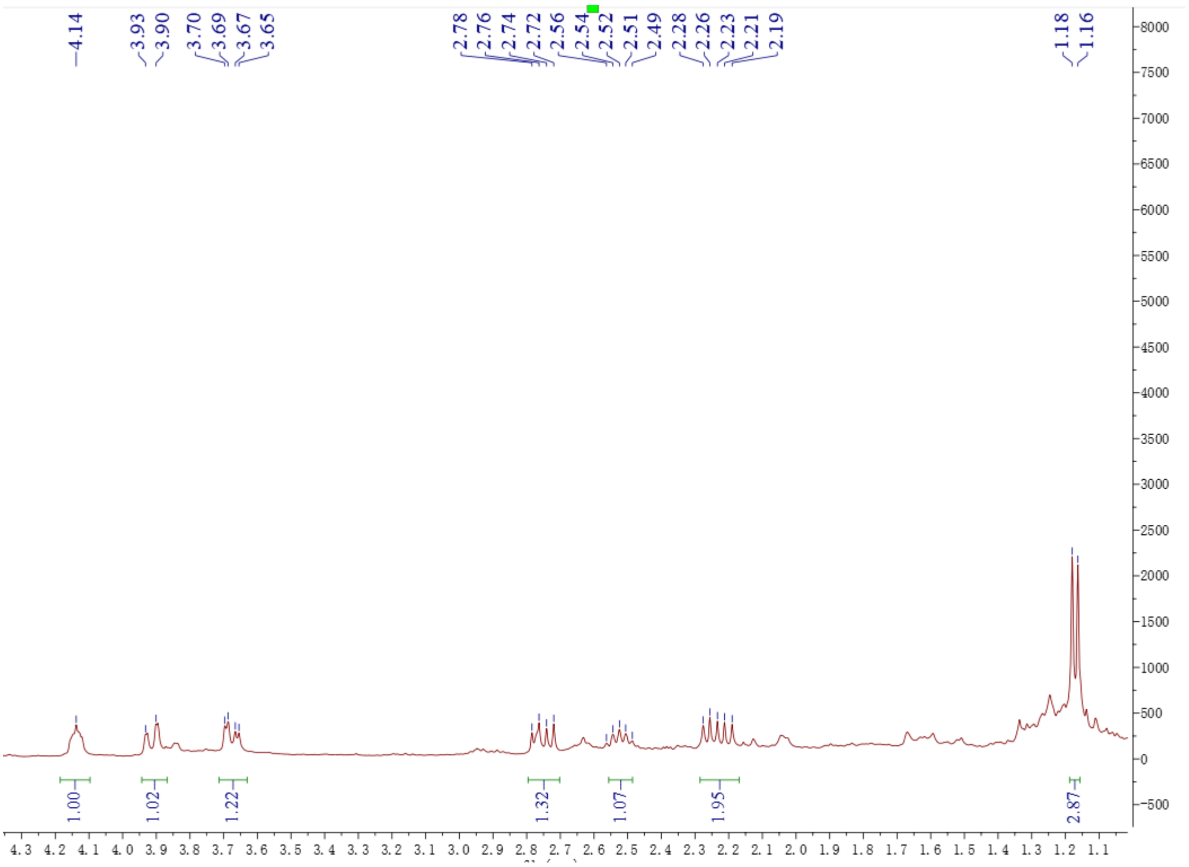

Figure 65. The ^{1}H NMR (400MHz) spectrum of compound **22** in $CDCl_3$-*d*.

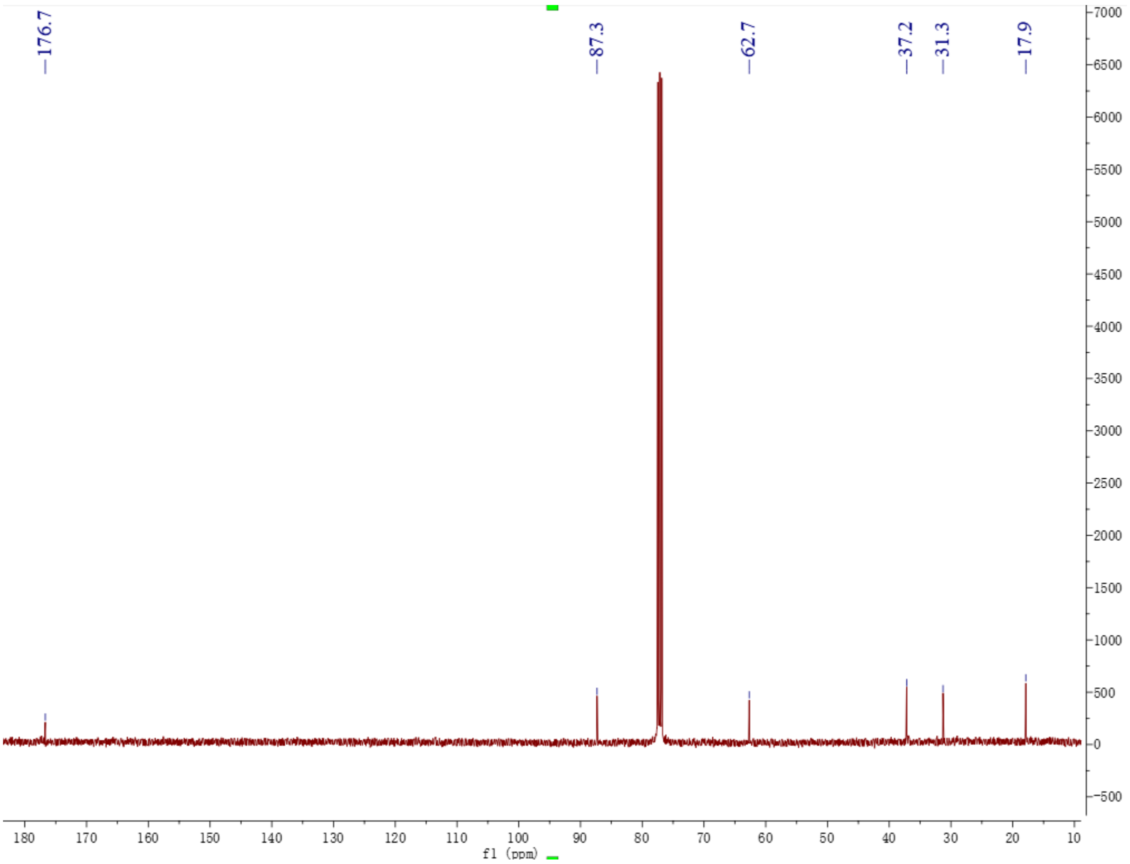

Figure 66. The ^{13}C NMR (100MHz) spectrum of compound **22** in $CDCl_3$-*d*.

Experimental Section

Calculation of ECD Spectra

Molecular Merck force field (MMFF) and DFT/TD-DFT calculations were carried out with Spartan' 14 software (Wavefunction Inc., Irvine, CA, USA) and Gaussian 09 program, respectively[1]. Conformers within 10 kcal/mol energy window were generated and optimized using DFT calculations at B3LYP/6-31G(d) level. Conformers with Bolzmann distribution over 1% were chosen for ECD calculations in methanol at B3lYP/6-311+g(2d,p) level. The IEF-PCM solvent model for MeOH was used. ECD spectra were generated using the program SpecDis 3.0 (University of Würzburg, Würzburg, Germany) and OriginPro 8.5 (OriginLab, Ltd., Northampton, MA, USA) from dipole-length rotational strengths by applying Gaussian band shapes with sigma = 0.30 ev. All calculations were performed by Tianhe-2 in National Super Computer Center in Guangzhou.

Table S1. Energy Analysis for the Conformers of (4*S*,5*S*,6*S*,10*R*)-3.

compound	Conformation	G (Hartree)	G (Kcal/mol)	ΔG (Kcal/mol)	Boltzmann Dist (%)
(4*S*,5*S*,6*S*,10*R*)-**3**	**3-1**	-735.4527047	-461503.5362	0	27.76
(4*S*,5*S*,6*S*,10*R*)-**3**	**3-2**	-735.4525708	-461503.4522	0.084003438	24.08
(4*S*,5*S*,6*S*,10*R*)-**3**	**3-3**	-735.4525708	-461503.4522	0.084003438	24.08
(4*S*,5*S*,6*S*,10*R*)-**3**	**3-4**	-735.4525708	-461503.4522	0.084003438	24.08

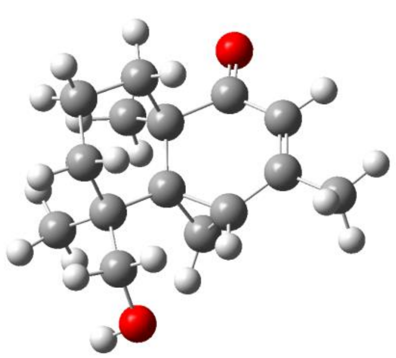

3-1

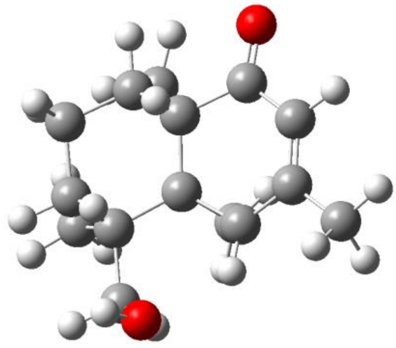

3-2

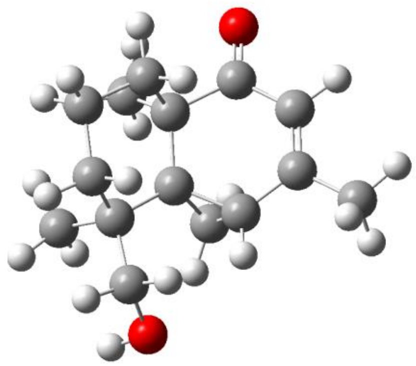

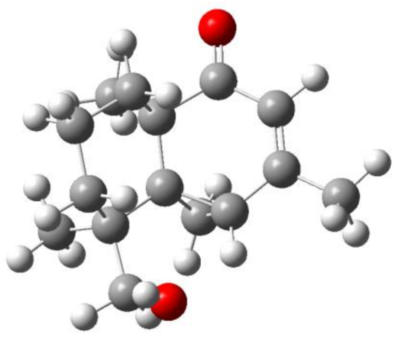

3-3 **3-4**

Figure S67. B3LYP/6-31G(d) optimized low-energy conformers of (4*S*,5*S*,6*S*,10*R*)**-3.**

Table S2. Energy Analysis for the Conformers of (4*S*,5*S*,6*S*,10*R*)-3.

compound	Conformation	G (Hartree)	G (Kcal/mol)	ΔG (Kcal/mol)	Boltzmann Dist (%)
**(**5*S*,6*R*,10*R*)**-4**	**4-1**	-735.4515258	-461502.7964	0	0.532700266
**(**5*S*,6*R*,10*R*)**-4**	**4-2**	-735.4502537	-461501.9982	0.798237225	0.138352351
**(**5*S*,6*R*,10*R*)**-4**	**4-3**	-735.4500343	-461501.8605	0.935910293	0.109649128
**(**5*S*,6*R*,10*R*)**-4**	**4-4**	-735.4500343	-461501.8605	0.935910293	0.109649128
**(**5*S*,6*R*,10*R*)**-4**	**4-5**	-735.4500343	-461501.8605	0.935910293	0.109649128

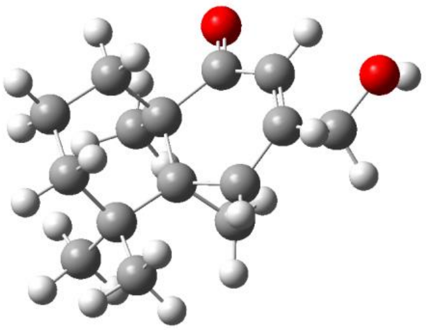

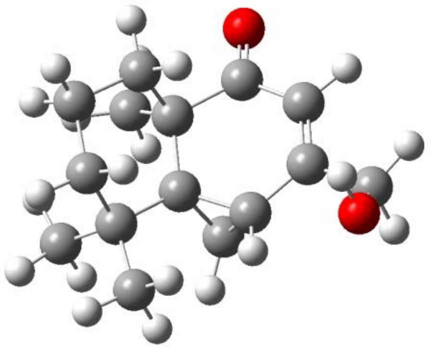

4-1 **4-2**

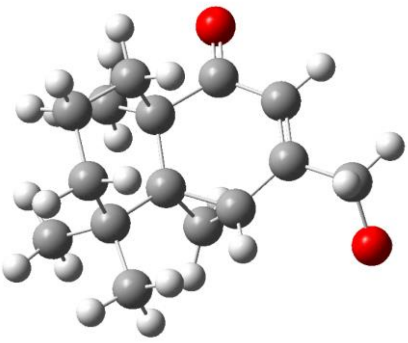

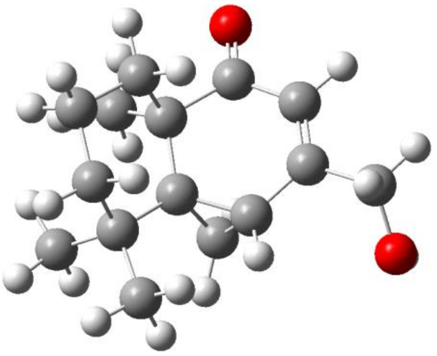

4-3 **4-4**

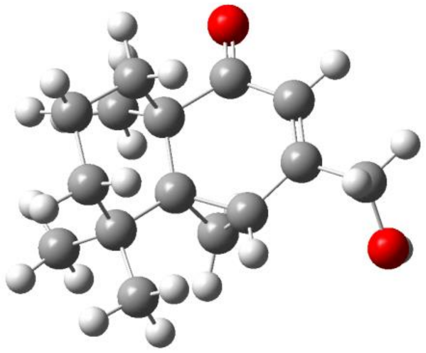

4-5

Figure S68. B3LYP/6-31G(d) optimized low-energy conformers of (5*S*,6*R*,10*R*)**-4.**

NMR data of known compounds 5-22.

Compound 5: ^{1}H NMR (400 MHz, acetone-d_6) δ_H 7.59 (t, *J* = 7.7 Hz, 1H), 7.11 (d, *J* = 7.6 Hz, 1H), 6.94 (d, *J* = 8.2 Hz, 1H), 5.85 (dd, *J* = 8.2, 4.6 Hz, 1H), 3.70 (s, 3H), 3.13 (dd, *J* = 16.7, 4.5 Hz, 1H), 2.85 (dd, *J* = 16.7, 8.2 Hz, 1H); ^{13}C NMR (100 MHz, acetone-d_6) δ_C 170.5, 170.3, 157.4, 151.3, 137.4, 116.7, 114.1, 112.3, 78.1, 52.1, 39.6.

Compound 6: ^{1}H NMR (400 MHz, acetone-d_6) δ_H 7.58 (s, 1H), 7.13 (d, *J* = 7.5 Hz, 2H), 6.94 (d, *J* = 8.2 Hz, 2H), 5.85 (s, 1H), 3.11 (dd, *J* = 16.8, 4.7 Hz, 3H), 2.85 (dd, *J* = 16.8, 8.0 Hz, 3H); ^{13}C NMR (100 MHz, acetone-d_6) δ_C 171.0, 170.4, 157.1, 151.4, 137.4, 116.5, 114.2, 112.2, 78.23, 39.4.

Compound 7: ^{1}H NMR (400 MHz, acetone-d_6) δ_H 11.51 (s, 1H), 7.03 (d, *J* = 2.5 Hz, 1H), 6.57 (d, *J* = 2.4 Hz, 1H), 3.94 (s, 3H), 2.97 (s, 1H), 2.80 (s, 1H), 1.71 (s, 3H); ^{13}C NMR (100 MHz, DMSO) δ_C 178.2, 163.8, 160.9, 159.1, 141.4, 116.5, 114.2, 110.7, 100.5, 22.4, 19.3.

Compound 9: ^{1}H NMR (400 MHz, $CDCl_3$) δ_H 11.35 (s, 1H), 6.51 (d, *J* = 2.2 Hz, 1H), 6.38 (d, *J* = 2.4 Hz, 1H), 5.22 (m, 1H), 3.89 (s, 3H), 3.32 (d, *J* = 7.2 Hz, 1H), 2.18 (m, 2H), 1.28 (s, 1H); ^{13}C NMR (100 MHz, $CDCl_3$) δ_C 167.1, 165.1, 156.6, 154.0, 136.9, 120.9, 100.9, 100.4, 100.1, 71.9, 55.9, 40.5, 32.5, 21.3.

Compound 10: ^{1}H NMR (400 MHz, DMSO-d_6) δ_H 11.92 (m, 1H), 6.42 (dd, *J* = 18.6, 1.8 Hz, 2H), 6.10 (d, *J* = 6.5 Hz, 1H), 5.92 (d, *J* = 1.9 Hz, 1H), 4.63 (d, *J* = 6.4 Hz, 1H), 3.75 (s, 3H), 1.76 (d, *J* =

1.5 Hz, 3H); ^{13}C NMR (100 MHz, DMSO-d_6) δ_C 200.1, 168.7, 166.7, 165.6, 158.2, 149.9, 130.4, 105.0, 102.1, 99.3, 91.9, 78.2, 55.8, 13.1.

Compound 11: ^{1}H NMR (400 MHz, acetone-d_6) δ_H 11.97 (s, 1H), 9.26 (s, 1H), 7.30 (d, J = 2.3 Hz, 1H), 6.80 (m, 1H), 6.71 (d, J = 2.7 Hz, 1H), 6.57 (d, J = 2.2 Hz, 1H), 3.97 (s, 3H), 2.81 (s, 3H); ^{13}C NMR (100 MHz, acetone-d_6) δ_C 167.6, 166.0, 159.4, 154.1, 139.7, 139.1, 118.5, 110.7, 104.6, 102.8, 100.5, 99.9, 56.3, 25.6.

Compound 13: ^{1}H NMR (400 MHz, MeOH-d_4) δ_H 6.60 (s, 1H), 6.51 (s, 1H), 6.43 (d, J = 2.6 Hz, 1H), 6.18 (d, J = 2.6 Hz, 1H), 3.78 (s, 3H), 1.91 (s, 3H); ^{13}C NMR (100 MHz, MeOH-d_4) δ_C 174.2, 165.6, 164.9, 147.9, 144.8, 143.1, 135.3, 127.4, 117.3, 116.6, 111.4, 106.9, 100.5, 55.9, 19.3.

Compound 14: ^{1}H NMR (400 MHz, DMSO-d_6) δ_H 11.91 (s, 1H), 9.93 (s, 1H), 9.15 (s, 1H), 7.23 (d, J = 2.3 Hz, 1H), 6.72 (s, 1H), 6.62 (d, J = 2.1 Hz, 1H), 3.91 (s, 3H), 2.66 (s, 3H); ^{13}C NMR (100 MHz, DMSO-d_6) δ_C 166.1, 164.6, 164.1, 147.0, 141.5, 138.4, 131.2, 126.4, 116.9, 109.1, 103.4, 99.2, 98.3, 55.8, 24.5.

Compound 16: ^{1}H NMR (400 MHz, acetone-d_6) δ_H 11.30 (s, 1H), 7.00 (d, J = 2.2 Hz, 1H), 6.82 (d, J = 1.8 Hz, 1H), 6.65 (m, 1H), 6.21 (s, 1H), 3.95 (q, J = 2.4, 1.9 Hz, 3H), 1.72 (d, J = 1.9 Hz, 3H); ^{13}C NMR (100 MHz, acetone-d_6) δ_C 181.6, 168.4, 167.3, 165.1, 152.1, 148.1, 136.4, 122.8, 116.8, 104.7, 104.4, 100.7, 80.1, 56.6, 49.7.

Compound 17: ^{1}H NMR (400 MHz, acetone-d_6) δ_H 6.67 (s, 1H), 6.59 (s, 1H), 6.46 (d, J = 2.7 Hz, 2H), 6.21 (d, J = 2.6 Hz, 1H), 3.84 (s, 3H), 1.93 (s, 3H); ^{13}C NMR (100 MHz, acetone-d_6) δ_C 173.6, 166.0, 164.7, 147.6, 144.6, 142.9, 134.8, 126.8, 117.1, 116.2, 111.3, 105.8, 100.4, 55.9, 19.2.

Compound 18: ^{1}H NMR (400 MHz, $CDCl_3$) δ_H 7.56 (t, J = 7.9 Hz, 1H), 6.96 (m, 2H), 5.89 (t, J = 6.6 Hz, 1H), 3.77 (s, 3H), 2.91 (dd, J = 6.7, 5.7 Hz, 2H); ^{13}C NMR (100 MHz, $CDCl_3$) δ_C 171.5, 169.7, 156.7, 149.0, 137.4, 116.2, 113.5, 111.0, 78.3, 52.4, 39.3.

Compound 19: ^{1}H NMR (400 MHz, acetone-d_6) δ_H 6.72 (d, J = 6.8 Hz, 1H), 6.67 (s, 1H), 6.59 (s, 1H), 6.46 (dd, J = 2.6, 0.8 Hz, 1H), 6.37 (dd, J = 1.9, 0.9 Hz, 1H), 6.21 (dd, J = 2.6, 0.9 Hz, 1H), 3.84 (d, J = 0.9 Hz, 3H), 1.93 (s, 3H); ^{13}C NMR (100 MHz, acetone-d_6) δ_C 173.6, 166.0, 164.6, 147.6, 144.6, 142.9, 134.7, 126.8, 117.0, 116.2, 111.3, 100.4, 55.9, 19.3.

Compound 20: ^{1}H NMR (400 MHz, acetone-d_6) δ_H 6.65 (s, 1H), 6.56 (s, 1H), 6.44 (d, J = 2.6 Hz, 1H), 6.19 (d, J = 2.6 Hz, 1H), 3.84 (s, 3H), 1.92 (s, 3H); ^{13}C NMR (100 MHz, acetone-d_6) δ_C 173.6, 166.0, 164.6, 147.6, 144.6, 142.9, 134.8, 126.8, 117.0, 116.2, 111.1, 106.1, 100.4, 55.8, 19.3.

Compound 21: ^{1}H NMR (400 MHz, DMSO-d_6) δ_H 10.53 (s, 1H), 6.61 (m, 2H), 5.97 (s, 1H), 2.64 (s, 3H), 2.27 (s, 3H); ^{13}C NMR (100 MHz, DMSO) δ_C 178.2, 163.8, 160.9, 159.1, 141.4, 116.5, 114.2, 110.7, 100.5, 22.4, 19.3.

Compound 22: ^{1}H NMR (400 MHz, $CDCl_3$) δ_H 4.14 (s, 1H), 3.90 (s, 1H), 3.69 (d, J = 3.9 Hz, 1H), 2.75 (dd, J = 17.5, 8.5 Hz, 1H), 2.52 (m, 1H), 2.23 (dt, J = 17.5, 8.5 Hz, 2H), 1.17 (d, J = 6.7 Hz, 3H); ^{13}C NMR (100 MHz, $CDCl_3$) δ_C 176.7, 87.3, 62.8, 37.2, 31.3, 17.9.

Statistical Analysis:

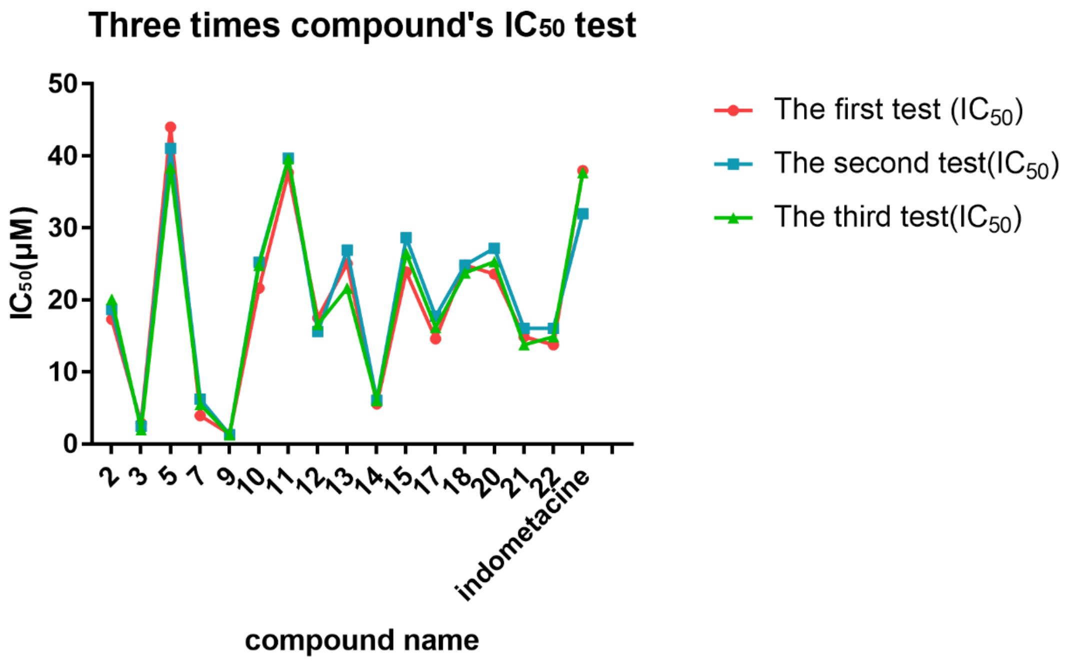

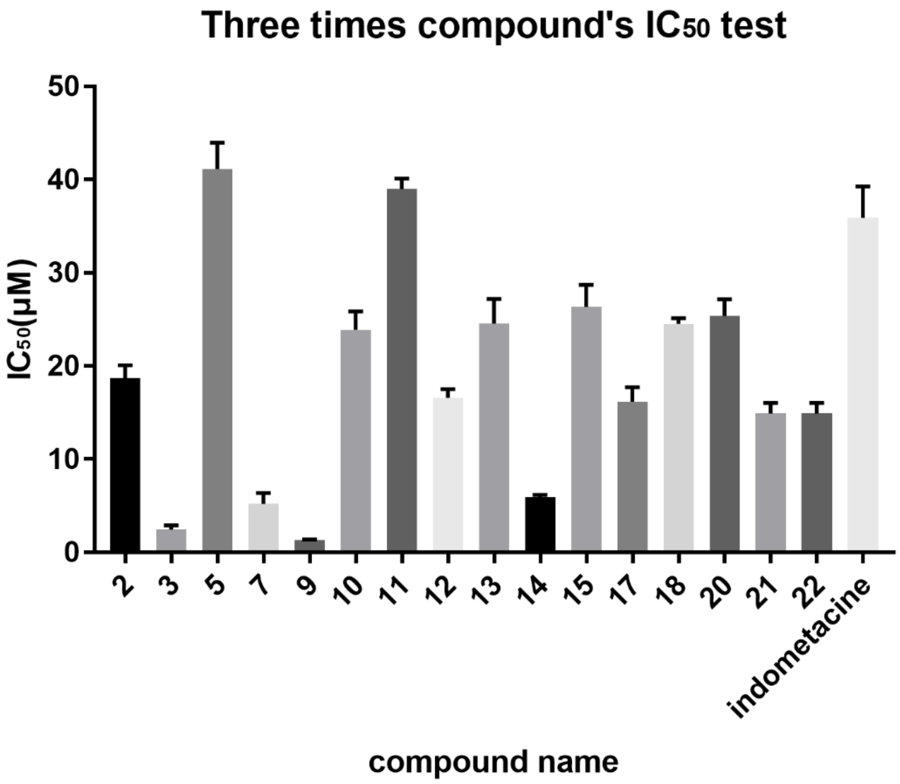

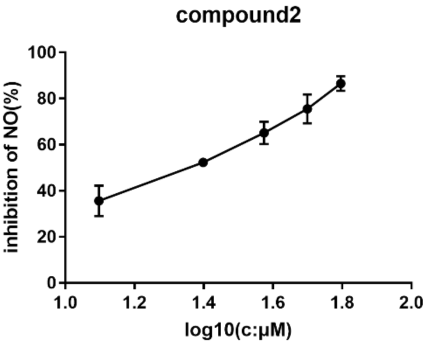
compound2
inhibition of NO(%)
0
20
40
60
80
100
1.0
1.2
1.4
1.6
1.8
2.0
log10(c:μM)

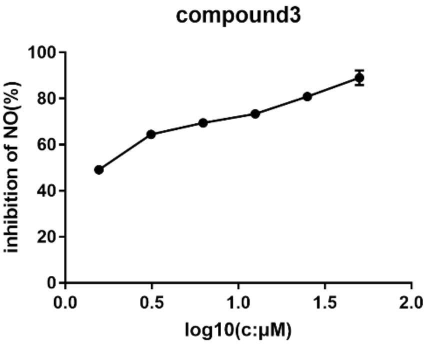
compound3
inhibition of NO(%)
0
20
40
60
80
100
0.0
0.5
1.0
1.5
2.0
log10(c:μM)

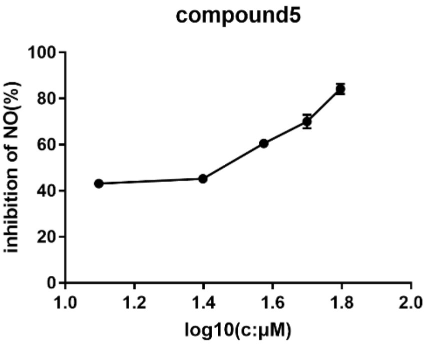
compound5
inhibition of NO(%)
0
20
40
60
80
100
1.0
1.2
1.4
1.6
1.8
2.0
log10(c:μM)

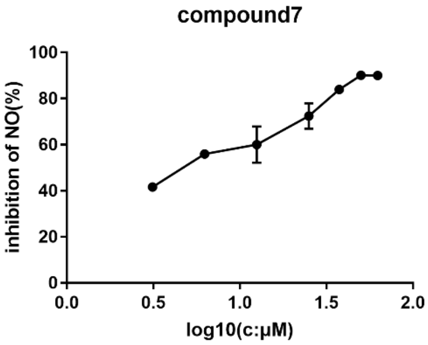
compound7
inhibition of NO(%)
0
20
40
60
80
100
0.0
0.5
1.0
1.5
2.0
log10(c:μM)

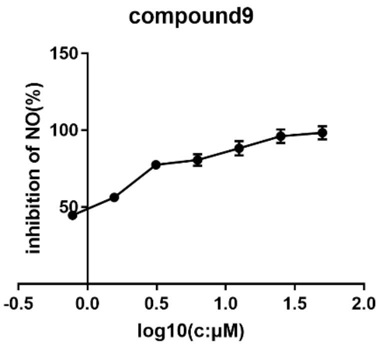
compound9
inhibition of NO(%)
50
100
150
-0.5
0.0
0.5
1.0
1.5
2.0
log10(c:μM)

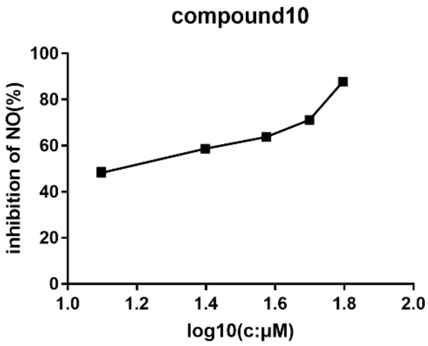
compound10
inhibition of NO(%)
0
20
40
60
80
100
1.0
1.2
1.4
1.6
1.8
2.0
log10(c:μM)

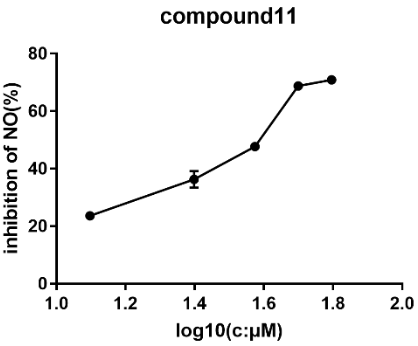
compound11
inhibition of NO(%)
0
20
40
60
80
1.0
1.2
1.4
1.6
1.8
2.0
log10(c:μM)

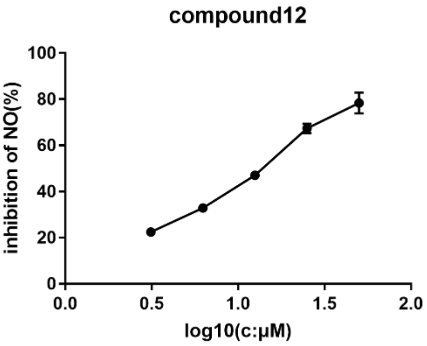
compound12
inhibition of NO(%)
0
20
40
60
80
100
0.0
0.5
1.0
1.5
2.0
log10(c:μM)

compound13

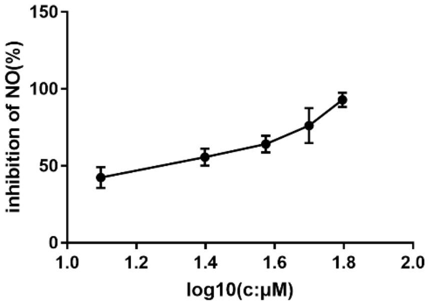

compound14

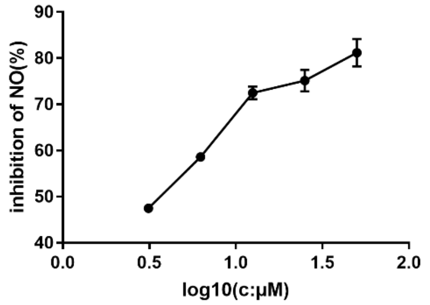

compound15

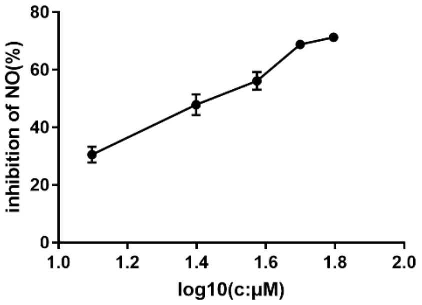

compound17

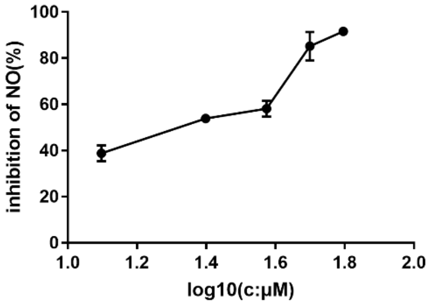

compound18

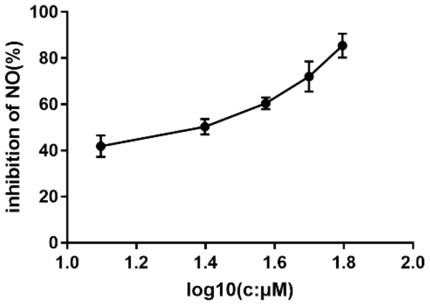

compound20

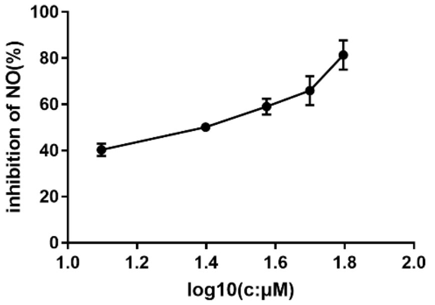

compound21

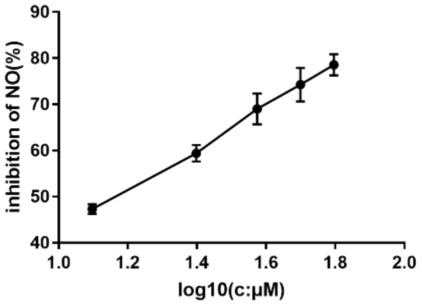

compound22

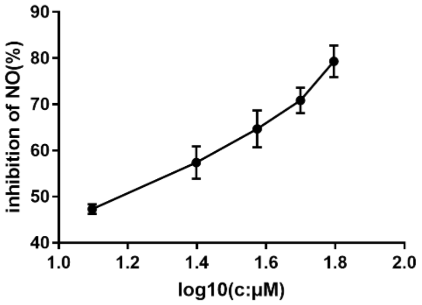

Supplement: Supplementary file 1 [file marinedrugs-18-00426-s001.pdf]
